# *Panax ginseng* ameliorates hepatorenal oxidative alterations induced by commercially used cypermethrin in male rats: experimental and molecular docking approaches

**DOI:** 10.1007/s11356-023-29935-2

**Published:** 2023-09-30

**Authors:** Samar S. Elblehi, Mona H. Hafez, Ali H. El-Far

**Affiliations:** 1 Department of Pathology, Faculty of Veterinary Medicine, Alexandria University, Alexandria, 22758 Egypt; 2 Department of Physiology, Faculty of Veterinary Medicine, Alexandria University, Alexandria, 22758 Egypt; 3 Department of Biochemistry, Faculty of Veterinary Medicine, Damanhour University, Damanhour, 22511 Egypt

**Keywords:** Antioxidant, Anti-inflammatory, Apoptosis, *Panax ginseng*, Cypermethrin

## Abstract

Cypermethrin (CYP) is a synthetic pyrethroid utilized as an insecticide in agriculture and various pest eradication programs. However, it induces numerous health hazards for animals and humans. Therefore, the current study used *Panax ginseng* root extract (ginseng) to reduce the hepatorenal damage caused by commercially used CYP. Thirty-two male Wistar albino rats were distributed into control, ginseng (300 mg/kg B.W/day), CYP (4.67 mg/kg B.W.), and Ginseng+CYP (rats received both CYP and ginseng). All treatments were administered orally for 30 consecutive days. Cypermethrin induced harmful effects on hepatic and renal tissues through a substantial decline in body weight in addition to a considerable increase in liver enzymes, functional renal markers, and cholesterol. Also, CYP significantly decreased acetylcholinesterase (AChE) activity and increased pro-inflammatory cytokines (interleukin-1*β* (IL-1*β*), IL-6, and tumor necrosis factor-*α* (TNF-*α*)). Moreover, a marked increase in malondialdehyde level with a significant drop in reduced glutathione level and total superoxide dismutase (T-SOD) and catalase (CAT) activities was reported in the CYP group in kidney and liver tissues. Additionally, CYP exhibited affinities to bind and inhibit AChE and antioxidant enzymes (T-SOD and CAT) in rats following the molecular docking modeling. The apparent hepatorenal oxidative damage was linked with obvious impairments in the liver and kidney histoarchitecture, immunohistochemical staining of B cell lymphoma-2 (Bcl-2), and caspase-3 proteins. *Ginseng* reduced CYP’s oxidative alterations by repairing the metabolic functional markers, improving antioxidant status, reducing the inflammatory response, and enhancing the molecular docking evaluation. It also ameliorated the intensity of the histopathological alterations and improved the immunohistochemical staining of Bcl-2 and caspase-3 proteins in the liver and kidney tissues. Finally, concomitant oral administration of ginseng mitigated CYP-prompted hepatorenal damage through its antioxidant, anti-inflammatory, and anti-apoptotic potentials.

**Graphical Abstract:**

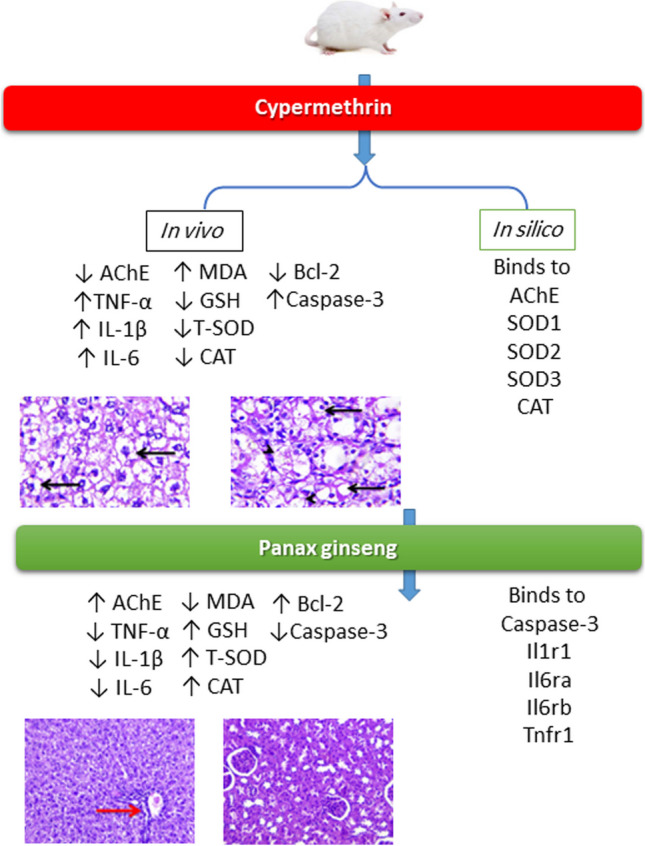

**Supplementary Information:**

The online version contains supplementary material available at 10.1007/s11356-023-29935-2.

## Introduction

Pesticides have been utilized worldwide for the last many decades to support the growth of the ever-enhancing human population by improving food production and for their defense against vector-borne diseases. The quantity of insecticides consumed on the crops is detrimental to the environment and animal and human health and might disrupt the normal physiological function and the histoarchitecture of various organs in the body. So, public concern increases over health and the environment (Shuklan et al. 2023). Problems that are associated with the low residual level of pesticides are blood disorders like anemia, defects in blood coagulation, nervous system damage, hepatic fibrosis, jaundice, allergic reactions, infertility, excretory problems, and genetic disorders (Özkara et al. 2016). Synthetic pyrethroids are a diverse class of more than a thousand powerful broad-spectrum organic insecticides used in domestic, veterinary, and agricultural applications. Cypermethrin (CYP), the synthetic pyrethroid insecticide, has long been utilized to control pests. Pyrethroids hurt reproductive fitness, feed conversion, and food safety in animals (Taniyasu et al. 2022). Also, human exposure to CYP has been reported to occur primarily occupationally during application or through residues in cow’s milk, bread, fruits, and vegetables (Sankar et al. 2012). CYP is widely used as an insecticide in developing countries to control pests. It is widely used in pest control programs in agriculture and public health in Egypt (Hussien et al. 2013). In vivo and in vitro research revealed that CYP brutally damaged DNA and caused a disruption in the prooxidant/antioxidant balance in the lymphocytes of rats (Ma et al. 2022). Cellular alterations associated with CYP are because of reactive oxygen species (ROS) generation and consequently oxidative stress (Abdul-Hamid et al. 2017; Abdou and Sayed 2019).

CYP was reported in many previous studies to have a harmful impact on hepatic and renal histoarchitectures and functions with imbalanced oxidant/antioxidant balance and molecular inflammatory cascade (Sankar et al. 2012; Abdou and Sayed 2019).

The root of *Panax ginseng* (Araliaceae) has been used as an herbal remedy for 2000 years and implemented in Chinese medicine (Liu et al. 2020). *Ginseng* has been consumed in East Asia as a food additive or herbal drug (Mostafa et al. 2021). *Ginseng* contains ginsenosides and gypenoside XVII (Yang et al. 2017; Liu et al. 2020). Also, the volatile oil in ginseng involves heterocycles, aldehydes, and sesquiterpenoid compounds (falcarinol) in volatile ginseng oil (Jian et al. 2011). The active compounds of *ginseng* have several biological actions, comprising anticancer, anti-inflammatory, antioxidant, and protective properties, as presented in Supplementary Files 1–11 that were retrieved from the CTD database (http://ctdbase.org/). In addition, *ginseng* has been included in many clinical trials as recognized in ClinicalTrials.gov (https://clinicaltrials.gov/) and presented in Supplementary File 12. Additionally, *P. ginseng* reduces inflammation and free radicals and prevents age-related conditions, chronic fatigue, and cardiovascular and digestive dysfunctions (Kim et al. 2017; Lee et al. 2017). The hepato-renoprotective effects of *P. ginseng* have been highlighted in many studies due to its anti-inflammatory, anti-apoptotic, and antioxidant attributes (Raheem et al. 2017; Yang et al. 2017; Mostafa et al. 2021). To our knowledge, no previous research work studied the potential protective impact of * ginseng* against CYP-induced hepatorenal damage. Consequently, the existing study was designed to investigate the ameliorative potential of * ginseng* against CYP-induced hepatorenal damages with a specific focus on the possible underlying mechanisms of these effects.

## Materials and methods

### Ethical approval

The experiment was permitted by the “Alexandria University-Institutional Animal Care and Use Committee” (ALEXU-IACUC) (Ethical Committee Approval Number: 2022/013/143), according to the “NIH Guide for the Care and Use of Laboratory Animals” guidelines.

### Chemicals

Powder of * ginseng* root extracts (Ginsana®, 100 mg soft gelatin capsules) was obtained from Egyptian International Pharmaceutical Industries Company (EIPICO, Ash Sharqia, Egypt). Cypermethrin (Cyperkill® 25% EC) was obtained from Chimac-Agriphar S.A Company (Belgium). All additional chemicals utilized were of the uppermost analytical grade.

### Determination of lethal dose 50 (LD50) of cypermethrin

Thirty-five rats were orally given CYP with seven concentrations (35, 40, 45, 50, 55, 60, and 65 mg of CYP in Cyperkill® per kg B.W.). Six rats were retained as a control group during the period of the experiment (7 days) and given 0.5 mL distilled water (vehicle of CYP) orally by a stomach tube. Mortality was evaluated and calculated in the different groups. LD50 was calculated following the Kerber formula (Pershin 1971).

$$\textrm{LD}50=\textrm{LD}100\hbox{--} \Sigma \left(z\times d\right)/n$$

where *z* is half the sum of the number of animals that died from two successive doses, *d* is the interval between doses, and *n* is the number of animals.

### Animals, housing conditions, and experimental design

Thirty-two male Wistar rats (8 weeks old) weighed an average of 150 ± 5 g were acquired from a closed breed colony at Pharos University, Alexandria, Egypt. Male rats were chosen to reduce the influence of hormonal variations associated with the estrous cycle in females. Animals were fed the basal diet, the standard feed requirement for rats (Table 1), and permitted free water access. They were retained beneath close supervision, mainly in metallic rat cages with a 12-h light-dark cycle and a temperature of 27 °C ± 2 for 14-day acclimatization previously to the beginning of the treatments. After the adaptation period, four experimental groups of rats were randomly and equally allocated (*n*= 8; three replicates).

**Table 1:** Ingredients of the basal diet

Ingredients	g/kg diet
Corn flour	529.5
Casein	200
Sucrose	100
Soybean oil	70
Cellulose	50
Mineral mix	35
Vitamin mix	10
L-Cystine	3
Choline	2.5

All groups were kept on the basal diet. The control group rats were orally intubated with 0.5 mL distilled water. The ginseng-treated group was orally intubated with * ginseng* at a concentration of 300 mg/kg bwt/day (Mostafa et al. 2021). CYP-treated group was orally intubated with 4.67 mg of CYP/kg bwt (1/10 (LD50)). Ginseng+ CYP-treated group was orally intubated with 300 mg of * ginseng* and 4.67 mg of CYP per kg bwt.

All treatments were administered orally daily using the stomach tube and continuously for thirty consecutive days. The doses administered throughout the experiment were adjusted to the biomass of each animal. The body weight of rats in all groups was recorded at the end of the experiment.

### Sampling

Rats were anesthetized with ketamine (100 mg/kg) and xylazine (10 mg/kg) injections on the last day of the experiment. Sera were taken from the blood collected from the orbital venous plexus for biochemical analyses and centrifuged at 3000 rpm for 15 min at 4 °C. Rats were decapitated; the livers and kidneys were collected and weighed and then rinsed in ice-cold physiological saline (NaCl 0.9%, pH 7).

Apart from the liver and the left kidney of each rat were clipped off and rinsed with physiological saline solution and deionized water. Tissues were blotted with blotting paper and perfused with 50 mM sodium phosphate saline buffer (100 mM Na_2_HPO_4_/NaH_2_PO_4_, pH 7.4) in an ice-cold medium which contains 0.1 mM EDTA to get rid of RBC and platelet gatherings. Formerly, tissues were shredded in 10 mL of ice-cold buffer/gram tissue, homogenized, and centrifuged at 10,000×g for 30 min at 4 °C. The supernatants of the tissue homogenates were relocated into Eppendorf tubes and kept at −80 °C until oxidative/nitrosative and anti-oxidative enzyme activities were investigated. A 10% neutral-buffered formalin was prepared to reserve the tissues for histopathological investigation. Other liver and left kidney slices were held at −20 °C to analyze oxidative stress markers.

### Biochemical analyses

Serum aspartate aminotransferase (AST) (DD0453) and alanine aminotransferase (ALT) (DD0440) enzymes were determined using Diamond Diagnostics kits (Cairo, Egypt) according to the method of Reitman and Frankel (1957) which rely on the transfer of an amino group from aspartate/alanine to α-ketoglutarate results in the generation of glutamate, resulting in the production of a colorimetric (450 nm) product proportional to the AST/ALT enzymatic activity present. Serum total protein and albumin levels were evaluated following the Doumas et al. (1981) procedure. In addition, globulin values in the serum were calculated by subtracting the albumin values from the sample’s total protein concentrations.

Serum creatinine (B6021) levels were determined following Bartels et al. (1972) and urea (B6025) levels were determined according to Coulombe and Favreau (1963). In this assay, creatinine/urea concentration is evaluated by a coupled enzyme reaction, which results in a colorimetric (570 nm)/fluorometric (λex = 535em/λ = 587 nm) product, proportional to the creatinine/urea present. Creatinine and urea levels were assessed using commercially available kits (Biodiagnostics Co., Giza, Egypt). Colorimetric kits from Boehringer Mannheim were utilized to quantify total serum cholesterol (TC) and triacylglycerol (TAG) (Mannheim, Germany). Total cholesterol/TAG concentration is determined by a coupled enzyme assay, which results in a colorimetric (570 nm)/fluorometric (λex = 535/λem = 587 nm) product, proportional to the cholesterol/TAG present.

### Enzyme-linked immunosorbent assay (ELISA) assessments

Serum acetylcholinesterase (AChE) activities were evaluated by ELISA kit (NB00187), NOVA (Bioneovan Co., Beijing, China); this assay is an optimized version of the Ellman method in which thiocholine, produced by AChE, reacts with 5,5′-dithiobis(2-nitrobenzoic acid) to form a colorimetric (412 nm) product, proportional to the AChE activity present (Worek et al. 2012). Also, serum tumor necrosis factor-*α* (TNF-*α*) (Seriolo et al. 2006) and interleukin-1*β* (IL-1*β*) and -6 (IL-6) (Safieh-Garabedian et al. 1995) were determined with ELISA kits following the manufacturer’s instructions (Millipore, CA, USA). The values of cytokines were determined by the optical densitometry determination at 450 nm using a microplate reader. An ELISA Plate Reader (Bio-Rad, Hercules, CA, USA) was used to finalize all readings.

### Oxidant stress and antioxidant status assessments

Malondialdehyde (MDA), the product of lipid peroxidation, was determined by spectrophotometry following Ohkawa et al. (1979); briefly, sample was mixed with 2 mL TBA reagent, and the tubes were placed in a hot water bath for 10 min and cooled at room temperature followed by centrifugation; the supernatant was used for spectrophotometric assessment at 532 nm against a reference blank. Reduced glutathione (GSH) levels were determined following the method of Ellman (1959), where a yellow chemical spectrophotometrically was determined at 405 nm after GSH reduction of 5,5′-dithiobis(2-nitrobenzoic acid). Total superoxide dismutase (T-SOD) was determined following Sun et al. (1988), which depends on the formation of a colored complex due to auto-oxidation of pyrogallol. This was measured for 3 min at an interval of 60 s at 412 nm in the presence or absence of the enzyme. Catalase (CAT) activity was examined according to Aebi (1984) which depends on the reducing absorbance of hydrogen peroxide (H_2_O_2_) consumption measured at 240 nm at an interval of 60 s for 3 min. Levels of protein in hepatic and renal homogenates were assessed by Lowry et al. (1951).

### Histopathological examination and semi-quantitative lesion scoring

After necropsy, tissue specimens from seven rats’ livers and right kidneys per group were quickly dissected and delicately rinsed with normal saline. They were immediately fixed for at least 24 h in phosphate-buffered formalin (10%, pH 7.4) and processed via the conventional paraffin embedding technique (Bancroft and Layton 2013). The specimens were embedded in paraffin blocks, 5-μm slices were obtained from them, mounted on slides, deparaffinated with xylene, and rehydrated with ethanol. Seven slides per organ for group were stained with Mayer’s hematoxylin and eosin (H&E) for further histopathological investigations. Tissue slides were inspected using a light microscope (Leica, DM500) at magnifications of ×100 and ×400 and captured with a digital camera (EC3, Leica, Germany). For grading the histopathological variations detected in the livers and kidneys, the following semi-quantitative scoring system was utilized: (−) absence of the lesion = 0%, (+) mild = 5–25%, (++) moderate = 26–50%, and (+++) severe damage ≥ 50% of the examined tissue sections were involved (Mahana et al. 2023). Seven slides per group were examined to assess the severity of the histopathological alterations in each organ depending on the number of affected slides and regions within the same slide. To avoid any bias, the histopathological assessment was conducted blindly.

### Immunohistochemical assessment of Bcl-2 and caspase-3 proteins

Following embedding in paraffin blocks, liver and kidney tissue slices were cut at 4 μm thicknesses and mounted on positively charged slides. Immunohistochemical staining was conducted for marking B cell lymphoma-2 (Bcl-2) and caspase-3 proteins (Missaoui et al. 2014). The slices were deparaffinized and rehydrated and underwent antigen retrieval with 10 mM citrate buffer (pH 6.0) in the microwave for 10 min. Then, endogenous peroxidase activity was blocked with 3% H_2_O_2_ for 10 min. Nonspecific proteins were inhibited by 2% bovine serum albumin. The slices were rinsed three times in Dako Tris-buffered saline before being incubated overnight at 4 °C with a primary rabbit polyclonal anti-Bcl-2 antibody (1:100) (PA5-27094; Thermo Fisher Scientific, WA, USA) and rabbit polyclonal anti-caspase-3 antibody (1:100) (Code# ab4051; Abcam, Cambridge, UK). The tissue slices were rinsed in Tris-buffered saline and then incubated with the streptavidin-horseradish peroxidase reagent and a biotinylated secondary antibody for 30 min at 37 °C. To establish a peroxidase reaction, the sections were washed with washing buffer and incubated with 3,3-diaminobenzidine tetrahydrochloride (DAB Substrate Kit, Thermo Fisher Scientific). Finally, the tissue sections were counterstained with Mayer’s hematoxylin to enhance the nuclear staining and mounted with DPX. The stained cells were examined using a light microscope and digitally photographed. Images of ten different fields at a magnification of ×400 were analyzed using ImageJ software (ImageJ software, v1.46r, National Institutes of Health, USA) to calculate the area (%) of Bcl-2 and caspase-3 protein-positive brown immune-stained cells using one section from each animal.

### Molecular docking

The three-dimensional protein structures were retrieved from AlphaFold Protein Structure Database (https://alphafold.ebi.ac.uk/). Furthermore, the three-dimensional structures of CYP and * ginseng*’s active compounds were retrieved from the PubChem (https://pubchem.ncbi.nlm.nih.gov/) and LOTUS: Natural Products databases (https://lotus.naturalproducts.net/).

Molecular docking scores of CYP against rat’s AChE, Cu/Zn-superoxide dismutase (SOD1), Mn-superoxide dismutase (SOD2), extracellular superoxide dismutase (SOD3), and CAT were assessed using Molecular Operating Environment (MOE 2015.10, Chemical Computing Group, Montreal, QC, Canada) software with induced fit method. Also, molecular docking scores of * ginseng* active compounds against rat’s caspase-3, interleukin-1 alpha receptor-1 (Il1r1), interleukin-6 receptor subunit alpha (Il6ra), interleukin-6 receptor subunit beta (Il6rb), and tumor necrosis factor receptor 1 (Tnfr1) were assessed using MOE software with induced fit method. Finally, visualization of ligand-protein interactions was done using MOE software.

### Statistical analysis

A one-way ANOVA with Tukey’s post hoc multiple range tests was utilized for data analysis using GraphPad Prism v.9 (https:// www. graph pad. com/) (GraphPad, San Diego, CA, USA). All statements of significance were at *P* < 0.05.

## Results

### Effect of cypermethrin and *Panax ginseng* on body and hepatorenal weights

**Fig. 1:**
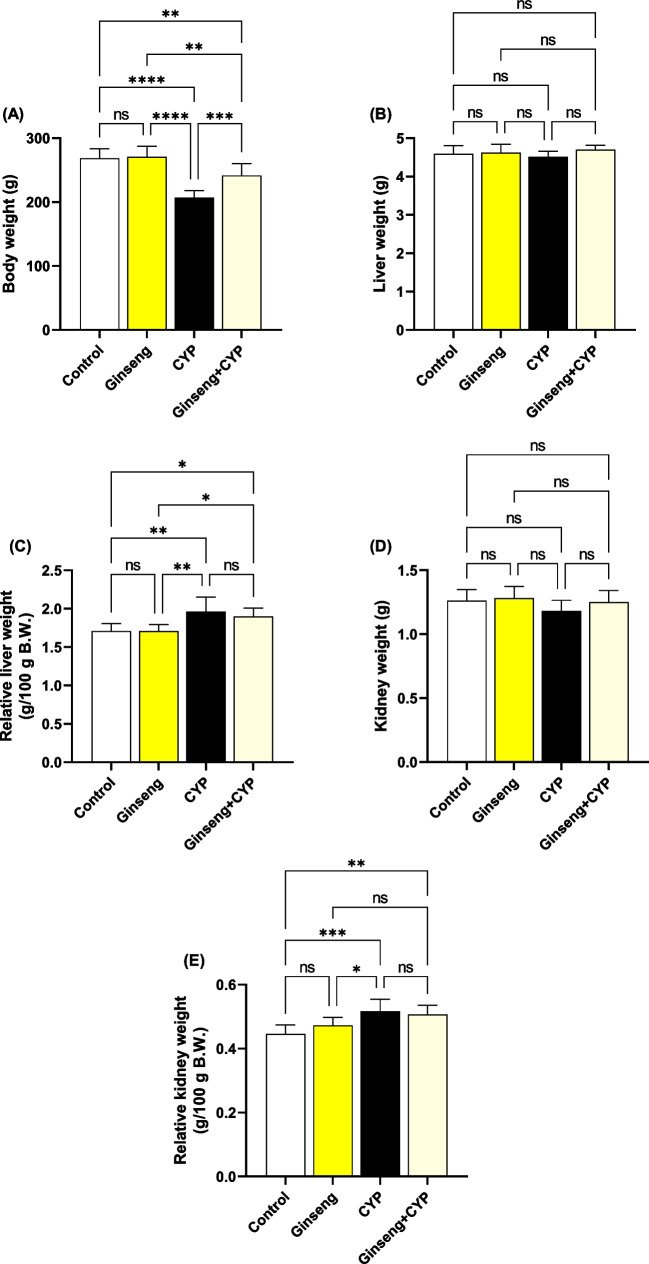
Assessment of body, liver, and kidney weights. **A** Body weight. **B** Liver weight. **C** Relative liver weight. **D** Kidney weight. **E** Relative kidney weight. Data were analyzed with a one-way ANOVA followed by Tukey’s multiple comparison test. Data are expressed as the mean ± SD; *n*=8. Means within columns carrying * are significantly different at *p* <0.05, **p < 0.01, ***p < 0.001, and ****p < 0.0001. CYP, cypermethrin

Data represented in Fig. 1A, weights of the body of rats in CYP group, were considerably declined compared with ginseng (*p* < 0.0001), Ginseng+CYP (*p* < 0.001), and control (*p* < 0.0001) groups. The weights of the liver and kidney were within physiologically standard ranges (Fig. 1B, D). The CYP group markedly increased relative kidney and liver weights (Fig. 1C, E).

### Effect of cypermethrin and *Panax ginsen*g on liver and kidney functional serum markers

**Fig. 2:**
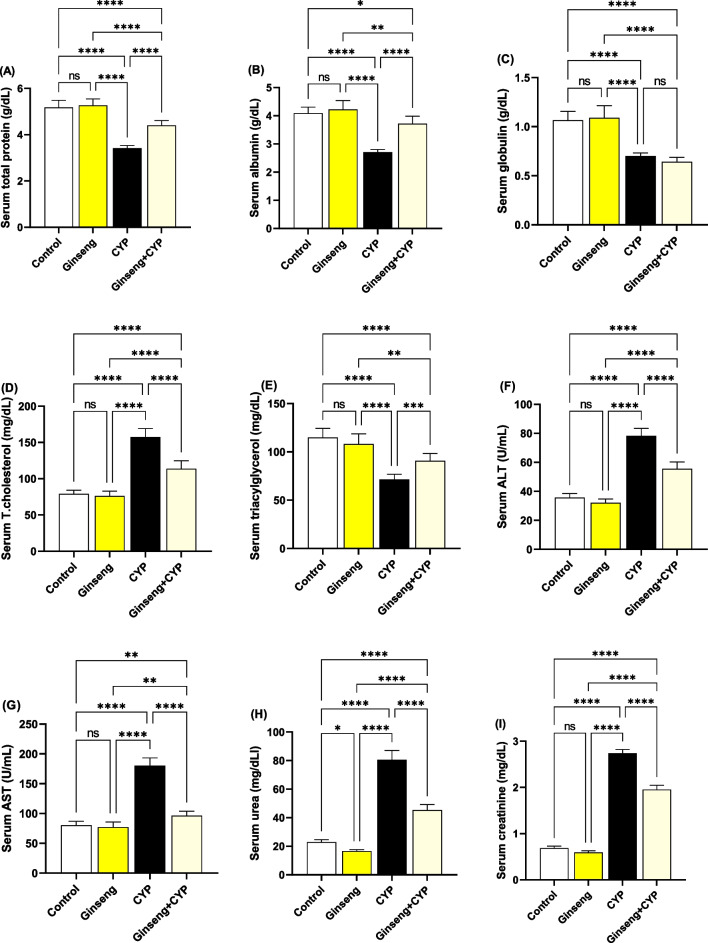
Serum biochemical markers. **A** Total protein. **B** Albumin. **C** Globulin. **D** Total cholesterol. **E** Triacylglycerol (TAG). **F** Alanine aminotransferase (ALT). **G** Aspartate aminotransferase (AST). **H** Urea. **I** Creatinine. Data were analyzed with a one-way ANOVA followed by Tukey’s multiple comparison test. Data are expressed as the mean ± SD; *n*=8. Means within columns carrying * are significantly different at *p* <0.05, **p < 0.01, ***p < 0.001, and ****p < 0.0001. CYP, cypermethrin

Results presented in Fig. 2A–I showed significant (*p* < 0.0001) increases in serum T. cholesterol, ALT, AST, urea, and creatinine relative to the control and ginseng groups. Meanwhile, the serum TP, albumin, and TAG levels considerably decreased (*p* < 0.0001). The same parameters were markedly (*p* < 0.0001) decreased in Ginseng+CYP excluding globulin, while TAG values were considerably (*p* < 0.001) increased relative to the CYP-treated rats.

### Effect of cypermethrin and *Panax ginseng* on serum acetylcholinesterase

**Fig. 3:**
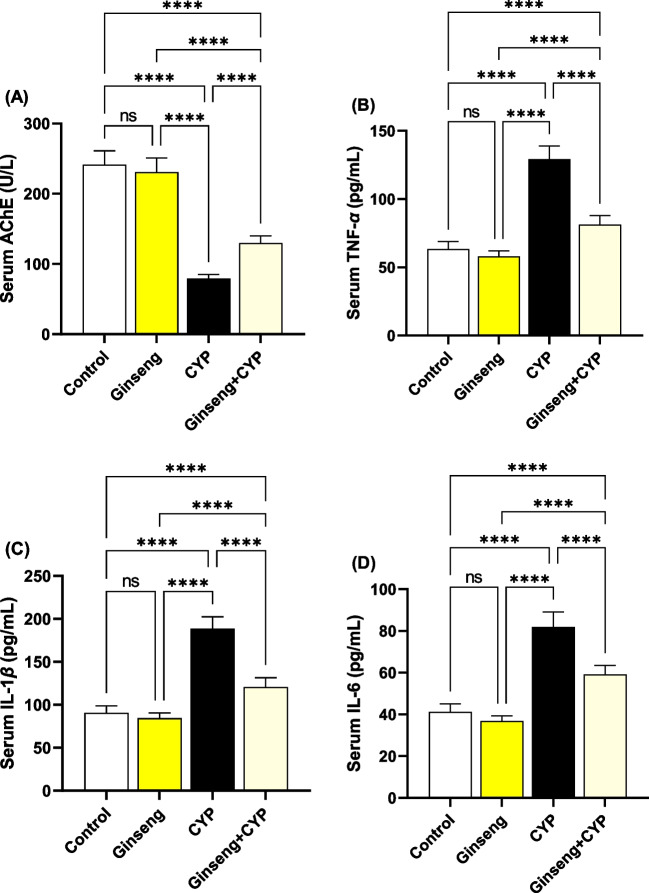
Serum acetylcholinesterase and inflammatory markers. **A** Acetylcholinesterase (AChE). **B** Tumor necrosis factor-*α* (TNF-*α*). **C** Interleukin-1*β* (IL-1*β*). **D** Interleukin-6 (IL-6). Data were analyzed with a one-way ANOVA followed by Tukey’s multiple comparison test. Data are expressed as the mean ± SD; *n*=8. Means within columns carrying * are significantly different at *p* <0.05, **p < 0.01, ***p < 0.001, and ****p < 0.0001. CYP, cypermethrin

Serum AChE activity was reduced significantly (*p* < 0.0001) in the CYP group relative to other treated rats. Supplementation of * ginseng* and CYP in Ginseng+CYP markedly (*p* < 0.001) improved the AChE activity close to the CYP group (Fig. 3A).

### Effect of cypermethrin and *Panax ginseng* serum pro-inflammatory cytokines

Serum TNF-*α* (Fig. 3B), IL-1*β* (Fig. 3C), and IL-6 (Fig. 3D) concentrations were significantly (*p* < 0.0001) enhanced in CYP rats relative to the control, ginseng, and Ginseng+CYP-treated rats. In contrast, their values markedly declined in Ginseng+CYP relative to the CYP group.

### Effect of cypermethrin and *Panax ginseng* on hepatorenal oxidative stress and antioxidant status

**Fig. 4:**
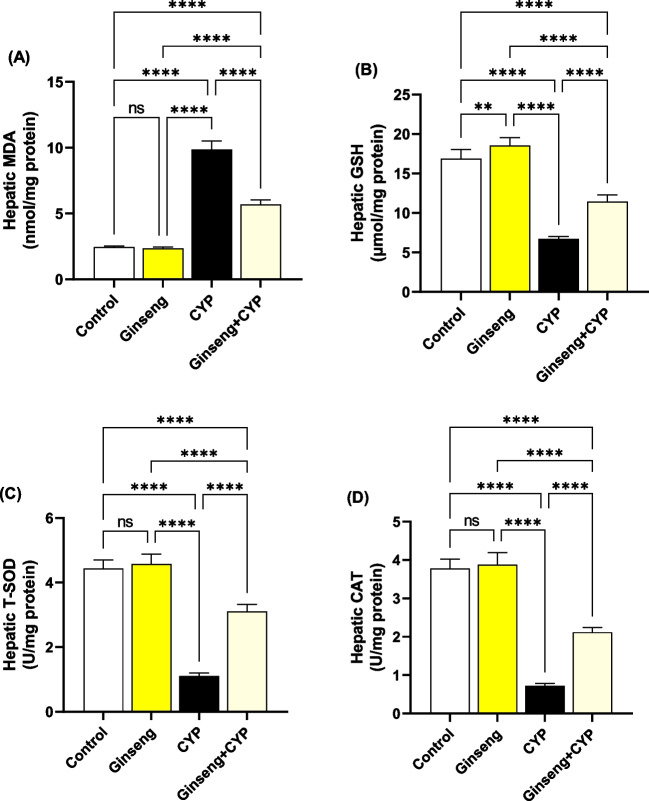
Hepatic oxidative stress and antioxidant status. **A** Malondialdehydes (MDA). **B** Reduced glutathione (GSH). **C** Total superoxide dismutase (T-SOD). **D** Catalase (CAT). Data were analyzed with a one-way ANOVA followed by Tukey’s multiple comparison test. Data are expressed as the mean ± SD; *n*=8. Means within columns carrying * are significantly different at *p* <0.05, **p < 0.01, ***p < 0.001, and ****p < 0.0001. CYP, cypermethrin

**Fig. 5:**
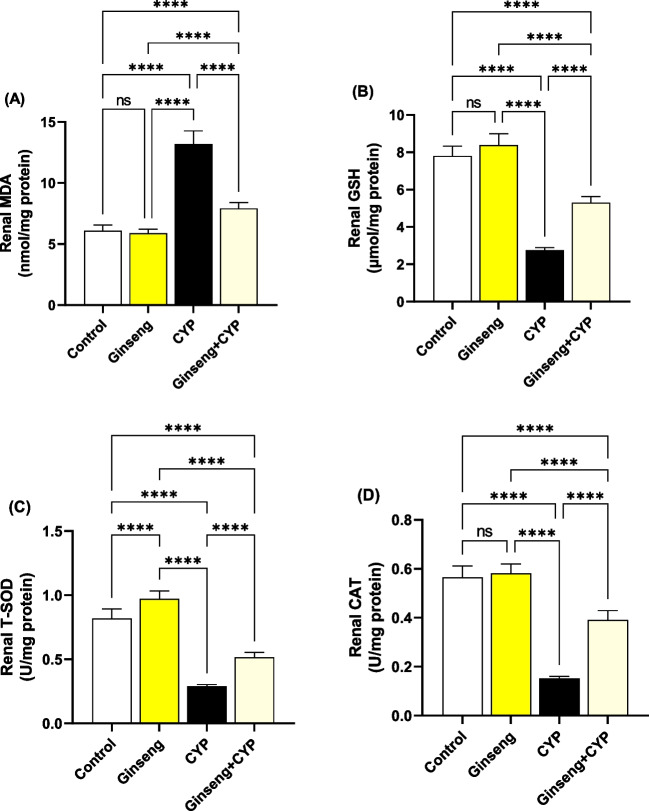
Renal oxidative stress and antioxidant status. **A** Malondialdehydes (MDA). **B** Reduced glutathione (GSH). **C** Total superoxide dismutase (T-SOD). **D** Catalase (CAT). Data were analyzed with a one-way ANOVA followed by Tukey’s multiple comparison test. Data are expressed as the mean ± SD; *n*=8. Means within columns carrying * are significantly different at *p* <0.05, **p < 0.01, ***p < 0.001, and ****p < 0.0001. CYP, cypermethrin

MDA levels considerably (*p* < 0.0001) enhanced in CYP-treated rats relative to control and ginseng-treated rats, while the T-SOD and CAT activities and GSH concentration were markedly (*p* < 0.0001) decreased in the liver (Fig. 4A–C) and kidney (Fig. 5A–C) homogenates. Hepatic GSH values and renal T-SOD activities were considerably improved in ginseng relative to control rats.

### Effect of cypermethrin and *Panax ginseng* on hepatorenal histoarchitectures

**Fig. 6:**
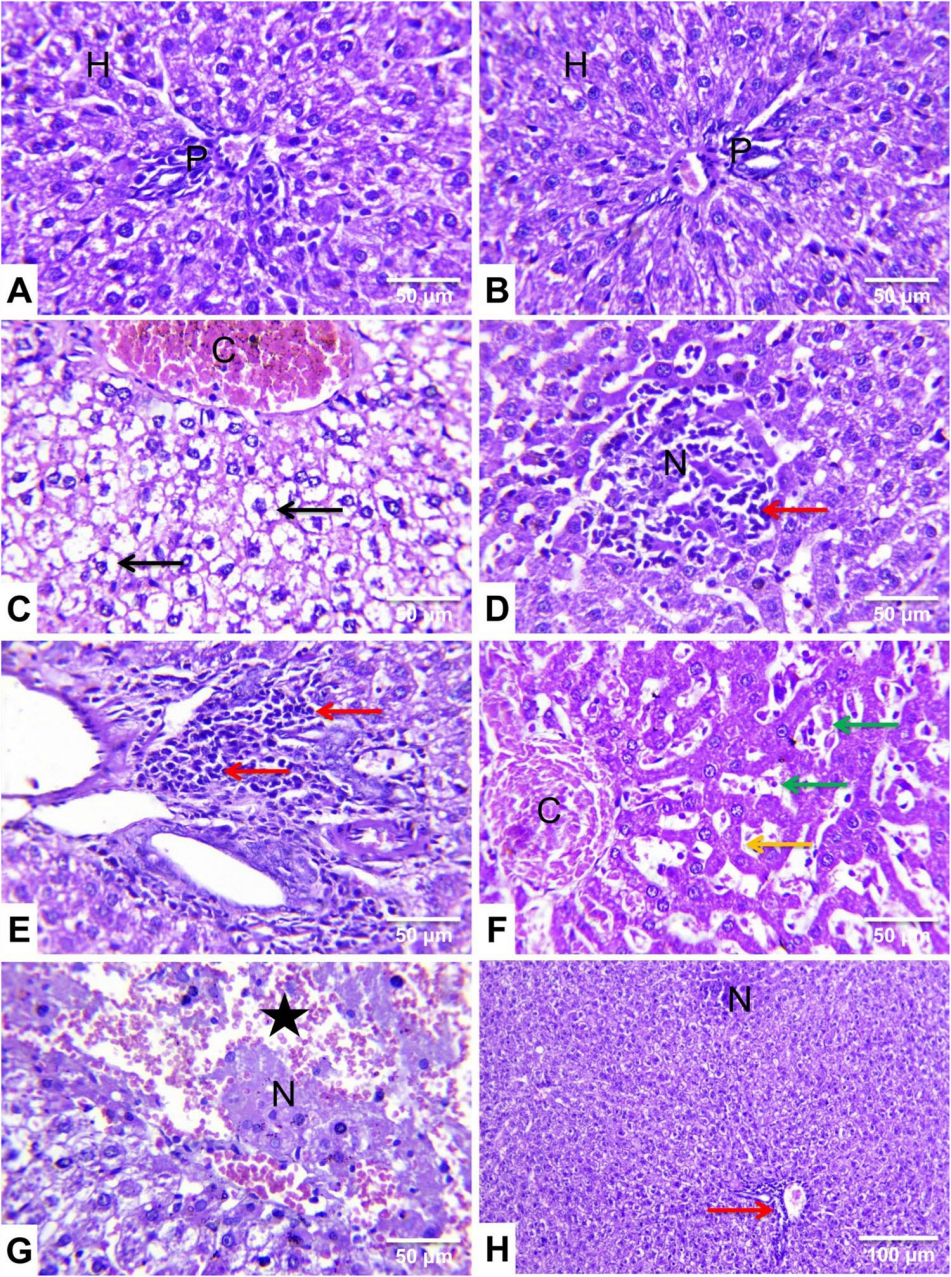
Representative photomicrographs of rat liver sections from experimental groups (H&E, ×400 (**A–G**) and ×100 (**H**)). **A** A control rat and **B** *Panax ginseng* (ginseng)-treated rat showing normal histoarchitecture of the hepatocytes (H) and portal areas (P). **C**, **D**, **E**, **F**, and **G** Cypermethrin (CYP)-treated rats showing diffuse cytoplasmic vacuolation of the hepatocytes of hydropic type (black arrow), mid-zonal hepatocellular necrosis (N), mononuclear cells infiltrations (red arrow), congestion of the central vein (C), and hepatic sinusoids (green arrow), atrophied hepatic cords (yellow arrow), and extravasated RBCs (star). **H** A Ginseng+ CYP-treated rat showed marked improvement in hepatic histoarchitecture with minute areas of hepatocellular necrosis (N) and few mononuclear cells infiltrations in the portal area (red arrow)

The livers of the control (Fig. 6A) and ginseng-treated (Fig. 6B) rats revealed normal histological limits with large polygonal hepatocytes arranged in cords and divided by blood sinusoids, central vein, and portal triad with few inflammatory cell infiltrations.

Meanwhile, CYP-treated rats’ hepatic sections showed diffuse cytoplasmic vacuolation of the hepatocytes of mostly hydropic (Table 2 and Fig. 6C) and lipid types. Periportal and mid-zonal (Table 2 and Fig. 6D) hepatocellular necrosis with mononuclear inflammatory cells infiltrations were constant findings. In addition, the portal areas showed intense mononuclear cells infiltrations (Table 2 and Fig. 6E). Widening of the hepatic sinusoids, hyperactivation of Kupffer cells with atrophy of the hepatic cords, widespread severe vascular congestion (Table 2 and Fig. 6F), and areas of hemorrhage (Table 2 and Fig. 6G) also were noticed.

**Table 2:** Lesion scoring in the hematoxylin and eosin-stained liver and kidney tissue sections in different experimental groups

	Control	Ginseng	CYP	Ginseng+ CYP
Liver				
Hepatocytes				
Vacuolation	−	−	+++	++
Necrosis	−	−	++	+
Inflammatory cells infiltrations	−	−	++	+
Vascular congestion	−	−	+++	+
Portal inflammation	−	−	++	+
Kidney				
Tubular epithelial				
Vacuolation	−	−	+++	++
Necrosis	−	−	++	+
Intratubular cast formation	−	−	++	+
Glomerular necrosis	−	−	++	+
Interstitial Inflammatory cells infiltrations	−	−	++	+
Vascular congestion	−	−	+++	++
Perivascular edema	−	−	++	+
Perivascular inflammatory cells infiltration	−	−	++	+

Lesion scoring: (−) absence of the lesion = 0%, (+) mild = 5–25%, (++) moderate = 26–50%, and (+++) severe ≥50% of the examined tissue sections were involved. *n*= 7

*Ginseng Panax ginseng*, *CYP* cypermethrin, *Ginseng+ CYP* cypermethrin plus *Panax ginseng*-treated

In contrast, the hepatic histoarchitecture of the Ginseng+ CYP-treated group was significantly improved, as the previously described lesions were less in the severities and distributions (Table 2 and Fig. 6H).

**Fig. 7:**
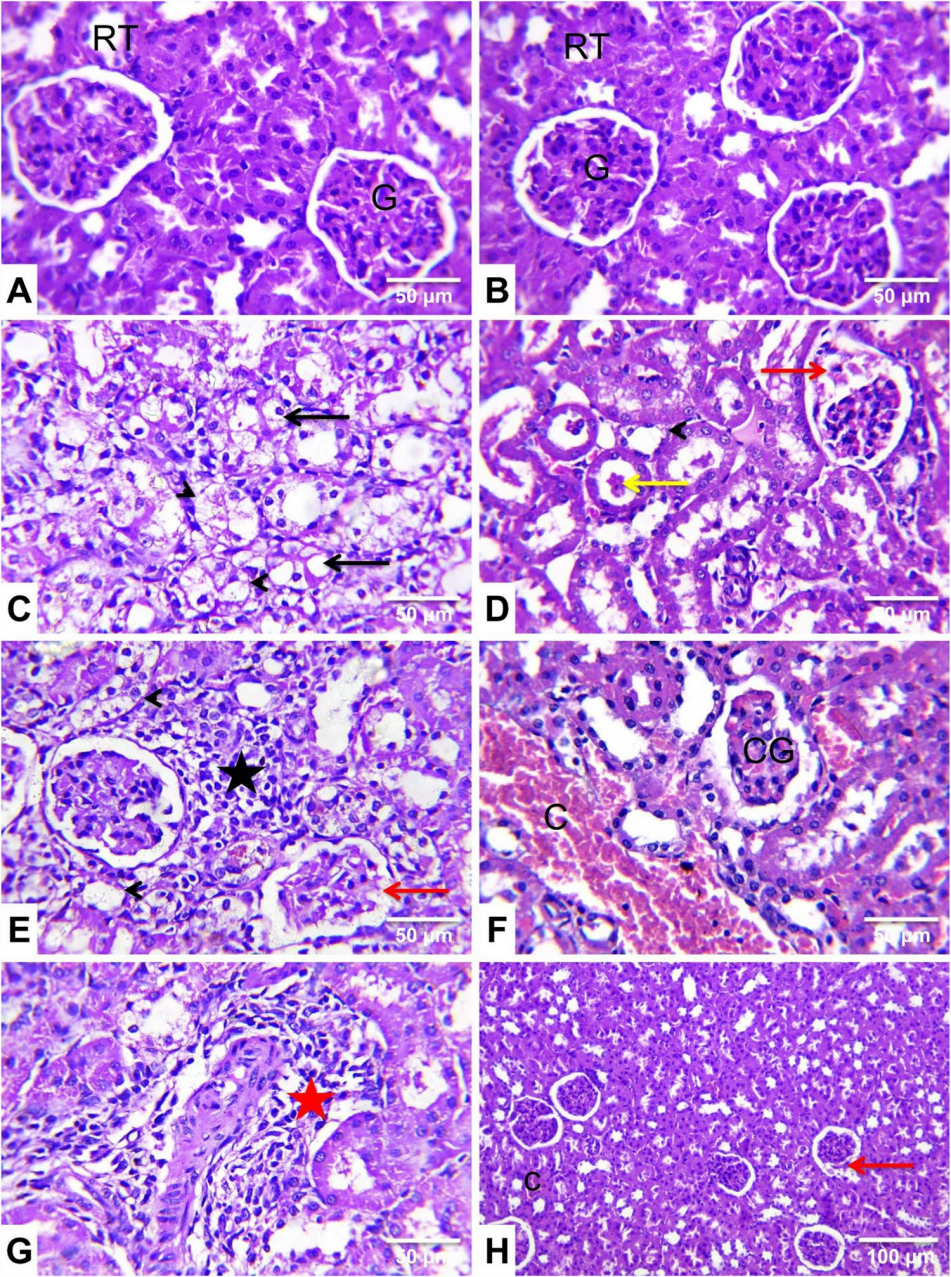
Representative photomicrographs of rat kidney sections from experimental groups (H&E, ×400 (**A–G**) and ×100 (**H**)). **A** A control rat and **B** *Panax ginseng* (ginseng)-treated rat showing normal histoarchitecture of glomeruli (G) bowman’s space, renal tubules (RT), and interstitial tissue. **C**, **D**, **E**, **F**, and **G** Cypermethrin (CYP)-treated rats showing vacuolated tubular epithelium (black arrow), attenuated and necrotic tubular epithelium (arrowhead), dark eosinophilic necrotic debris (yellow arrow) in the tubular lumen, necrotic glomerular capillary tufts (red arrow), tubulo interstitial nephritis with mononuclear cells infiltrations in the renal cortex (black star), congested blood vessels (C), congested glomerular capillary tufts (CG), and perivascular inflammatory cells infiltration (red star). **H** A Ginseng + CYP-treated rat showed marked improvement of the kidney histoarchitecture with few glomeruli showing necrotic capillary tufts (red arrow) and mild vascular congestion (C)

The kidneys of the control (Fig. 7A) and ginseng-treated (Fig. 7B) rats revealed the normal histological architecture of the renal parenchyma with clearly defined tubules and glomeruli.

Approximately 80% of the CYP-treated rats inspected renal tubules exhibited severe degenerative alterations, with the tubular epithelial cells being moderately swollen, had a foamy granular cytoplasm, and enlarged toward the tubular lumen, causing the lumen to be narrow and star-shaped. Moderate cytoplasmic vacuolation (Table 2 and Fig. 7C) was observed in other tubular epithelial cells. Tubular epithelium attenuation and necrosis with pyknotic or karyorrhectic nuclei were noticed. The tubular lumen contains dark eosinophilic necrotic debris (Table 2 and Fig. 7D). There were compressed and necrotic glomerular capillary tufts with a widening of Bowman’s space. The parenchyma of the renal cortex showed tubulointerstitial nephritis with lymphocytic cell infiltrations (Table 2 and Fig. 7E). Also, there were vascular and glomerular congestion (Table 2 and Fig. 7F), moderate perivascular lymphocytic cell infiltrations (Table 2 and Fig. 7G), edema, and focal areas of hemorrhages.

On the other hand, the lesions in the kidneys of Ginseng+ CYP-treated rats resembled those in the CYP-treated rats; however, they were less in severity and distribution (Table 2 and Fig. 7H).

### Effect of cypermethrin and *Panax ginseng* on hepatic and renal Bcl-2 and caspase-3 immunohistochemical staining

**Fig. 8:**
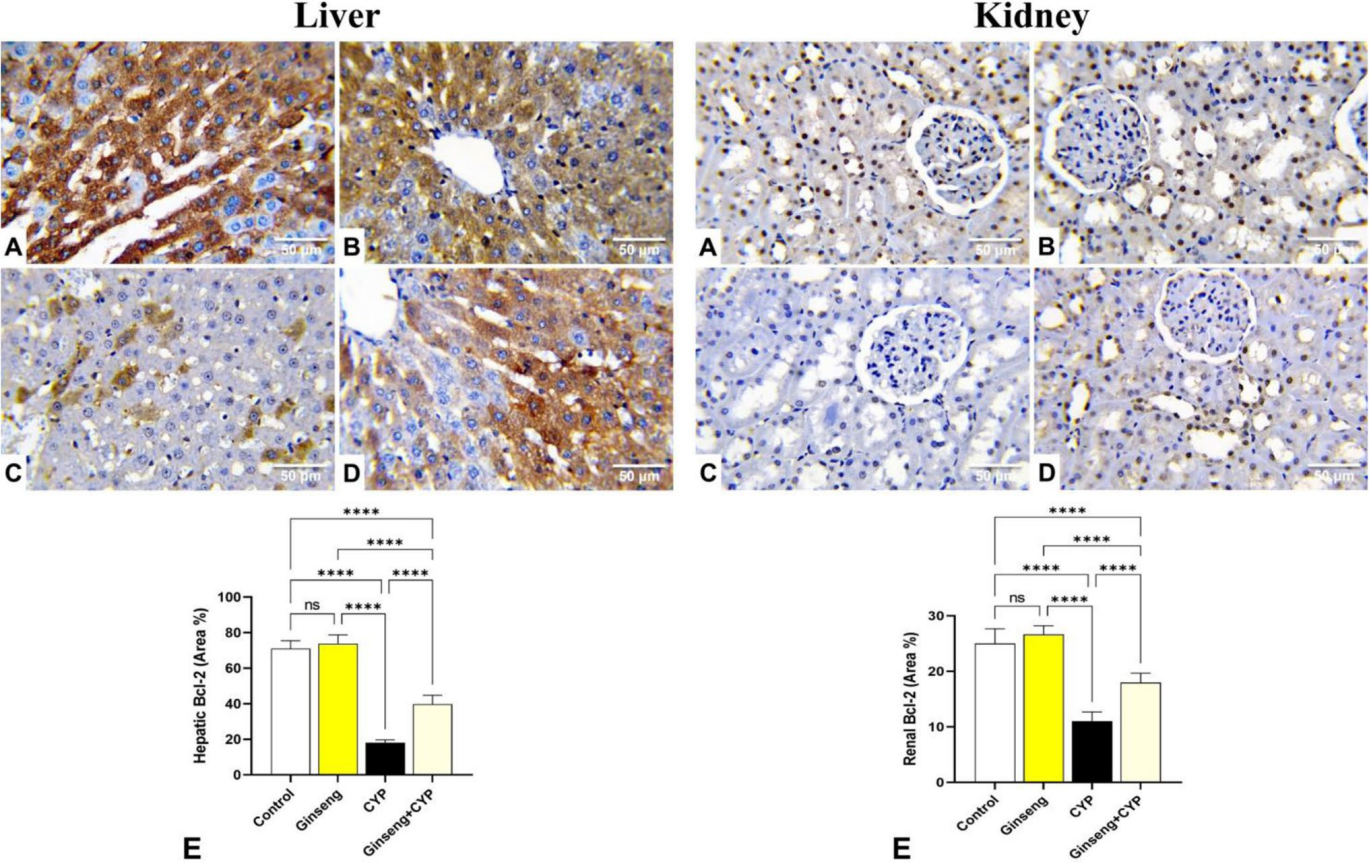
Representative photomicrographs demonstrating the immunohistochemical staining (brown staining) of the B-cell lymphoma-2 (Bcl-2) in the experimental rats’ liver and kidney tissue sections (IHC, ×400). **A** Control, **B** *Panax ginseng* (ginseng)-treated, **C** cypermethrin (CYP)-treated, and **D** Ginseng + CYP-treated rats. **E** Quantification of Bcl-2 expression, the immunohistochemical staining of Bcl-2 was measured as area percent (%) across 10 different fields/section, *n* = 7 rat/group. Data were analyzed with a one-way ANOVA followed by Tukey’s multiple comparison test. Data are expressed as the mean ± SD; *n*=7. Means within columns carrying * are significantly different at *p* <0.05, **p < 0.01, ***p < 0.001, and ****p < 0.0001

**Fig. 9:**
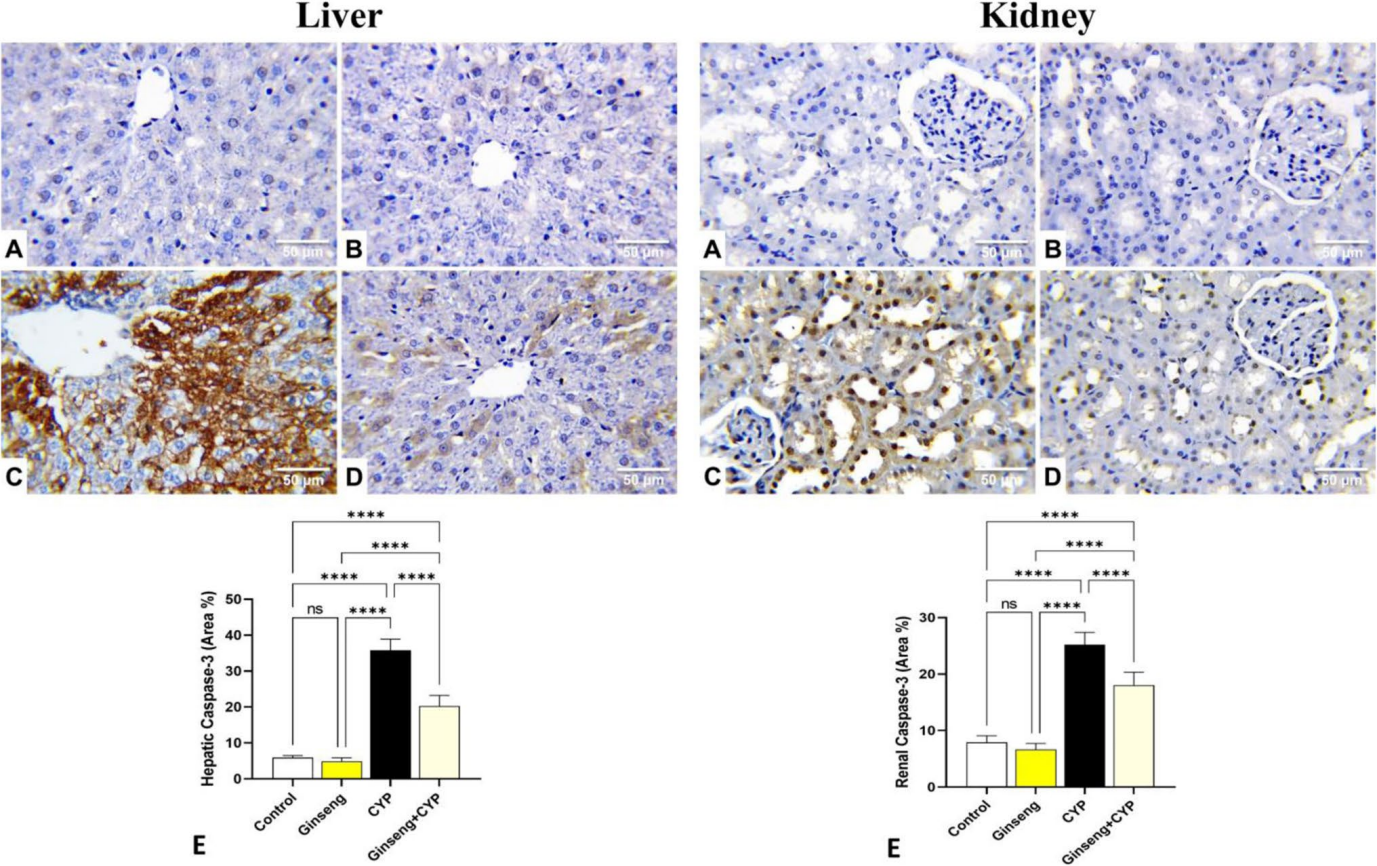
Representative photomicrographs demonstrating the immunohistochemical staining (brown staining) of caspase-3 in the experimental rats’ liver and kidney tissue sections (IHC, ×400). **A** Control, **B** *Panax ginseng* (ginseng)-treated, **C** cypermethrin (CYP)-treated, and **D** Ginseng+ CYP-treated rats. **E** Quantification of caspase-3 expression, the immunohistochemical staining of caspase-3 was measured as area percent (%) across 10 different fields/sections, *n*= 7 rat/group. Data were analyzed with a one-way ANOVA followed by Tukey’s multiple comparison test. Data are expressed as the mean ± SD; *n*= 7. Means within columns carrying * are significantly different at *p* <0.05, **p < 0.01, ***p < 0.001, and ****p < 0.0001

Immunohistochemical staining of the hepatic and renal tissues for Bcl-2 and caspase-3 consistently with the quantitative assessment demonstrated that normal control and ginseng-treated rats displayed strong positive immunostaining of Bcl-2 (Fig. 8A, B) and weak immunostaining of caspase-3 (Fig. 9A, B). There were no significant alterations in the immune-stained area % of Bcl-2 (Fig. 8E) and caspase-3 (Fig. 9E).

The hepatic and kidney tissue sections of CYP-treated rats exhibited weak immunostaining of Bcl-2 (Fig. 8C) and strong positive immunostaining of caspase-3 (Fig. 9C). Compared with the normal control values, this group displayed a significant reduction in the immune-stained area % of Bcl-2 (Fig. 8E) and a significant increase in the immune-stained area % of caspase-3 (Fig. 9E).

Compared with the CYP-treated group, the Ginseng+ CYP-treated rats’ hepatic and renal tissues displayed moderate positive Bcl-2 (Fig. 8D) and mild caspase-3 immunoreactivity (Fig. 9D) with a significant increase in immune-stained area % of Bcl-2 (Fig. 8E) and a significant reduction in immune-stained area % of caspase-3 (Fig. 9E).

### Molecular docking interactions

**Fig. 10:**
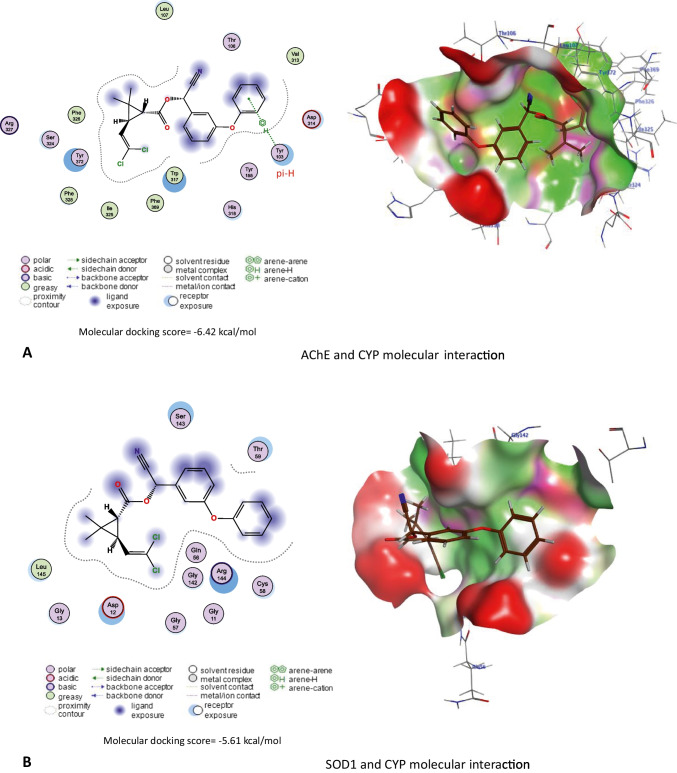

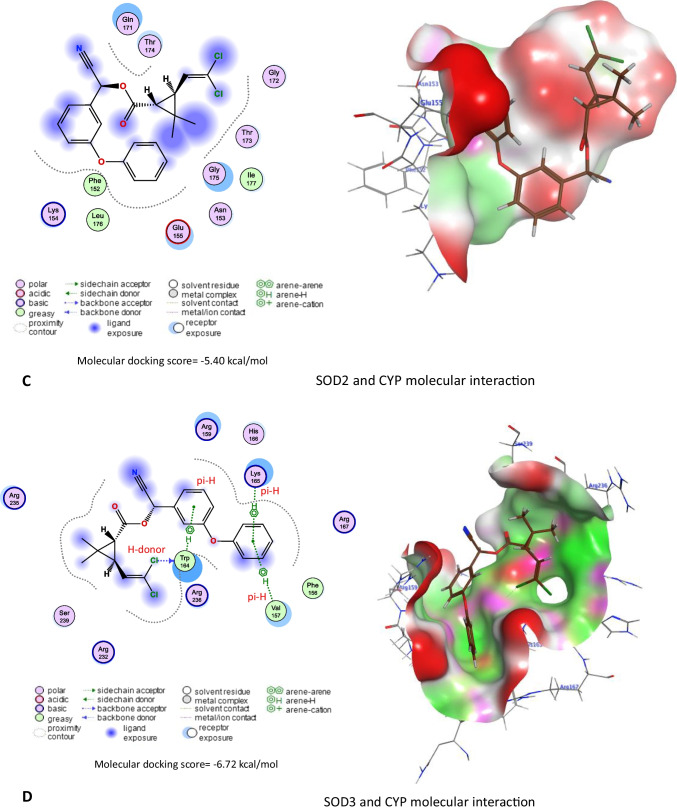

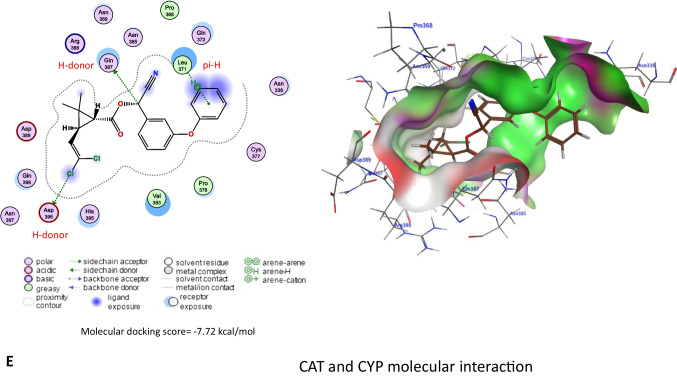
Molecular docking interaction of cypermethrin (CYP) with **A** acetylcholinesterase (AChE), **B** Cu/Zn-superoxide dismutase (SOD1), **C** Mn-superoxide dismutase (SOD2), **D** extracellular superoxide dismutase (SOD3), and **E** catalase (CAT) in rats

The molecular docking study indicated that CYP exhibited affinity to bind and inhibit AChE, SOD1, SOD2, SOD3, and CAT in rats with −6.42, −5.6, −5.40, −6.72, and −7.72 kcal/mol binding energy, respectively as stated in Fig. 10.

**Fig. 11:**
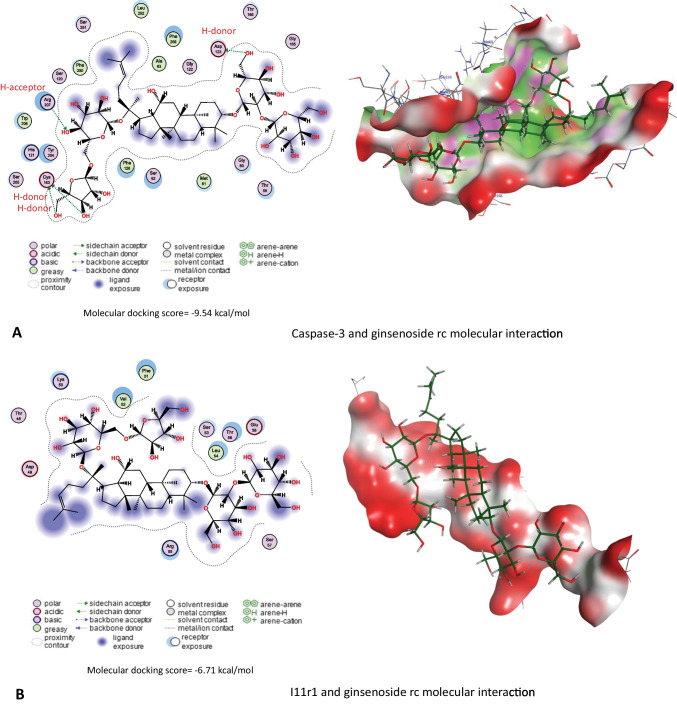

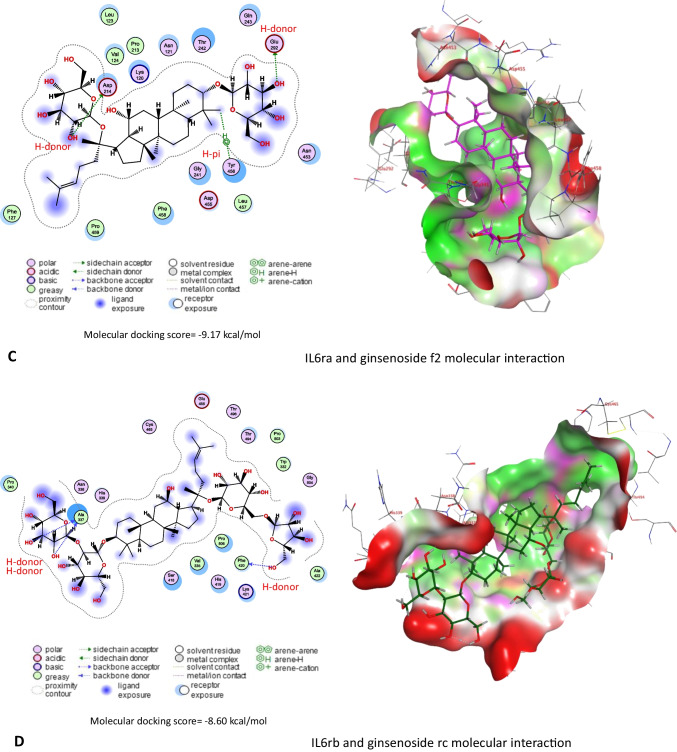

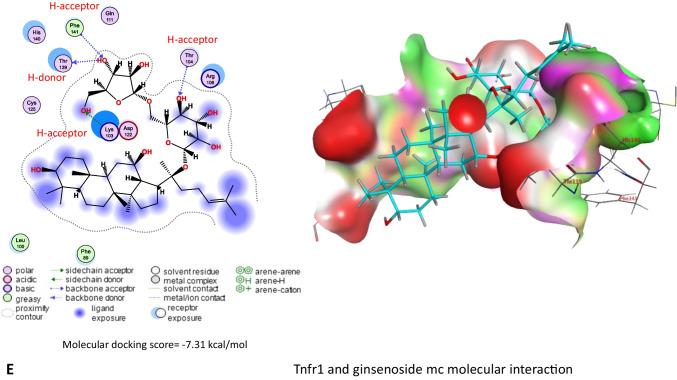
Molecular docking interaction of *Panax ginseng* active compounds. Molecular interaction of **A** ginsenoside rc with caspase-3, **B** ginsenoside rc with interleukin-1 alpha receptor-1 (Il1r1), **C** ginsenoside f2 with interleukin-6 receptor subunit alpha (Il6ra), **D** ginsenoside rc with interleukin-6 receptor subunit beta (Il6rb), and **E** ginsenoside mc with tumor necrosis factor receptor 1 (Tnfr1) in rats

On the other hand, results in Table 3 and Fig. 11A–E revealed that *Panax ginseng* active compounds interacted with the binding sites of caspase-3, Il1r1, Il6ra, Il6rb, and Tnfr1 in rats. Ginsenoside rc bound with caspase-3 (Fig. 11A), Il1r1 (Fig. 11B), and Il6rb (Fig. 11D) by −9.54, −6.71, and 8.60 kcal/mol, respectively. Furthermore, ginsenoside f2 (Fig. 11C) interacted with Il6ra (−9.17 kcal/mol), while ginsenoside mc (Fig. 11E) bound Tnfr1 by a binding energy of −7.31 kcal/mol.

**Table 3:** Molecular docking scores of *Panax ginseng* active compounds against caspase-3, interleukin-1 alpha receptor-1 (Il1r1), interleukin-6 receptor subunit alpha (Il6ra), interleukin-6 receptor subunit beta (Il6rb), and tumor necrosis factor receptor 1 (Tnfr1)

Lotus ID	Compounds	Molecular docking scores (kcal/mol)				
		Caspase-3	IL1r1	IL6ra	IL6rb	Tnfr1
LTS0028747	Cuparene	−4.33	−4.22	−5.32	−5.01	−4.49
LTS0076521	Ginsenoside rf	−8.02	−6.16	−8.38	−7.93	−6.74
LTS0085258	Andrographolide	−5.68	−4.56	−5.95	−5.77	−5.06
LTS0093069	Ginsenoside f2	−7.82	−6.67	−9.17	−7.93	−6.77
LTS0093105	Ginsenoside f1	−7.52	−6.23	−7.76	−7.17	−5.99
LTS0094839	Ginsenoside rg5	−8.91	−5.58	−8.30	−8.60	−6.57
LTS0110949	Ginsenoside rg2	−7.81	−5.87	−8.74	−7.45	−5.51
LTS0115294	(20r)-Ginsenoside rg3	−7.53	−6.00	−7.97	−8.50	−6.78
LTS0116351	Ginsenoside rc	−9.54	−6.71	−8.42	−8.39	−6.92
LTS0123697	β-Cubebene	−4.42	−4.26	−5.64	−4.87	−4.35
LTS0164245	Ginsenoside mx	−7.53	−5.91	−8.06	−7.54	−7.09
LTS0184823	Falcarinol	−5.87	−5.01	−6.05	−6.87	−5.92
LTS0187875	(20s)-Ginsenoside rh2	−7.41	−5.69	−7.32	−7.86	−5.68
LTS0206570	Ginsenoside mc	−7.98	−6.09	−8.27	−6.90	−7.31
LTS0225133	Ginsenoside rg1	−6.97	−6.19	−7.90	−7.76	−6.16

## Discussion

Although pesticides benefit crop production, they pollute the air, soil, water, and the global ecosystem, posing numerous health risks (Özkara et al. 2016). CYP is a pesticide that can be inhaled, ingested, and absorbed through the skin (Seven et al. 2022). Because of its hydrophobic nature, CYP generates ROS that initiate oxidative stress and apoptosis (Abdul-Hamid et al. 2017). CYP disrupted the oxidative/antioxidant status and induced an impairment of hepatic and renal histoarchitectures by damaging the cellular membranes and triggering DNA injury (Li et al. 2022). Many antioxidants are used to combat CYP’s harmful effects, and the current experiment aimed to investigate if * ginseng* could defend against CYP-induced hazards (Nauen et al. 2022). *Ginseng* is a potent antioxidant and effective in reducing tissue damage induced by free radical; also, * ginseng* has a protective effect against many toxicants in human and experimental animals and can increase body resistance to many harmful factors and can protect tissues from damage when an organism is in stress (Mostafa et al. 2021).

In the current work, CYP provoked a marked decline in body weight compared with control groups. This could be accredited to the impact of CYP on the gastrointestinal tract causing a reduced appetite, protein synthesis, and impaired absorption of nutrients from the gut (Ma et al. 2022). Additionally, the * ginseng* co-administration with CYP exhibited an improved body weight which might be attributed to the enhanced protein biosynthesis and feed intake (Nam et al. 2018).

The liver enzymes play a critical role in regulating physiological processes, for example, biosynthesis of macromolecules, cellular metabolism, and detoxification (Elblehi et al. 2019). In our study, the CYP administration caused significant upsurges in the serum ALT and AST activities and AChE activity compared to a control group. This may be due to the ability of CYP to induce oxidative stress and production of ROS, causing hepatocellular necrosis or membrane damage, leading to the liberation of these enzymes from hepatic cells to blood (Taha et al. 2021). Our results agree with previous findings of Abdou and Sayed (2019) who reported that CYP administration induced liver enzyme activities, through inflammatory cytokines, metabolic dysfunction, apoptosis, and gene expression modulation that contribute to CYP’s hepatorenal damage. CYP is expected to have two modes of action: it easily crosses and accumulates in biological membranes leading to stimulate the production of ROS and result in oxidative damage to essential cell components caused by oxygen-free radicals (Khanna et al. 2022).

Hypoproteinemia and hypoalbuminemia in intoxicated rats may have resulted from a decline in protein synthesis by hepatic cells reflecting hepatocellular necrosis. In agreement with Abdul-Hamid et al. (2017), our results also showed significant decreases in the serum TP and albumin. This may be explained by the ability of CYP to induce liver damage and decrease the synthesis, digestion, and absorption of protein because the liver is the main site for plasma protein synthesis (Ma et al. 2022). Data of the current study displayed a marked decline in TAG and a rise in T. cholesterol values. This might be because of the ability of the pesticide to block the bile duct and so reduce release of cholesterol in the intestine or due to the pancreatic dysfunction triggered by CYP, causing a lower lipid absorption (Taha et al. 2021). In the same context, Abdul-Hamid et al. (2017) described a marked enhancement in cholesterol and a decline in TAG values in rats orally administered with CYP for 28 days.

Concerning the renal function markers, our data showed a considerable rise in serum urea and creatinine concentrations in the CYP group. These data might reveal renal oxidative damage and dysfunction (Elblehi et al. 2019). It could also be because of a reduction in glomerular filtration (Khanna et al. 2022). The hepatorenal damage induced by CYP administration in the present study is associated with the generation of oxidative stress (Elblehi et al. 2015) that associated with significant decline in hepatorenal GSH level and antioxidant enzyme (SOD and CAT) activities related with remarkable rises in MDA level in CYP given rats. Similarly, CYP produces a lot of ROS in the liver (Khanna et al. 2022). CYP oxidation is mediated by microsomal cytochrome P450 (Bhutia et al. 2015; Ganguly et al. 2022). Also, CYP is detoxified by glutathione conjugation leading to a reduction in GSH levels and the concomitant rise in oxidized glutathione levels (Abdul-Hamid et al. 2017). Also, molecular docking assessment revealed that CYP exhibited harmful effect to antioxidant enzymes (SOD1, SOD2, SOD3, and CAT) in rats and humans, which might explain the oxidative damage associated with CYP-induced hepatorenal alterations associated with reduced antioxidant enzyme activities and monitored with increases in serum ALT, AST, urea, and creatinine along with significant decreased in serum TP, albumin, and globulin levels. This could be clarified by the capability of CYP to encourage liver damage and reduce protein production (Ma et al. 2022).

Furthermore, CYP induced nervous system function disruption by inhibiting AChE, an enzyme that breaks down the neurotransmitter acetylcholine (ACh) in neuromuscular junctions and cholinergic synapses within various tissues of ganglia (Li et al. 2022). This result was also confirmed with findings of the molecular docking of the current study. The molecular docking indicated that CYP exhibited affinity to bind and inhibit AChE. Reduction of AChE activity leads to the accumulation of Ach which has a damaging impact on the conduction of the nerve impulse through the cholinergic synapses (Hussien et al. 2013; Sharma et al. 2019).

Our data displayed that co-administration of * ginseng* to CYP-administered rats reduced hepatorenal damage. * ginseng* encouraged considerable declines in the serum ALT, AST activities, urea, creatinine, and cholesterol values with significant improvement in AChE, TP, albumin, and TAG concentrations relative to the CYP group. Additionally, the * ginseng* treatment alone presented a remarkable upsurge in TP levels compared with the control. Other similar results display that * ginseng* can defend the liver contrary to D-galactosamine/lipopolysaccharide (Nam et al. 2018), fipronil (Abd Eldaim et al. 2020), and cyhalothrin (Abdul-Hamid et al. 2020). Also, * ginseng* protects the kidney against gentamycin-induced renal damage (Raheem et al. 2017). Furthermore, * ginseng* improved the AChE levels in the CYP group, which indicated a reduced accumulation of Ach and enhanced nerve conduction through the neuromuscular junctions and cholinergic synapses (Ghamry et al. 2022).

The initial effect of CYP harmful effect is that it stimulates Kupffer cells in the liver, leading to the production of pro-inflammatory cytokines such as IL-1*β*, IL-6, and interferon gamma (Taha et al. 2021). Pro-inflammatory cytokines that promote inflammation elaborated in innate and acquired immunity, cell proliferation, tissue necrosis, and apoptosis (Hafez et al. 2020). In the present work, the CYP administration enhanced serum values of TNF-*α* significantly due to hepatic necrosis and mononuclear cell infiltration triggered by CYP (Abdul-Hamid et al. 2017), indicating inflammatory response. Conversely, co-treatment of * ginseng* to CYP-administered rats caused a considerable reduction in TNF-*α*, which might be attributed to the anti-inflammatory impact of ginseng (2020).

Likewise, CYP triggers mitochondrial dysfunction by altering the mitochondrial proteome, causing apoptosis, and inducing oxidative stress (Coughlan et al. 2015). The reported hepatorenal dysfunction was affirmed by the histopathological findings, which were established in the CYP-administered rats as degenerative changes, inflammation, proliferation of Kupffer cells, hepatocellular and renal necrosis, vascular congestion, hemorrhages, compressed and necrotic capillary tufts with a widening of Bowman’s space, and tubulo-interstitial nephritis and apoptosis. Consistently, these alterations may be due to the ability of CYP to initiate oxidative stress, mitochondrial dysfunction, ROS generation, and cytoskeleton changes resulting in cell death necrosis or apoptosis (Prabhudesai et al. 2010), whereas ROS affects various cellular components, including proteins, DNA, and RNA, disrupting vital cellular processes (Hafez et al. 2020). Concomitant oral administration of * ginseng* with CYP ameliorated CYP-induced hepatorenal damages. This may be due to the antioxidant properties of ginseng (Ghamry et al. 2022).

The primary markers of apoptosis are Bcl-2 family members, which include anti-apoptotic Bcl-2 and pro-apoptotic protein caspase-3 proteins. During apoptosis, the Bcl-2 expression was downregulated, while caspase-3 expression was upregulated (Deavall et al. 2012). Herein, the immunohistochemical staining of liver and kidney tissues revealed that CYP induced a significant decrease in the Bcl-2 and an increase in the caspase-3 immunohistochemical staining, which reflected the apoptotic effect of CYP. Nevertheless, concomitant oral administration of *P. ginseng* with CYP enhanced Bcl-2 and reduced caspase-3 immunohistochemical staining, implying anti-apoptotic activity of *P. ginseng* that conserved cellular integrity and mitigated CYP-induced hepatorenal damage (Abd Eldaim et al. 2020).

*Ginseng* anti-oxidative abilities have long been documented because of its property to improve the antioxidant enzyme gene expression that hunts ROS (Ghamry et al. 2022). *Ginseng* efficiently enhanced the activity of the antioxidant enzymes (SOD, CAT, and GPx), as well as reduced lipid peroxidation (Abdul-Hamid et al. 2020). *Ginseng* improves glomerular filtration and enhances the integrity of the glomerular basement membrane (Abd Eldaim et al. 2020). The hepatorenal protective impact of ginseng could be attributed to its pharmacological abilities, which contain antihyperlipidemic and anti-inflammatory properties (Kim et al. 2017). Also, these data could be established by the documented antioxidant impacts of co-administration with ginseng in the current experiment, revealing marked improvements in GSH levels and SOD and CAT activities associated with marked MDA level reductions compared with the CYP group. These data were approved by Raheem et al. (2017), Abd Eldaim et al. (2020), and Ghamry et al. (2022) in renal and hepatic tissues. On the other hand, *Panax ginseng* exhibited a high affinity toward Bcl-2, caspase, and inflammatory cytokine receptors that might alleviate the inflammatory and apoptotic processes associated with CYP exposure, which might explain the anti-apoptotic and anti-inflammatory impact of ginseng to combat the harmful effects of CYP (Ghamry et al. 2022).

## Conclusions

CYP is a pyrethroid associated with numerous health hazards. In the current study, CYP-induced oxidative stress damage in the liver and kidneys was monitored by increasing MDA levels and reducing GSH levels with a sharp decline in T-SOD and CAT activities. Consequently, the depletion of antioxidant potentials of liver and kidney tissues due to CYP led to inflammation and apoptosis initiation. On the contrary, ginseng successfully alleviated the oxidative damage and apoptosis induced by CYP, consequently improving the function and histoarchitectures of the liver and kidney; these results were affirmed with the molecular docking findings. In conclusion, we can determine that ginseng is considered a promising protective supplement against CYP-associated oxidative injuries. Therefore, we encourage researchers to examine the various protective impacts of ginseng in the suspected CYP-polluted districts for protection of exposed human and livestock.

### Supplementary materials

ESM 1 Supplementary File 1. Biological activities of ginsenoside rf (XLS 36 kb)

ESM 2 Supplementary File 2. Biological activities of andrographolide (XLS 180 kb)

ESM 3 Supplementary File 3. Biological activities of ginsenoside f2 (XLS 3 kb)

ESM 4 Supplementary File 4. Biological activities of ginsenoside f1 (XLS 17 kb)

ESM 5 Supplementary File 5. Biological activities of ginsenoside rg5 (XLS 8 kb)

ESM 6 Supplementary File 6. Biological activities of ginsenoside Rg2 (XLS 16 kb)

ESM 7 Supplementary File 7. Biological activities of (20r)-ginsenoside rg3 (XLS 59 kb)

ESM 8 Supplementary File 8. Biological activities of ginsenoside rc (XLS 508 bytes)

ESM 9 Supplementary File 9. Biological activities of falcarinol (XLS 11 kb)

ESM 10 Supplementary File 10. Biological activities of ginsenoside Rh2 (XLS 24 kb)

ESM 11 Supplementary File 11. Biological activities of ginsenoside Rg1 (XLS 65 kb)

ESM 12 Supplementary File 12. Clinical trials of *Panax ginseng*

## Data Availability

All data are represented in the manuscript and supplementary materials.
