# Supplementary material for: Panax ginseng ameliorates hepatorenal oxidative alterations induced by commercially used cypermethrin in male rats: experimental and molecular docking approaches
Source: Environ Sci Pollut Res Int. 2023 Sep 30;30(50):109702–23. doi: 10.1007/s11356-023-29935-2 (PMC10622388; doi:10.1007/s11356-023-29935-2)
Supplement: Supplementary file 12 — Clinical trials of Panax ginseng [file 11356_2023_29935_MOESM12_ESM.pdf]

ClinicalTrials.gov Search Results 11/22/2022

	NCT Number	Title	Other Names	Status	Conditions	Interventions	Characteristics	Population	Sponsor/ Collaborators	Funder Type	Dates	Locations
1	NCT03947554	Ginseng HRG80 in Stress and Fatigue<br><br>Study Documents:	Title Acronym:<br><br>Other Ids:<br>EP-1005	Completed	•Psychological Stress	•Dietary Supplement: HRG80 Panax ginseng<br>•Dietary Supplement: Panax ginseng<br>•Dietary Supplement: Placebo	Study Type:<br>Interventional<br><br>Phase:<br>•Phase 1<br>•Phase 2<br><br>Study Design:<br>•Allocation: Randomized<br>•Intervention Model: Crossover Assignment<br>•Masking: Double (Participant, Care Provider)<br>•Primary Purpose: Prevention<br><br>Outcome Measures:<br>Change in Perceived Stress Scale (PSS) from baseline to the last day of treatment	Enrollment:<br>50<br><br>Age:<br>18 Years to 65 Years (Adult, Older Adult)<br><br>Sex:<br>All	•EuroPharma, Inc.<br>•Botalys SA	•Industry<br>•Other	Study Start:<br>April 1, 2019<br><br>Primary Completion:<br>June 15, 2019<br><br>Study Completion:<br>July 1, 2019<br><br>First Posted:<br>May 13, 2019<br><br>Results First Posted:<br>No Results Posted<br><br>Last Update Posted:<br>July 5, 2019	•Sports Medicine and Anti-Doping Service Republican Centre, Yerevan, Armenia
2	NCT02211352	Effect of Korean Red Ginseng on Central Blood Pressure in Patient With Essential Hypertension<br><br>Study Documents:	Title Acronym:<br>KRGCBP<br><br>Other Ids:<br>GS302-100	Completed	•Hypertension	•Drug: Korean Red Ginseng Capsules<br>•Drug: Placebo	Study Type:<br>Interventional<br><br>Phase:<br>Phase 2<br><br>Study Design:<br>•Allocation: Randomized<br>•Intervention Model: Crossover Assignment<br>•Masking: Double (Participant, Investigator)<br>•Primary Purpose: Supportive Care<br><br>Outcome Measures:<br>•central blood pressure change after treatment of Korea Red Ginseng<br>•brachial systolic and diastolic blood pressure<br>•Augumentation index<br>•ESR(erythrocyte sedmentation rate)<br>•CRP(C-reactive protein)	Enrollment:<br>58<br><br>Age:<br>18 Years to 70 Years (Adult, Older Adult)<br><br>Sex:<br>All	•Daegu Catholic University Medical Center<br>•The Korean Society of Ginseng	•Other	Study Start:<br>May 2012<br><br>Primary Completion:<br>December 2013<br><br>Study Completion:<br>July 2014<br><br>First Posted:<br>August 7, 2014<br><br>Results First Posted:<br>No Results Posted<br><br>Last Update Posted:<br>August 7, 2014	•Daegu Catholic University Medical Center, Daegu, Korea, Republic of

	NCT Number	Title	Other Names	Status	Conditions	Interventions	Characteristics	Population	Sponsor/ Collaborators	Funder Type	Dates	Locations
3	NCT02999048	Clinical Study on the Treatment of Hypertensive Intracerebral Hemorrhage With Panax Notoginseng Saponin<br>Study Documents:	Title Acronym: CSTHIHPNS<br>Other Ids: 2012CC47	Completed	•Hematoma Absorption and Neurological Function Recovery	•Drug: Panax Notoginseng Saponins	Study Type: Interventional<br>Phase: Phase 4<br>Study Design:<br>•Allocation: Randomized<br>•Intervention Model: Parallel Assignment<br>•Masking: Double (Participant, Outcomes Assessor)<br>•Primary Purpose: Treatment<br>Outcome Measures:<br>•Hematoma volume<br>•National Institutes of Health Stroke Scale (NIHSS) scores<br>•Barthel index<br>•adverse events	Enrollment: 90<br>Age: 50 Years to 80 Years (Adult, Older Adult)<br>Sex: All	•The First People's Hospital of Jingzhou	•Other	Study Start: May 2014<br>Primary Completion: May 2016<br>Study Completion: May 2016<br>First Posted: December 21, 2016<br>Results First Posted: No Results Posted<br>Last Update Posted: December 21, 2016	
4	NCT03945123	Effects of Red Ginseng on Liver Function<br>Study Documents:	Title Acronym:<br>Other Ids: RIVER	Completed	•Liver Dysfunction	•Dietary Supplement: Red ginseng	Study Type: Interventional<br>Phase: Not Applicable<br>Study Design:<br>•Allocation: N/A<br>•Intervention Model: Single Group Assignment<br>•Masking: None (Open Label)<br>•Primary Purpose: Treatment<br>Outcome Measures:<br>Liver enzyme	Enrollment: 94<br>Age: 37 Years to 63 Years (Adult)<br>Sex: All	•Chuncheon Sacred Heart Hospital	•Other	Study Start: January 1, 2018<br>Primary Completion: December 31, 2018<br>Study Completion: December 31, 2018<br>First Posted: May 10, 2019<br>Results First Posted: No Results Posted<br>Last Update Posted: May 10, 2019	•ChuncheonSHH, Chuncheon, Kangwon, Korea, Republic of

	NCT Number	Title	Other Names	Status	Conditions	Interventions	Characteristics	Population	Sponsor/ Collaborators	Funder Type	Dates	Locations
5	NCT03775837	Effect of Panax Ginseng C.A. Mey Extract on Liver Function in Adults<br><br>Study Documents:	Title Acronym:<br><br>Other Ids: 02-2018-031	Completed	• Liver Diseases	• Dietary Supplement: Panax Ginseng C.A. Mey Extract group<br>• Dietary Supplement: Placebo group	Study Type: Interventional<br><br>Phase: Not Applicable<br><br>Study Design:<br>• Allocation: Randomized<br>• Intervention Model: Parallel Assignment<br>• Masking: Triple (Participant, Care Provider, Investigator)<br>• Primary Purpose: Treatment<br><br>Outcome Measures:<br>• Alanine aminotransferase<br>• Aspartate aminotransferase<br>• Gamma-glutamyl transferase	Enrollment: 60<br><br>Age: 19 Years to 75 Years (Adult, Older Adult)<br><br>Sex: All	• Pusan National University Yangsan Hospital	• Other	Study Start: December 1, 2018<br><br>Primary Completion: July 30, 2020<br><br>Study Completion: July 31, 2020<br><br>First Posted: December 14, 2018<br><br>Results First Posted: No Results Posted<br><br>Last Update Posted: September 16, 2020	• Integrated Research Institute for Natural Ingredients and Functional Foods, Yangsan, Korea, Republic of

	NCT Number	Title	Other Names	Status	Conditions	Interventions	Characteristics	Population	Sponsor/ Collaborators	Funder Type	Dates	Locations
6	NCT04069715	The Effect of Farlong® NotoGinseng™ (Ginseng Plus®) on Cholesterol and Blood Pressure<br><br>Study Documents:<br>• Study Protocol and Statistical Analysis Plan	Title Acronym:<br><br>Other Ids:<br>Farlong® NotoGinseng 16GCHY	Completed	• Hyperlipidemias<br>• Hypertension	• Dietary Supplement: Farlong NotoGinseng™ (Farlong Ginseng Plus® Panax Notoginseng extract)<br>• Other: Placebo	Study Type:<br>Interventional<br><br>Phase:<br>Phase 2<br><br>Study Design:<br>• Allocation: Randomized<br>• Intervention Model: Parallel Assignment<br>• Masking: Double (Participant, Investigator)<br>• Primary Purpose: Supportive Care<br><br>Outcome Measures:<br>• The Difference in Serum LDL-C From Baseline to Week 12 Between Farlong NotoGinseng™ (Farlong Ginseng Plus® Panax Notoginseng Extract) and Placebo After 12 Weeks of Supplementation.<br>• 1. The Difference in Serum LDL-C From Baseline to Week 8 Between Farlong Notoginseng and Placebo<br>• 2. The Difference in Blood Pressure From Baseline to Week 8 Between Farlong Notoginseng and Placebo<br>• 3. The Difference in Blood Pressure From Baseline to Week 12 Between Farlong Notoginseng and Placebo<br>• 4. The Difference in Triglycerides From Baseline to Week 8 Between Farlong Notoginseng and Placebo<br>• 5. The Difference in Triglycerides From Baseline to Week 12 Between Farlong Notoginseng and Placebo<br>• 6. The Difference in HDL-C From Baseline to Week 8 Between Farlong Notoginseng and Placebo<br>• 7. The Difference in HDL-C From Baseline to Week 12 Between Farlong Notoginseng and Placebo<br>• 8. The Difference in Total Cholesterol From Baseline to Week 8 Between Farlong Notoginseng and Placebo<br>• 9. The Difference in Total Cholesterol From Baseline	Enrollment:<br>95<br><br>Age:<br>18 Years to 75 Years (Adult, Older Adult)<br><br>Sex:<br>All	• LongStar HealthPro, Inc. DBA Farlong Pharmaceutical<br>• Yunnan PanLongYunHai Pharmaceuticals, Ltd.<br>• KGK Science Inc.	• Industry<br>• Other	Study Start:<br>July 20, 2016<br><br>Primary Completion:<br>June 1, 2019<br><br>Study Completion:<br>June 1, 2019<br><br>First Posted:<br>August 28, 2019<br><br>Results First Posted:<br>December 30, 2020<br><br>Last Update Posted:<br>May 17, 2021	

	NCT Number	Title	Other Names	Status	Conditions	Interventions	Characteristics	Population	Sponsor/ Collaborators	Funder Type	Dates	Locations
7	NCT03888196	Evaluation of the Efficacy of Panax Ginseng on Lipid Metabolism in Men and the Relationship With Sports Practice.<br><br>Study Documents:	Title Acronym: GinsengRun<br><br>Other Ids: Nutren-Disanta 0012018	Completed	• Sports Physical Therapy	• Dietary Supplement: Panax Ginseng Supplementation<br>• Dietary Supplement: Placebo Supplementation	Study Type: Interventional<br><br>Phase: Early Phase 1<br><br>Study Design:<br>• Allocation: Randomized<br>• Intervention Model: Parallel Assignment<br>• Masking: Double (Participant, Investigator)<br>• Primary Purpose: Treatment<br><br>Outcome Measures:<br>• Change from baseline plasmatic Total Lipids at 2 weeks<br>• Change from baseline plasmatic Triglycerides at 2 weeks<br>• Change from baseline Respiratory Exchange Rate at 2 weeks	Enrollment: 40<br><br>Age: 18 Years and older (Adult, Older Adult)<br><br>Sex: Male	• Universitat de Lleida	• Other	Study Start: September 1, 2016<br><br>Primary Completion: November 1, 2018<br><br>Study Completion: January 15, 2019<br><br>First Posted: March 25, 2019<br><br>Results First Posted: No Results Posted<br><br>Last Update Posted: May 2, 2019	

	NCT Number	Title	Other Names	Status	Conditions	Interventions	Characteristics	Population	Sponsor/ Collaborators	Funder Type	Dates	Locations
8	NCT01136928	Pharmacokinetic Interaction Study of Efavirenz and American Ginseng in Healthy Volunteers<br><br>Study Documents:	Title Acronym:<br><br>Other Ids:<br>• NA_00038067<br>• R01AT005526-01	Completed	• Healthy Volunteers	• Drug: American ginseng	Study Type:<br>Interventional<br><br>Phase:<br>Phase 1<br><br>Study Design:<br>• Allocation: N/A<br>• Intervention Model: Single Group Assignment<br>• Masking: None (Open Label)<br>• Primary Purpose: Other<br><br>Outcome Measures:<br>• To compare efavirenz AUC0-24 and Cmax when dosed alone to steady-state efavirenz AUC0-24 and Cmax given concurrently with American ginseng. Efavirenz AUC0-24 with and without American ginseng<br>• Efavirenz tmax with and without American ginseng<br>• Efavirenz Cmin with and without American ginseng<br>• Efavirenz Clearance with and without American ginseng<br>• Efavirenz T1/2 with and without American ginseng	Enrollment:<br>15<br><br>Age:<br>18 Years and older (Adult, Older Adult)<br><br>Sex:<br>Male	• Johns Hopkins University<br>• National Center for Complementary and Integrative Health (NCCIH)	• Other<br>• NIH	Study Start:<br>September 28, 2010<br><br>Primary Completion:<br>March 14, 2011<br><br>Study Completion:<br>March 14, 2011<br><br>First Posted:<br>June 4, 2010<br><br>Results First Posted:<br>No Results Posted<br><br>Last Update Posted:<br>June 15, 2018	• Johns Hopkins University, Baltimore, Maryland, United States

	NCT Number	Title	Other Names	Status	Conditions	Interventions	Characteristics	Population	Sponsor/ Collaborators	Funder Type	Dates	Locations
9	NCT01500096	American Ginseng to Improve HIV-Associated Fatigue: A Randomized, Placebo-Controlled, Parallel Design, Multiple-Dose Clinical Trial<br>Study Documents:	Title Acronym:<br>Other Ids: NA_00071671	Completed	•HIV/AIDS-associated Fatigue	•Drug: American ginseng<br>•Dietary Supplement: Placebo for American ginseng	Study Type: Interventional<br>Phase: Phase 2<br>Study Design:<br>•Allocation: Randomized<br>•Intervention Model: Parallel Assignment<br>•Masking: Quadruple (Participant, Care Provider, Investigator, Outcomes Assessor)<br>•Primary Purpose: Treatment<br>Outcome Measures:<br>•Change in Fatigue Severity Score (FSS)<br>•Change in the Brief Fatigue Inventory<br>•Change in Epworth Sleepiness Scale<br>•Change in Patient Health Questionnaire 9<br>•Change in Insomnia Severity Index<br>•Change in Medical Outcomes Study HIV Health Survey<br>•Changes in Clinical Global Impressions<br>•Inflammatory Markers<br>•Change in CD4 Cell Count<br>•Change in Plasma HIV RNA<br>•Change in PROMIS Fatigue<br>•Number of Participants With Adverse Events in the Ginseng and Placebo Arms	Enrollment: 96<br>Age: 18 Years and older (Adult, Older Adult)<br>Sex: All	•Johns Hopkins University<br>•National Center for Complementary and Integrative Health (NCCIH)	•Other<br>•NIH	Study Start: February 2013<br>Primary Completion: September 2016<br>Study Completion: September 2016<br>First Posted: December 26, 2011<br>Results First Posted: June 12, 2018<br>Last Update Posted: July 10, 2018	•The Johns Hopkins University, Baltimore, Maryland, United States

	NCT Number	Title	Other Names	Status	Conditions	Interventions	Characteristics	Population	Sponsor/ Collaborators	Funder Type	Dates	Locations
10	NCT00911768	Effect of Korean Red Ginseng (KRG) on Dry Mouth<br>Study Documents:	Title Acronym:<br>Other Ids: KHNMC-OH-IRB 2007-007	Completed	•Xerostomia	•Dietary Supplement: Korean Red Ginseng Powder Capsule<br>•Dietary Supplement: Corn-starch powder with ginseng flavor	Study Type: Interventional<br>Phase: Phase 4<br>Study Design: •Allocation: Randomized •Intervention Model: Parallel Assignment •Masking: Triple (Participant, Care Provider, Investigator) •Primary Purpose: Treatment<br>Outcome Measures: •Visual Analogue Scale of Subjective Dry Mouth •Stimulated Salivary Flow Rates •Unstimulated Salivary Flow Rates	Enrollment: 100<br>Age: 19 Years to 76 Years (Adult, Older Adult)<br>Sex: All	•The Korean Society of Ginseng	•Other	Study Start: September 2007<br>Primary Completion: May 2008<br>Study Completion: December 2008<br>First Posted: June 2, 2009<br>Results First Posted: June 2, 2009<br>Last Update Posted: August 6, 2009	•Kyung Hee East-West Neo Medical Center, Seoul, Korea, Republic of
11	NCT02386852	Ginseng and Ginkgo Biloba Effects on Cognition as Modulated by Cardiovascular Reactivity<br>Study Documents:	Title Acronym:<br>Other Ids: Sunway-979	Completed	•Blood Pressure<br>•Cognition	•Drug: Placebo<br>•Drug: Ginseng<br>•Drug: Ginkgo Biloba	Study Type: Interventional<br>Phase: Early Phase 1<br>Study Design: •Allocation: Randomized •Intervention Model: Crossover Assignment •Masking: Double (Participant, Investigator) •Primary Purpose: Basic Science<br>Outcome Measures: •Cognitive performance as modulated by either ginseng or ginkgo biloba •Cardiovascular reactivity as a result of ginseng or ginkgo biloba intake	Enrollment: 48<br>Age: 18 Years to 30 Years (Adult)<br>Sex: All	•Sunway University	•Other	Study Start: March 2014<br>Primary Completion: November 2014<br>Study Completion: November 2014<br>First Posted: March 12, 2015<br>Results First Posted: No Results Posted<br>Last Update Posted: March 13, 2015	•Department of Psychology, Sunway University, Bandar Sunway, Selangor, Malaysia

	NCT Number	Title	Other Names	Status	Conditions	Interventions	Characteristics	Population	Sponsor/ Collaborators	Funder Type	Dates	Locations
12	NCT02806349	Konjac-Glucomannan Fibre Blend and American Ginseng in Type 2 Diabetes<br><br>Study Documents:	Title Acronym:<br><br>Other Ids:<br>RB02-014C	Completed	• Diabetes Mellitus, Type 2	• Dietary Supplement: American Ginseng<br>• Dietary Supplement: Konjac-glucomannan fiber blend<br>• Dietary Supplement: Control	Study Type:<br>Interventional<br><br>Phase:<br>Phase 2<br><br>Study Design:<br>• Allocation: Randomized<br>• Intervention Model: Crossover Assignment<br>• Masking: None (Open Label)<br>• Primary Purpose: Treatment<br><br>Outcome Measures:<br>• HbA1c<br>• Total Cholesterol<br>• High sensitivity C reactive protein<br>• Apolipoprotein A and B<br>• Oxidized LDL<br>• Blood pressure<br>• LDL Cholesterol<br>• Triglycerides<br>• HDL-cholesterol<br>• fasting glucose<br>• fasting insulin	Enrollment:<br>39<br><br>Age:<br>40 Years to 75 Years (Adult, Older Adult)<br><br>Sex:<br>All	• Vladimir Vuksan<br>• Canadian Diabetes Association<br>• Unity Health Toronto	• Other	Study Start:<br>March 2002<br><br>Primary Completion:<br>November 2004<br><br>Study Completion:<br>March 2005<br><br>First Posted:<br>June 20, 2016<br><br>Results First Posted:<br>No Results Posted<br><br>Last Update Posted:<br>June 20, 2016	• Clinical Risk Factor and Modification Centre, Toronto, Ontario, Canada

	NCT Number	Title	Other Names	Status	Conditions	Interventions	Characteristics	Population	Sponsor/ Collaborators	Funder Type	Dates	Locations
13	NCT00401089	Efficacy Study of Panax Ginseng to Boost Antipsychotics Effects in Schizophrenia<br><br>Study Documents:	Title Acronym:<br><br>Other Ids:<br>R-02-285	Completed	•Schizophrenia<br>•Schizoaffective Disorder<br>•Tardive Dyskinesia<br>•Insulin Resistance<br>•Obesity	•Drug: Panax Ginseng	Study Type:<br>Interventional<br><br>Phase:<br>•Phase 1<br>•Phase 2<br><br>Study Design:<br>•Allocation: Randomized<br>•Intervention Model: Crossover Assignment<br>•Masking: Double (Participant, Investigator)<br>•Primary Purpose: Treatment<br><br>Outcome Measures:<br>•Neuro-Cognitive Screening Test<br>•PANSS Positive Negative Syndrome Scale<br>•SANS<br>•HAM-D Hamilton Depression Rating Scale<br>•BPRS Brief Psychiatric Rating Scale<br>•QLS Quality of Life Scale<br>•AIMS Abnormal Involuntary Movement Scale<br>•SAS Simpson Angus Scale for Extrapyramidal Symptoms<br>•Blood Chemistry Profile: CBC, kidney function,lipid profile, fasting glucose insulin<br>•BMI Body Mass index	Enrollment:<br>60<br><br>Age:<br>18 Years to 65 Years (Adult, Older Adult)<br><br>Sex:<br>All	•Lawson Health Research Institute<br>•Queen's University<br>•Northern Ontario School of Medicine<br>•Imperial College London	•Other	Study Start:<br>December 2002<br><br>Primary Completion:<br>December 2006<br><br>Study Completion:<br>October 2007<br><br>First Posted:<br>November 17, 2006<br><br>Results First Posted:<br>No Results Posted<br><br>Last Update Posted:<br>December 12, 2012	•Queen's University, Kingston, Ontario, Canada<br>•Regional Mental Health Care London, St. Thomas, Ontario, Canada<br>•Northern Ontario Medical School, Thunder Bay, Ontario, Canada<br>•Northwick Park Hospital, Harrow, Middlesex, United Kingdom

	NCT Number	Title	Other Names	Status	Conditions	Interventions	Characteristics	Population	Sponsor/ Collaborators	Funder Type	Dates	Locations
14	NCT00103012	Drug Interactions of Echinacea, Ginseng, and Ginkgo Biloba Taken With Lopinavir/Ritonavir in Healthy Volunteers<br>Study Documents:	Title Acronym:<br>Other Ids:<br>•050082<br>•05-CC-0082	Completed	•Healthy	•Drug: Gingko Biloba<br>•Drug: Echinacea purpurea<br>•Drug: Panax ginseng	Study Type: Interventional<br>Phase: Phase 4<br>Study Design:<br>•Allocation: Randomized<br>•Intervention Model: Crossover Assignment<br>•Masking: None (Open Label)<br>Outcome Measures:<br>Lopinavir Pharmacokinetics When Administered Alone and in Combination With Three Different Herbal Supplements: Ginkgo Biloba, Panax Ginsing, and Echinacea Purpurea.	Enrollment: 47<br>Age: 18 Years to 50 Years (Adult)<br>Sex: All	•National Institutes of Health Clinical Center (CC)	•NIH	Study Start: January 2005<br>Primary Completion: January 2011<br>Study Completion: June 2011<br>First Posted: February 7, 2005<br>Results First Posted: April 16, 2012<br>Last Update Posted: April 16, 2012	•National Institutes of Health Clinical Center, 9000 Rockville Pike, Bethesda, Maryland, United States
15	NCT01496248	Efficacy Study of Korean Red Ginseng to Treat Depression<br>Study Documents:	Title Acronym:<br>Other Ids: R1105721	Completed	•Major Depressive Disorder	•Dietary Supplement: Korean Red Ginseng	Study Type: Interventional<br>Phase: Not Applicable<br>Study Design:<br>•Allocation: N/A<br>•Intervention Model: Single Group Assignment<br>•Masking: None (Open Label)<br>•Primary Purpose: Treatment<br>Outcome Measures:<br>•Depression Residual Symptom Scale<br>•Visual Analogue Scale<br>•Montgomery Asberg Depression Rating Scale<br>•Clinical Global Index	Enrollment: 35<br>Age: 18 Years to 55 Years (Adult)<br>Sex: Female	•Korea University<br>•The Korean Society of Ginseng	•Other	Study Start: August 2011<br>Primary Completion: July 2012<br>Study Completion: December 2012<br>First Posted: December 21, 2011<br>Results First Posted: October 10, 2014<br>Last Update Posted: October 10, 2014	•Korea University Ansan Hospital, Ansan, Gyeonggi-do, Korea, Republic of

	NCT Number	Title	Other Names	Status	Conditions	Interventions	Characteristics	Population	Sponsor/ Collaborators	Funder Type	Dates	Locations
16	NCT01664156	Effect of Korean Red Ginseng on Women With Cold Hypersensitivity of Hands and Feet<br>Study Documents:	Title Acronym:<br>Other Ids: KHNMC-OH-IRB 2012-004	Completed	• Cold Hypersensitivity	• Drug: Korean red ginseng<br>• Drug: Placebo	Study Type: Interventional<br>Phase: Phase 4<br>Study Design:<br>• Allocation: Randomized<br>• Intervention Model: Parallel Assignment<br>• Masking: Double (Participant, Investigator)<br>• Primary Purpose: Treatment<br>Outcome Measures:<br>• The change of the infrared thermography of cold hypersensitivity on hands<br>• The change of the infrared thermography of cold hypersensitivity on feet<br>• The change of the Visual Analogue Scale of cold hypersensitivity on hands and feet<br>• The change of cold stress test<br>• The change of Distal-Dorsal Difference<br>• The change of Heart Rate Variability<br>• The change of 36-Item Short Form Health Survey	Enrollment: 80<br>Age: 16 Years to 60 Years (Child, Adult)<br>Sex: Female	• The Korean Society of Ginseng	• Other	Study Start: October 2012<br>Primary Completion: December 2013<br>Study Completion: March 2014<br>First Posted: August 14, 2012<br>Results First Posted: No Results Posted<br>Last Update Posted: March 14, 2014	• Kyung Hee University Hospital at Gangdong, Seoul, Gangdong-gu, Korea, Republic of

	NCT Number	Title	Other Names	Status	Conditions	Interventions	Characteristics	Population	Sponsor/ Collaborators	Funder Type	Dates	Locations
17	NCT04184388	Efficacy and Safety of Hydrolysed Red Ginseng Extract on Improvement of Cognitive Function<br><br>Study Documents:	Title Acronym:<br><br>Other Ids:<br>IH-CF-HRGE	Completed	• Cognitive Function	• Dietary Supplement: Hydrolysed Red Ginseng Extract<br>• Dietary Supplement: Placebo	Study Type:<br>Interventional<br><br>Phase:<br>Not Applicable<br><br>Study Design:<br>• Allocation: Randomized<br>• Intervention Model: Parallel Assignment<br>• Masking: Double (Participant, Investigator)<br>• Primary Purpose: Prevention<br><br>Outcome Measures:<br>• Visual learning test<br>• Visual working memory test<br>• MoCA-K; Korean Version of the Montreal Cognitive Assessment<br>• Auditory continuous performance test<br>• Verbal learning test<br>• PSS; Perceived stress scale<br>• BDI; Beck Depression Inventory<br>• BDNF; Brain-derived neurotrophic factor<br>• TAS(Total antioxidant status)	Enrollment:<br>100<br><br>Age:<br>60 Years and older (Adult, Older Adult)<br><br>Sex:<br>All	• Chonbuk National University Hospital	• Other	Study Start:<br>November 1, 2019<br><br>Primary Completion:<br>September 28, 2020<br><br>Study Completion:<br>November 30, 2020<br><br>First Posted:<br>December 3, 2019<br><br>Results First Posted:<br>No Results Posted<br><br>Last Update Posted:<br>January 27, 2021	• Department of Psychiatry, Chonbuk National University Hospital, Jeonju, Jeollabuk-do, Korea, Republic of

	NCT Number	Title	Other Names	Status	Conditions	Interventions	Characteristics	Population	Sponsor/ Collaborators	Funder Type	Dates	Locations
18	NCT02039635	Korean Red Ginseng in Treating Patients With Fatigue Caused by Chemotherapy for Colorectal Cancer<br><br>Study Documents:	Title Acronym: KRG<br><br>Other Ids: KGC-S-02	Completed	• Colorectal Cancer	• Dietary Supplement: Korean Red Ginseng<br>• Dietary Supplement: Placebo	Study Type: Interventional<br><br>Phase: Not Applicable<br><br>Study Design:<br>• Allocation: Randomized<br>• Intervention Model: Parallel Assignment<br>• Masking: Quadruple (Participant, Care Provider, Investigator, Outcomes Assessor)<br>• Primary Purpose: Supportive Care<br><br>Outcome Measures:<br>• Area Under Curve (AUC) of Brief Fatigue Index (BFI) on the intake trial drugs up to 16 weeks<br>• Area Under Curve (AUC) of Brief Fatigue Index (BFI) on the intake of trial drugs up to 8 weeks<br>• Change in Functional Assessment of Chronic Illness Therapy-Fatigue Trial Outcome Index (FACIT-F TOI) after 8 and 16 weeks of trial drug intake<br>• Change in Functional Assessment of Chronic Illness Therapy-Fatigue (FACIT-F) total score after 8 and 16 weeks of trial drug in take<br>• The percentage of subjects whom the Functional Assessment of Cancer Therapy-General (FACT-G) score decreased more than 20 scores after 8 and 16 weeks of trial drug intake<br>• Change in blood cytokine (IL-1, IL-6, TNF-alpha) level after 16 weeks of trial drug intake<br>• Change in blood cortisol level after 16 weeks of trial drug intake<br>• Perceived Stress Scale (PSS) change after 16 weeks of trial drug intake<br>• adverse events	Enrollment: 438<br><br>Age: 20 Years and older (Adult, Older Adult)<br><br>Sex: All	• Korea Ginseng Corporation	• Industry	Study Start: December 2013<br><br>Primary Completion: August 2016<br><br>Study Completion: March 2017<br><br>First Posted: January 17, 2014<br><br>Results First Posted: No Results Posted<br><br>Last Update Posted: August 11, 2017	• Korea Ginseng Corporation, Shinseongdong, Daejeon, Korea, Republic of

	NCT Number	Title	Other Names	Status	Conditions	Interventions	Characteristics	Population	Sponsor/ Collaborators	Funder Type	Dates	Locations
19	NCT00976274	Korean Red Ginseng and Metabolic Syndrome<br><br>Study Documents:	Title Acronym:<br><br>Other Ids: 3-2009-0015	Completed	• Metabolic Syndrome	• Dietary Supplement: Korean red ginseng<br>• Dietary Supplement: starch	Study Type: Interventional<br><br>Phase: Not Applicable<br><br>Study Design:<br>• Allocation: Randomized<br>• Intervention Model: Parallel Assignment<br>• Masking: Triple (Participant, Care Provider, Investigator)<br>• Primary Purpose: Treatment<br><br>Outcome Measures:<br>• Change in the Pre- and Post-treatment Systolic Blood Pressure<br>• Change in the Pre- and Post-treatment Oxidized Low-densty Lipoprotein(LDL)	Enrollment: 60<br><br>Age: 20 Years and older (Adult, Older Adult)<br><br>Sex: All	• The Korean Society of Ginseng	• Other	Study Start: August 2009<br><br>Primary Completion: January 2011<br><br>Study Completion: January 2011<br><br>First Posted: September 14, 2009<br><br>Results First Posted: February 24, 2012<br><br>Last Update Posted: February 27, 2012	• Yonsei Univeristy College of Medicine, Gangnam Severance Hospital, Seoul, Korea, Republic of

	NCT Number	Title	Other Names	Status	Conditions	Interventions	Characteristics	Population	Sponsor/ Collaborators	Funder Type	Dates	Locations
20	NCT00631852	A Phase II Biomarker Trial of Gelatin Encapsulated Extract of American Ginseng Root (LEAG) in Breast Cancer<br>Study Documents:<br>• Study Protocol and Statistical Analysis Plan	Title Acronym:<br>Other Ids: PER-SCCI 07-001.1	Completed	• Breast Cancer	• Drug: American Ginseng root	Study Type: Interventional<br>Phase: Phase 2<br>Study Design:<br>• Allocation: N/A<br>• Intervention Model: Single Group Assignment<br>• Masking: None (Open Label)<br>• Primary Purpose: Treatment<br>Outcome Measures:<br>• Adiponectin<br>• C Reactive Protein (CRP)<br>• Hepatocyte Growth Factor (HGF)<br>• Insulin Like Growth Factor 1 (IGF-1)<br>• Insulin Like Growth Factor 1 Receptor (IGF-1R)<br>• Interlueken-1- (IL-10)<br>• IL-12p40<br>• IL-1b<br>• IL-1ra<br>• IL-2<br>• and 9 more	Enrollment: 16<br>Age: 18 Years and older (Adult, Older Adult)<br>Sex: Female	• Southern Illinois University	• Other	Study Start: February 2008<br>Primary Completion: June 30, 2019<br>Study Completion: June 30, 2019<br>First Posted: March 10, 2008<br>Results First Posted: November 11, 2021<br>Last Update Posted: November 11, 2021	• Simmons Cancer Institute-SIU School of Medicine, Springfield, Illinois, United States
21	NCT01827696	Effect of American Ginseng on Exercise-induced Muscle Soreness<br>Study Documents:	Title Acronym:<br>Other Ids: 001	Completed	• Muscle Damage	• Dietary Supplement: Ginseng	Study Type: Interventional<br>Phase: Phase 1<br>Study Design:<br>• Allocation: Randomized<br>• Intervention Model: Parallel Assignment<br>• Masking: Double (Participant, Investigator)<br>• Primary Purpose: Prevention<br>Outcome Measures: muscle strength	Enrollment: 10<br>Age: 18 Years to 35 Years (Adult)<br>Sex: All	• Lawson Health Research Institute	• Other	Study Start: May 2013<br>Primary Completion: November 2013<br>Study Completion: December 2013<br>First Posted: April 9, 2013<br>Results First Posted: No Results Posted<br>Last Update Posted: March 3, 2016	• University of Western Ontario, London, Ontario, Canada

	NCT Number	Title	Other Names	Status	Conditions	Interventions	Characteristics	Population	Sponsor/ Collaborators	Funder Type	Dates	Locations
22	NCT03579095	Investigating the Acute and Chronic Effects of an American Ginseng Root Extract on Cognition and Mood<br>Study Documents:	Title Acronym:<br>Other Ids: RDG-004	Completed	•Cognitive Change<br>•Effects of Ginseng on Cognitive Function	•Dietary Supplement: Cereboost<br>•Dietary Supplement: Placebo	Study Type: Interventional<br>Phase: Not Applicable<br>Study Design:<br>•Allocation: Randomized<br>•Intervention Model: Parallel Assignment<br>•Masking: Quadruple (Participant, Care Provider, Investigator, Outcomes Assessor)<br>•Primary Purpose: Treatment<br>Outcome Measures:<br>•Rapid Visual Information Processing task<br>•Immediate word recall<br>•Corsi blocks task<br>•Modified Attention Network Task<br>•Task Switch Task<br>•Delayed word recall<br>•Mood	Enrollment: 60<br>Age: 18 Years to 30 Years (Adult)<br>Sex: All	•University of Reading<br>•Naturex	•Other	Study Start: May 4, 2018<br>Primary Completion: August 1, 2018<br>Study Completion: June 1, 2019<br>First Posted: July 6, 2018<br>Results First Posted: No Results Posted<br>Last Update Posted: July 1, 2019	•School of Psychology and Clinical Languages, Reading, United Kingdom
23	NCT00754832	American Ginseng Treatment for Multiple Sclerosis Related Fatigue<br>Study Documents:	Title Acronym:<br>Other Ids: 1357	Completed	•Multiple Sclerosis	•Drug: American ginseng extract HT-1001<br>•Drug: placebo	Study Type: Interventional<br>Phase: Phase 2<br>Study Design:<br>•Allocation: Randomized<br>•Intervention Model: Crossover Assignment<br>•Masking: Quadruple (Participant, Care Provider, Investigator, Outcomes Assessor)<br>•Primary Purpose: Treatment<br>Outcome Measures:<br>•Fatigue Severity Scale<br>•Modified Fatigue Impact Scale<br>•Realtime Digital Fatigue Score	Enrollment: 56<br>Age: 18 Years to 70 Years (Adult, Older Adult)<br>Sex: All	•Oregon Health and Science University<br>•National Multiple Sclerosis Society	•Other	Study Start: September 2005<br>Primary Completion: September 2008<br>Study Completion: March 2011<br>First Posted: September 18, 2008<br>Results First Posted: January 24, 2012<br>Last Update Posted: January 24, 2012	•Oregon Health & Science University, Portland, Oregon, United States

	NCT Number	Title	Other Names	Status	Conditions	Interventions	Characteristics	Population	Sponsor/ Collaborators	Funder Type	Dates	Locations
24	NCT02796664	Preventive Effects of Ginseng Against Atherosclerosis<br><br>Study Documents:<br>• Study Protocol and Statistical Analysis Plan	Title Acronym: PEGASUS<br><br>Other Ids: KGC2016-26	Completed	• Ischemic Stroke<br>• Atherosclerosis	• Dietary Supplement: Ginseng<br>• Dietary Supplement: Placebo	Study Type: Interventional<br><br>Phase: Not Applicable<br><br>Study Design:<br>• Allocation: Randomized<br>• Intervention Model: Parallel Assignment<br>• Masking: Quadruple (Participant, Care Provider, Investigator, Outcomes Assessor)<br>• Primary Purpose: Prevention<br><br>Outcome Measures:<br>• The Composite of Cerebral Ischemic Stroke and Transient Ischemic Attack<br>• Modified Rankin Scale<br>• The Changes in Volumetric Blood Flow (ml/Sec) in Intracranial Vessels.<br>• The Changes of White Matter Hyperintensities.<br>• Number of Participants With Changes of Parenchymal Ischemic Lesions	Enrollment: 58<br><br>Age: 20 Years to 80 Years (Adult, Older Adult)<br><br>Sex: All	• Dae Chul Suh<br>• Korea Ginseng Corporation<br>• Asan Medical Center	• Other<br>• Industry	Study Start: June 23, 2016<br><br>Primary Completion: July 4, 2018<br><br>Study Completion: July 4, 2018<br><br>First Posted: June 13, 2016<br><br>Results First Posted: August 24, 2021<br><br>Last Update Posted: August 24, 2021	• Asan Medical Center, Seoul, Korea, Republic of
25	NCT02331589	Anti-fatigue Effect of Korean Red Ginseng in Patients With Non-alcoholic Hepatitis<br><br>Study Documents:	Title Acronym:<br><br>Other Ids: AFEKRG	Completed	• Fatigue	• Drug: KRG (Korea Red ginseng)<br>• Drug: Placebo (for KRG)	Study Type: Interventional<br><br>Phase: Phase 4<br><br>Study Design:<br>• Allocation: Randomized<br>• Intervention Model: Parallel Assignment<br>• Masking: Single (Participant)<br>• Primary Purpose: Treatment<br><br>Outcome Measures:<br>• Liver Enzymes<br>• Fatigue as Measured by KRUPP's Fatigue Severity Scale<br>• Pro-inflammatory Cytokine<br>• Adiponectin	Enrollment: 75<br><br>Age: 18 Years to 80 Years (Adult, Older Adult)<br><br>Sex: All	• Chuncheon Sacred Heart Hospital	• Other	Study Start: August 2011<br><br>Primary Completion: April 2012<br><br>Study Completion: April 2012<br><br>First Posted: January 6, 2015<br><br>Results First Posted: February 10, 2015<br><br>Last Update Posted: February 10, 2015	

	NCT Number	Title	Other Names	Status	Conditions	Interventions	Characteristics	Population	Sponsor/ Collaborators	Funder Type	Dates	Locations
26	NCT03407716	Ginseng in Decreasing Cancer-Related Fatigue After Treatment in Cancer Survivors<br><br>Study Documents:	Title Acronym:<br><br>Other Ids:<br>•MC16C2<br>•NCI-2017-02494<br>•P30CA015083	Completed	•Cancer Survivor<br>•Stage I Breast Cancer AJCC v7<br>•Stage I Colon Cancer AJCC v6 and v7<br>•Stage IA Breast Cancer AJCC v7<br>•Stage IB Breast Cancer AJCC v7<br>•Stage II Breast Cancer AJCC v6 and v7<br>•Stage II Colon Cancer AJCC v7<br>•Stage IIA Breast Cancer AJCC v6 and v7<br>•Stage IIA Colon Cancer AJCC v7<br>•Stage IIB Breast Cancer AJCC v6 and v7<br>•and 10 more	•Drug: American Ginseng<br>•Other: Laboratory Biomarker Analysis<br>•Other: Placebo<br>•Other: Questionnaire Administration	Study Type:<br>Interventional<br><br>Phase:<br>Early Phase 1<br><br>Study Design:<br>•Allocation: Randomized<br>•Intervention Model: Crossover Assignment<br>•Masking: Double (Participant, Investigator)<br>•Primary Purpose: Supportive Care<br><br>Outcome Measures:<br>•Change in general subscale of the Multidimensional Fatigue Symptom Inventory- Short Form (MFSI-SF)<br>•Change in MFSI-SF emotional subscale<br>•Change in MFSI-SF general subscale<br>•Change in MFSI-SF mental subscale<br>•Change in MFSI-SF physical subscale<br>•Change in MFSI-SF vigor subscale<br>•Change in the single item numeric analogue fatigue question<br>•Fatigue as measured by the National Institutes of Health (NIH) Patient-Reported Outcomes Measurement Information System (PROMIS) score<br>•Fatigue as measured by the NIH PROMIS score<br>•Incidence of adverse events as reported by the patient in a Ginseng Symptom Experience Diary<br>•Perceived treatment efficacy as measured by the Subject Global Impression of Change	Enrollment:<br>2<br><br>Age:<br>18 Years and older (Adult, Older Adult)<br><br>Sex:<br>All	•Mayo Clinic<br>•National Cancer Institute (NCI)	•Other<br>•NIH	Study Start:<br>March 1, 2019<br><br>Primary Completion:<br>May 13, 2020<br><br>Study Completion:<br>February 22, 2021<br><br>First Posted:<br>January 23, 2018<br><br>Results First Posted:<br>No Results Posted<br><br>Last Update Posted:<br>March 24, 2022	•Mayo Clinic in Arizona, Scottsdale, Arizona, United States

	NCT Number	Title	Other Names	Status	Conditions	Interventions	Characteristics	Population	Sponsor/ Collaborators	Funder Type	Dates	Locations
27	NCT01616134	Effect of Korean Red Ginseng on Insulin Sensitivity in Non Diabetic Overweight Korean Adults<br>Study Documents:	Title Acronym: KRGIS<br>Other Ids: KRG-0912	Completed	•Insulin Resistance<br>•Obesity	•Other: Korea red ginseng<br>•Other: Placebo	Study Type: Interventional<br>Phase: Not Applicable<br>Study Design:<br>•Allocation: Randomized<br>•Intervention Model: Factorial Assignment<br>•Masking: Triple (Participant, Care Provider, Investigator)<br>•Primary Purpose: Prevention<br>Outcome Measures:<br>insulin sensitivity (insulin, HOMA-IR, QUICKI)	Enrollment: 80<br>Age: 20 Years to 60 Years (Adult)<br>Sex: All	•The Korean Society of Ginseng	•Other	Study Start: August 2010<br>Primary Completion: April 2011<br>Study Completion: April 2011<br>First Posted: June 11, 2012<br>Results First Posted: No Results Posted<br>Last Update Posted: March 3, 2017	•Pusan National University Yangsan Hospital, Yangsan, Gyeongsangnam-do, Korea, Republic of
28	NCT00527969	Cognitive, Emotional, Physical and Psychosocial Effects of Panax Quinquefolius L<br>Study Documents:	Title Acronym: REMEMBER-fX<br>Other Ids: HT1001-2006-2	Completed	•Memory<br>•Learning<br>•Attention<br>•Cognition<br>•Well-Being	•Drug: HT1001 extract of Panax quinquefolius L	Study Type: Interventional<br>Phase: Phase 2<br>Study Design:<br>•Allocation: Randomized<br>•Intervention Model: Crossover Assignment<br>•Masking: Double<br>•Primary Purpose: Treatment<br>Outcome Measures:<br>•Use of HT1001 will improve objective measures of psychomotor speed, sustained attention, working memory, declarative memory, and or executive skills.<br>•Use of HT1001 will be associated with no cognitive or physical adverse effects.	Enrollment: 72<br>Age: 35 Years to 75 Years (Adult, Older Adult)<br>Sex: All	•CV Technologies<br>•Afexa Life Sciences Inc	•Industry	Study Start: July 2007<br>Primary Completion:<br>Study Completion:<br>First Posted: September 11, 2007<br>Results First Posted: No Results Posted<br>Last Update Posted: March 12, 2008	•PNL, Edmonton, Alberta, Canada

	NCT Number	Title	Other Names	Status	Conditions	Interventions	Characteristics	Population	Sponsor/ Collaborators	Funder Type	Dates	Locations
29	NCT00391833	Effect of Panax Ginseng on the Cognitive Performance in Alzheimer's Disease<br><br>Study Documents:	Title Acronym:<br><br>Other Ids: ginseng-AD	Completed	•Alzheimer's Disease<br>•Memory Decline	•Drug: Panax Ginseng	Study Type: Interventional<br><br>Phase:<br>•Phase 1<br>•Phase 2<br><br>Study Design:<br>•Allocation: Non-Randomized<br>•Intervention Model: Single Group Assignment<br>•Masking: None (Open Label)<br>•Primary Purpose: Treatment<br><br>Outcome Measures:<br>•Cognitive performances monitored by MMSE and Alzheimer's disease assessment scales.<br>•Biomarkers including hematopoietic progenitor cell count.	Enrollment:<br><br>Age: 40 Years to 83 Years (Adult, Older Adult)<br><br>Sex: Female	•Seoul National University Hospital	•Other	Study Start: April 2004<br><br>Primary Completion:<br><br>Study Completion: October 2005<br><br>First Posted: October 24, 2006<br><br>Results First Posted: No Results Posted<br><br>Last Update Posted: October 24, 2006	
30	NCT01699074	Acute Dose Response of Korean White Ginseng in Metabolic Syndrome or Type 2 Diabetes<br><br>Study Documents:	Title Acronym: KWG<br><br>Other Ids: 182629	Completed	•Type 2 Diabetes<br>•Metabolic Syndrome	•Dietary Supplement: 1 gram of White Korean Ginseng<br>•Dietary Supplement: 3 grams of White Korean Ginseng<br>•Dietary Supplement: 6 grams of White Korean Ginseng<br>•Dietary Supplement: 3 grams of Wheat Bran Control<br>•Dietary Supplement: 500mg of Korean Red Ginseng	Study Type: Interventional<br><br>Phase: Phase 1<br><br>Study Design:<br>•Allocation: Randomized<br>•Intervention Model: Crossover Assignment<br>•Masking: Double (Participant, Investigator)<br>•Primary Purpose: Treatment<br><br>Outcome Measures: Effect of KWG on vascular and glycemic measures	Enrollment: 30<br><br>Age: 18 Years to 75 Years (Adult, Older Adult)<br><br>Sex: All	•Unity Health Toronto	•Other	Study Start: May 2013<br><br>Primary Completion: September 2013<br><br>Study Completion: January 2014<br><br>First Posted: October 3, 2012<br><br>Results First Posted: No Results Posted<br><br>Last Update Posted: April 15, 2014	•: Clinical Nutrition and Risk Factor Modification Centre, St. Michael's Hospital, Toronto, Ontario, Canada

	NCT Number	Title	Other Names	Status	Conditions	Interventions	Characteristics	Population	Sponsor/ Collaborators	Funder Type	Dates	Locations
31	NCT04167449	Effects of Korean Red Ginseng Extract on Electrical Brain Activity in Elderly Subjects<br>Study Documents:	Title Acronym:<br>Other Ids: EP-1006	Completed	• Brain Waves	• Dietary Supplement: HRG80 Hydroponic Red Ginseng<br>• Dietary Supplement: Conventional White Ginseng<br>• Dietary Supplement: Placebo	Study Type: Interventional<br>Phase: Phase 4<br>Study Design:<br>• Allocation: Randomized<br>• Intervention Model: Crossover Assignment<br>• Masking: Double (Participant, Care Provider)<br>• Primary Purpose: Treatment<br>Outcome Measures: Change in baseline electrical activity of the brain as measured by Quantitative Electroencephalogram for the hydroponic Korean red ginseng preparation versus conventional ginseng and placebo	Enrollment: 30<br>Age: 60 Years to 75 Years (Adult, Older Adult)<br>Sex: All	• EuroPharma, Inc.	• Industry	Study Start: November 1, 2019<br>Primary Completion: March 30, 2020<br>Study Completion: May 1, 2020<br>First Posted: November 18, 2019<br>Results First Posted: No Results Posted<br>Last Update Posted: February 16, 2021	• Clinical Labors of NeuroCode AG, Wetzlar, Germany

	NCT Number	Title	Other Names	Status	Conditions	Interventions	Characteristics	Population	Sponsor/ Collaborators	Funder Type	Dates	Locations
32	NCT01578837	Combined Rg3-enriched Korean Red Ginseng and American Ginseng in the Management of Hypertension in Type 2 Diabetes<br><br>Study Documents:	Title Acronym: R-KAT<br><br>Other Ids: OG-2-09-2920-VV	Completed	• Diabetes Mellitus Type 2<br>• Hypertension	• Dietary Supplement: Ginseng<br>• Dietary Supplement: Wheat Bran	Study Type: Interventional<br><br>Phase:<br>• Phase 1<br>• Phase 2<br><br>Study Design:<br>• Allocation: Randomized<br>• Intervention Model: Parallel Assignment<br>• Masking: Double (Participant, Investigator)<br>• Primary Purpose: Treatment<br><br>Outcome Measures:<br>• Change in Mean 24 hour Systolic Ambulatory Blood Pressure at 12 weeks<br>• Change in Mean 24 hour Diastolic, daytime and nighttime Ambulatory Blood pressure at 12 Weeks<br>• Change in Pulse Pressure<br>• Change in Central Augmentation Index and pulse wave analysis<br>• Change in Pulse Wave Velocity at 12 Weeks<br>• Change in low-grade body inflammation (hs-CRP)<br>• Change in HbA1c<br>• Change in Fasting Insulin<br>• Change in Fasting Glucose<br>• Change in Calculated HOMA-Insulin Sensitivity<br>• Change in RHI at 12 weeks<br>• Change in lipids at 12 weeks	Enrollment: 90<br><br>Age: 40 Years to 75 Years (Adult, Older Adult)<br><br>Sex: All	• Unity Health Toronto<br>• Canadian Diabetes Association	• Other	Study Start: April 2014<br><br>Primary Completion: September 2017<br><br>Study Completion: September 2017<br><br>First Posted: April 17, 2012<br><br>Results First Posted: No Results Posted<br><br>Last Update Posted: November 21, 2018	• Clinical Nutrition and Risk Factor Modification Centre, St. Michael's Hospital, Toronto, Ontario, Canada<br>• Clinical Centre Vuk Vrhovac, Zagreb, Croatia

	NCT Number	Title	Other Names	Status	Conditions	Interventions	Characteristics	Population	Sponsor/ Collaborators	Funder Type	Dates	Locations
33	NCT03828188	Efficacy and Safety of Red Ginseng Concentrated Powder on Improvement of Blood Triglyceride Level<br><br>Study Documents:	Title Acronym:<br><br>Other Ids:<br>JAR-FHL-RG2	Completed	•Hyperlipidemias	•Dietary Supplement: Red Ginseng Concentrated Powder<br>•Dietary Supplement: Placebo	Study Type:<br>Interventional<br><br>Phase:<br>Not Applicable<br><br>Study Design:<br>•Allocation: Randomized<br>•Intervention Model: Crossover Assignment<br>•Masking: Double (Participant, Investigator)<br>•Primary Purpose: Prevention<br><br>Outcome Measures:<br>•Changes of Fasting triglyceride<br>•Changes of Lipid metabolism indices<br>•Changes of Arteriosclerosis indices<br>•Changes of Carnitine (Serum)<br>•Changes of lipoprotein lipase	Enrollment:<br>50<br><br>Age:<br>19 Years to 70 Years (Adult, Older Adult)<br><br>Sex:<br>All	•Chonbuk National University Hospital	•Other	Study Start:<br>March 1, 2018<br><br>Primary Completion:<br>May 11, 2018<br><br>Study Completion:<br>May 8, 2019<br><br>First Posted:<br>February 4, 2019<br><br>Results First Posted:<br>No Results Posted<br><br>Last Update Posted:<br>May 8, 2020	•Clinical Trial Center for Functional Foods Chonbuk National University Hospital, Jeonju, Jeollabuk-do, Korea, Republic of

	NCT Number	Title	Other Names	Status	Conditions	Interventions	Characteristics	Population	Sponsor/ Collaborators	Funder Type	Dates	Locations
34	NCT02428998	Safety for 24 Weeks Intake of Korean Red Ginseng in Adults<br><br>Study Documents:	Title Acronym:<br><br>Other Ids:<br>KGC-S-01	Completed	•Healthy<br>•Diabetes<br>•Hypertension<br>•Hyperlipidemia	•Dietary Supplement: Korean Red Ginseng<br>•Dietary Supplement: Placebo	Study Type:<br>Interventional<br><br>Phase:<br>Not Applicable<br><br>Study Design:<br>•Allocation: Randomized<br>•Intervention Model: Parallel Assignment<br>•Masking: Quadruple (Participant, Care Provider, Investigator, Outcomes Assessor)<br>•Primary Purpose: Supportive Care<br><br>Outcome Measures:<br>•All adverse events occurring up to 24 weeks time after taking the Investigational product<br>•Adverse events that occurred up to 24 weeks to collect all focus point after taking the Investigational product<br>•Cardiovascular adverse events that occurred after taking the Investigational product<br>•Gastrointestinal adverse events that occurred after ingestion of Investigational product<br>•Neuropsychiatric adverse events that occurred after ingestion of Investigational product<br>•Grade 3 adverse events that occurred after ingestion of Investigational product<br>•Baseline by 24 weeks after the change in quality of life measures ingestion of Investigational product	Enrollment:<br>1000<br><br>Age:<br>19 Years and older (Adult, Older Adult)<br><br>Sex:<br>All	•Korea Ginseng Corporation	•Industry	Study Start:<br>September 2014<br><br>Primary Completion:<br>August 2016<br><br>Study Completion:<br>April 2017<br><br>First Posted:<br>April 29, 2015<br><br>Results First Posted:<br>No Results Posted<br><br>Last Update Posted:<br>August 11, 2017	•Catholic Universtiy of Korea. Seoul St Mary's Hospital, Seoul, Korea, Republic of

	NCT Number	Title	Other Names	Status	Conditions	Interventions	Characteristics	Population	Sponsor/ Collaborators	Funder Type	Dates	Locations
35	NCT01712373	Ginseng in Treatment of Fatigue in Multiple Sclerosis<br>Study Documents:	Title Acronym:<br>Other Ids: ASD-1270	Completed	•Fatigue	•Drug: Ginseng<br>•Drug: Placebo	Study Type: Interventional<br>Phase: Phase 2<br>Study Design:<br>•Allocation: Randomized<br>•Intervention Model: Parallel Assignment<br>•Masking: Single (Outcomes Assessor)<br>•Primary Purpose: Treatment<br>Outcome Measures:<br>•Fatigue<br>•Quality Of Life	Enrollment: 60<br>Age: 18 Years to 50 Years (Adult)<br>Sex: All	•Isfahan University of Medical Sciences	•Other	Study Start: December 2010<br>Primary Completion: April 2011<br>Study Completion: April 2011<br>First Posted: October 23, 2012<br>Results First Posted: No Results Posted<br>Last Update Posted: October 23, 2012	•Al-zahra university hospital, Isfahan, Iran, Islamic Republic of

	NCT Number	Title	Other Names	Status	Conditions	Interventions	Characteristics	Population	Sponsor/ Collaborators	Funder Type	Dates	Locations
36	NCT02763280	Efficacy and Safety of Ginseng Extract on Improvement of Bone Metabolism in Menopausal Women<br><br>Study Documents:	Title Acronym:<br><br>Other Ids:<br>RDA-BM-GE	Completed	•Osteopenia	•Dietary Supplement: Ginseng extract 1g<br>•Dietary Supplement: Ginseng extract 3g<br>•Dietary Supplement: Placebo	Study Type:<br>Interventional<br><br>Phase:<br>Not Applicable<br><br>Study Design:<br>•Allocation: Randomized<br>•Intervention Model: Parallel Assignment<br>•Masking: Double (Participant, Investigator)<br>•Primary Purpose: Prevention<br><br>Outcome Measures:<br>•Changes in Serum Osteocalcin<br>•Changes in Urinary Deoxypyridinoline<br>•Changes in DPD/OC ratio<br>•Changes in Serum CTX(Cross-linked C-telopeptide of type-1 collagen)<br>•Changes in Serum NTX(Cross-linked N-telopeptide of type-1 collagen)<br>•Changes in Serum Ca, Phosphorus<br>•Changes in Serum BSALP(bone specific-alkaline phosphatase)<br>•Changes in Serum P1NP(procollagen type 1 N-terminal propeptide)<br>•Changes in WOMAC index(Western ontario and mcmaster universities arthritis)	Enrollment:<br>90<br><br>Age:<br>40 Years and older (Adult, Older Adult)<br><br>Sex:<br>Female	•Chonbuk National University Hospital	•Other	Study Start:<br>June 2015<br><br>Primary Completion:<br>July 2015<br><br>Study Completion:<br>September 2016<br><br>First Posted:<br>May 5, 2016<br><br>Results First Posted:<br>No Results Posted<br><br>Last Update Posted:<br>February 5, 2018	•Clinical Trial Center for Functional Foods; Chonbuk National University Hospital, Jeonju, Jeollabuk-do, Korea, Republic of

	NCT Number	Title	Other Names	Status	Conditions	Interventions	Characteristics	Population	Sponsor/ Collaborators	Funder Type	Dates	Locations
37	NCT00781534	A Clinical Trial of Ginseng in Diabetes<br><br>Study Documents:	Title Acronym:<br><br>Other Ids: 03-0824	Completed	•Diabetes	•Drug: Ginseng<br>•Drug: ginsenoside RE<br>•Dietary Supplement: Placebo (sugar pill)	Study Type: Interventional<br><br>Phase: Early Phase 1<br><br>Study Design:<br>•Allocation: Randomized<br>•Intervention Model: Parallel Assignment<br>•Masking: Quadruple (Participant, Care Provider, Investigator, Outcomes Assessor)<br>•Primary Purpose: Basic Science<br><br>Outcome Measures:	Enrollment: 19<br><br>Age: 18 Years to 65 Years (Adult, Older Adult)<br><br>Sex: All	•Washington University School of Medicine	•Other	Study Start: September 2003<br><br>Primary Completion: December 2004<br><br>Study Completion: September 2008<br><br>First Posted: October 29, 2008<br><br>Results First Posted: No Results Posted<br><br>Last Update Posted: September 9, 2019	•Washington University School of Medicine, Saint Louis, Missouri, United States
38	NCT02204826	Effects of Korean Red Ginseng on Semen Parameters in Male Infertility Patients: a Randomized, Placebo-controlled, Double-blind Clinical Study<br><br>Study Documents:	Title Acronym:<br><br>Other Ids: KRG-infertility	Completed	•Male Infertility	•Drug: Korean Red Ginseng (KRG)<br>•Procedure: Varicocelectomy	Study Type: Interventional<br><br>Phase: Phase 4<br><br>Study Design:<br>•Allocation: Randomized<br>•Intervention Model: Parallel Assignment<br>•Masking: Double (Participant, Investigator)<br>•Primary Purpose: Treatment<br><br>Outcome Measures:<br>•Semen parmaters change from baseline to post-treatment<br>•Changes in hormonal parameters after treatment	Enrollment: 80<br><br>Age: 25 Years to 45 Years (Adult)<br><br>Sex: Male	•Pusan National University Hospital	•Other	Study Start: April 2011<br><br>Primary Completion: February 2012<br><br>Study Completion: December 2013<br><br>First Posted: July 30, 2014<br><br>Results First Posted: No Results Posted<br><br>Last Update Posted: August 29, 2018	•Department of Urology, Pusan National University Hospital, Busan, Korea, Republic of

	NCT Number	Title	Other Names	Status	Conditions	Interventions	Characteristics	Population	Sponsor/ Collaborators	Funder Type	Dates	Locations
39	NCT02056743	Clinical Trials on Evaluate the Red Ginseng and Fermented-Red Ginseng Affect to Drug Metabolizing Enzyme and Transporter in Healthy Volunteers<br><br>Study Documents:	Title Acronym:<br><br>Other Ids: CUH_2012_RG	Completed	•Healthy	•Drug: CYP cocktail<br>•Drug: Fexofenadine 30mg<br>•Dietary Supplement: Red ginseng<br>•Dietary Supplement: Fermented-red ginseng	Study Type: Interventional<br><br>Phase: Phase 1<br><br>Study Design:<br>•Allocation: Randomized<br>•Intervention Model: Parallel Assignment<br>•Masking: None (Open Label)<br><br>Outcome Measures:<br>•Maximum plasma concentration (Cmax)<br>•Area under the plasma concentration curve (AUClast)<br>•Area under the plasma concentration curve (AUCinf)<br>•First time to reach Cmax (Tmax)<br>•Terminal half-life (t1/2)<br>•Apparent Total Body Clearance (CL/F)<br>•Apparent Volume of Distribution (Vd/F)	Enrollment: 30<br><br>Age: 20 Years to 55 Years (Adult)<br><br>Sex: Male	•Chonbuk National University Hospital	•Other	Study Start: September 2013<br><br>Primary Completion: October 2013<br><br>Study Completion:<br><br>First Posted: February 6, 2014<br><br>Results First Posted: No Results Posted<br><br>Last Update Posted: February 6, 2014	•Clinical Trial Center of Chonbuk National University Hospital, Jeonju, Jeollabuk-do, Korea, Republic of

	NCT Number	Title	Other Names	Status	Conditions	Interventions	Characteristics	Population	Sponsor/ Collaborators	Funder Type	Dates	Locations
40	NCT03260543	Efficacy and Safety of Fermented Ginseng Powder on Liver Function<br><br>Study Documents:	Title Acronym:<br><br>Other Ids:<br>GB-LFE-GP	Completed	• Fatty Liver, Nonalcoholic	• Dietary Supplement: fermented ginseng powder<br>• Dietary Supplement: Placebo	Study Type:<br>Interventional<br><br>Phase:<br>Not Applicable<br><br>Study Design:<br>• Allocation: Randomized<br>• Intervention Model: Parallel Assignment<br>• Masking: Double (Participant, Investigator)<br>• Primary Purpose: Prevention<br><br>Outcome Measures:<br>• Changes of ALT(Alanine Transaminase)<br>• Changes of Liver function index<br>• Changes of fatty liver grade<br>• Changes of lipid metabolism index<br>• Changes of total antioxidant capacity<br>• Changes of imflammation index<br>• Changes of Multidimensional Fatigue Scale	Enrollment:<br>90<br><br>Age:<br>19 Years to 70 Years (Adult, Older Adult)<br><br>Sex:<br>All	• Chonbuk National University Hospital	• Other	Study Start:<br>July 2016<br><br>Primary Completion:<br>November 2016<br><br>Study Completion:<br>August 2017<br><br>First Posted:<br>August 24, 2017<br><br>Results First Posted:<br>No Results Posted<br><br>Last Update Posted:<br>February 5, 2018	• Clinical Trial Center for Functional Foods Chonbuk National University Hospital, Jeonju, Jeollabuk-do, Korea, Republic of

	NCT Number	Title	Other Names	Status	Conditions	Interventions	Characteristics	Population	Sponsor/ Collaborators	Funder Type	Dates	Locations
41	NCT00182780	American Ginseng in Treating Patients With Cancer-Related Fatigue<br><br>Study Documents:	Title Acronym:<br><br>Other Ids:<br>• NCCTG-N03CA<br>• NCI-2012-02670<br>• CDR0000440907	Completed	• Fatigue<br>• Unspecified Adult Solid Tumor, Protocol Specific	• Dietary Supplement: American ginseng<br>• Other: Placebo	Study Type: Interventional<br><br>Phase: Not Applicable<br><br>Study Design:<br>• Allocation: Randomized<br>• Intervention Model: Parallel Assignment<br>• Masking: Double (Participant, Investigator)<br>• Primary Purpose: Supportive Care<br><br>Outcome Measures:<br>• Fatigue by brief inventory at 4 and 8 weeks of treatment<br>• Sleep by Pittsburg Sleep Quality Inventory at 4 and 8 weeks of treatment<br>• Quality of life by North Central Cancer Treatment Group Uniscale at 4 and 8 weeks of treatment	Enrollment: 290<br><br>Age: 18 Years and older (Adult, Older Adult)<br><br>Sex: All	• Alliance for Clinical Trials in Oncology<br>• National Cancer Institute (NCI)	• Other<br>• NIH	Study Start: October 2005<br><br>Primary Completion: September 5, 2006<br><br>Study Completion: April 2010<br><br>First Posted: September 16, 2005<br><br>Results First Posted: No Results Posted<br><br>Last Update Posted: October 31, 2017	

	NCT Number	Title	Other Names	Status	Conditions	Interventions	Characteristics	Population	Sponsor/ Collaborators	Funder Type	Dates	Locations
42	NCT01542905	Stress Relief Effect of Korean Red Ginseng<br><br>Study Documents:	Title Acronym:<br><br>Other Ids: KG2010	Completed	•Stress Relief	•Dietary Supplement: Korean Red Ginseng<br>•Other: Placebo	Study Type: Interventional<br><br>Phase: Not Applicable<br><br>Study Design:<br>•Allocation: Randomized<br>•Intervention Model: Parallel Assignment<br>•Masking: Quadruple (Participant, Care Provider, Investigator, Outcomes Assessor)<br>•Primary Purpose: Treatment<br><br>Outcome Measures:<br>•Change from Baseline in Stress Scores at 8 Weeks<br>•Change from Baseline in Stress Scores at 4 Weeks<br>•Change from Baseline in Stress Scores at 1 Week<br>•Change from Baseline in Neurocognitive Function at 8 Weeks<br>•Change from Baseline in Brain Function, Chemistry, and Structure Measured Using Magnetic Resonance Imaging at 8 Weeks<br>•Change in Fatigue Scores at 8 Weeks<br>•Change in Fatigue Scores at 4 Weeks<br>•Change in Fatigue Scores at 1 Week<br>•Change in Depressive Scores at 8 Weeks<br>•Change in Depressive Scores at 4 Weeks<br>•and 5 more	Enrollment: 51<br><br>Age: 18 Years to 65 Years (Adult, Older Adult)<br><br>Sex: All	•Seoul National University Hospital	•Other	Study Start: May 2010<br><br>Primary Completion: April 2011<br><br>Study Completion: April 2011<br><br>First Posted: March 2, 2012<br><br>Results First Posted: No Results Posted<br><br>Last Update Posted: May 27, 2015	•Seoul National University Hospital, Seoul, Korea, Republic of

	NCT Number	Title	Other Names	Status	Conditions	Interventions	Characteristics	Population	Sponsor/ Collaborators	Funder Type	Dates	Locations
43	NCT00752895	American Ginseng Extract in Preventing Respiratory Infection and in Reducing Antibiotic Use in Patients With CLL<br><br>Study Documents:	Title Acronym:<br><br>Other Ids:<br>• IRB00006819<br>• U10CA081851<br>• REBACCCWFU983	Completed	• Leukemia	• Dietary Supplement: American ginseng<br>• Dietary Supplement: Placebo	Study Type: Interventional<br><br>Phase: Phase 2<br><br>Study Design:<br>• Allocation: Randomized<br>• Intervention Model: Parallel Assignment<br>• Masking: Quadruple (Participant, Care Provider, Investigator, Outcomes Assessor)<br>• Primary Purpose: Prevention<br><br>Outcome Measures:<br>• Acute Respiratory Infection (ARI) Days<br>• Number of Antibiotic Use Days	Enrollment: 293<br><br>Age: 18 Years and older (Adult, Older Adult)<br><br>Sex: All	• Wake Forest University Health Sciences<br>• National Cancer Institute (NCI)	• Other<br>• NIH	Study Start: September 1, 2008<br><br>Primary Completion: December 1, 2008<br><br>Study Completion: June 1, 2009<br><br>First Posted: September 16, 2008<br><br>Results First Posted: November 1, 2015<br><br>Last Update Posted: September 28, 2021	• Providence Saint Joseph Medical Center - Burbank, Burbank, California, United States<br>• St. Joseph Hospital Regional Cancer Center - Orange, Orange, California, United States<br>• Helen and Harry Gray Cancer Center at Hartford Hospital, Hartford, Connecticut, United States<br>• Saint Anthony's Hospital at Saint Anthony's Health Center, Alton, Illinois, United States<br>• Rush-Copley Cancer Care Center, Aurora, Illinois, United States<br>• St. Joseph Medical Center, Bloomington, Illinois, United States<br>• Graham Hospital, Canton, Illinois, United States<br>• Memorial Hospital, Carthage, Illinois, United States<br>• Eureka Community Hospital, Eureka, Illinois, United States<br>• Evanston Northwestern Healthcare - Evanston Hospital, Evanston, Illinois, United States<br>• and 115 more

	NCT Number	Title	Other Names	Status	Conditions	Interventions	Characteristics	Population	Sponsor/ Collaborators	Funder Type	Dates	Locations
44	NCT00719563	American Ginseng in Treating Patients With Fatigue Caused by Cancer<br><br>Study Documents:	Title Acronym:<br><br>Other Ids:<br>• NCCTG-N07C2<br>• NCI-2009-00872<br>• CDR0000597665	Completed	• Chronic Myeloproliferative Disorders<br>• Fatigue<br>• Leukemia<br>• Lymphoma<br>• Lymphoproliferative Disorder<br>• Multiple Myeloma and Plasma Cell Neoplasm<br>• Myelodysplastic Syndromes<br>• Myelodysplastic/ Myeloproliferative Neoplasms<br>• Precancerous Condition<br>• Unspecified Adult Solid Tumor, Protocol Specific	• Drug: American ginseng<br>• Other: placebo	Study Type:<br>Interventional<br><br>Phase:<br>Phase 3<br><br>Study Design:<br>• Allocation: Randomized<br>• Intervention Model: Parallel Assignment<br>• Masking: Double (Participant, Investigator)<br>• Primary Purpose: Supportive Care<br><br>Outcome Measures:<br>• Change From Baseline to Week 4 in the General Subscale of the MFSI-SF<br>• Number of Treatment Related Grade 2 to 3 Adverse Events >=1% Incidence<br>• Change From Baseline to Week 4 in the Impact on Physical, Mental, and Emotional States and Vigor as Measured by Other Subscales of the MFSI-SF<br>• Change From Baseline to Week 4 Fatigue as Measured by the BFI and Linear Analogue Scale of Fatigue<br>• Change From Baseline to Week 4 Vigor/Activity and Fatigue-inertia as Measured by POMS<br>• Change From Baseline to Week 4 for the Impact on Stress as Measured by Perceived Stress Scale (PSS)<br>• Change From Baseline to Week 8 in the Impact on General, Physical, Mental, and Emotional States and Vigor as Measured by Other Subscales of the MFSI-SF<br>• Change From Baseline to Week 8 Fatigue as Measured by the BFI and Linear Analogue Scale of Fatigue<br>• Change From Baseline to Week 8 Vigor/Activity and Fatigue-inertia as Measured by POMS<br>• Change From Baseline to Week 8 for the Impact on	Enrollment:<br>364<br><br>Age:<br>18 Years and older (Adult, Older Adult)<br><br>Sex:<br>All	• Alliance for Clinical Trials in Oncology<br>• National Cancer Institute (NCI)	• Other<br>• NIH	Study Start:<br>October 2008<br><br>Primary Completion:<br>August 2011<br><br>Study Completion:<br>August 2013<br><br>First Posted:<br>July 21, 2008<br><br>Results First Posted:<br>August 6, 2014<br><br>Last Update Posted:<br>February 9, 2017	• Mayo Clinic Scottsdale, Scottsdale, Arizona, United States<br>• Aurora Presbyterian Hospital, Aurora, Colorado, United States<br>• Boulder Community Hospital, Boulder, Colorado, United States<br>• Penrose Cancer Center at Penrose Hospital, Colorado Springs, Colorado, United States<br>• St. Anthony Central Hospital, Denver, Colorado, United States<br>• Porter Adventist Hospital, Denver, Colorado, United States<br>• Presbyterian - St. Luke's Medical Center, Denver, Colorado, United States<br>• St. Joseph Hospital, Denver, Colorado, United States<br>• Rose Medical Center, Denver, Colorado, United States<br>• CCOP - Colorado Cancer Research Program, Denver, Colorado, United States<br>• and 316 more

	NCT Number	Title	Other Names	Status	Conditions	Interventions	Characteristics	Population	Sponsor/ Collaborators	Funder Type	Dates	Locations
45	NCT02923453	Effect of Ginseng in Type 2 Diabetes<br>Study Documents:	Title Acronym:<br>Other Ids: CNT 2000	Completed	• Type II Diabetes Control	• Dietary Supplement: CNT 2000 American ginseng extract<br>• Dietary Supplement: Placebo	Study Type: Interventional<br>Phase: Phase 2<br>Study Design:<br>• Allocation: Randomized<br>• Intervention Model: Crossover Assignment<br>• Masking: Double (Participant, Care Provider)<br>• Primary Purpose: Treatment<br>Outcome Measures:<br>• HbA1c<br>• Fasting blood glucose<br>• Fasting blood insulin<br>• Blood pressure<br>• serum nitrates/nitrites (NOx)<br>• Plasminogen activator inhibitor-1 (PAI-1)<br>• Alanine amino-transferase (ALT)<br>• serum creatinine	Enrollment: 23<br>Age: 45 Years to 75 Years (Adult, Older Adult)<br>Sex: All	• Unity Health Toronto	• Other	Study Start: March 1998<br>Primary Completion: December 2001<br>Study Completion: August 2002<br>First Posted: October 4, 2016<br>Results First Posted: No Results Posted<br>Last Update Posted: October 4, 2016	• : Clinical Nutrition and Risk Factor Modification Centre, St. Michael's Hospital, Toronto, Ontario, Canada
46	NCT00219960	The Effect of North American Ginseng on Blood Pressure in Individuals With Hypertension<br>Study Documents:	Title Acronym:<br>Other Ids:<br>• RFMC-0001-77<br>• SR-7093	Completed	• Hypertension	• Drug: North American Ginseng (Panax Quinquefolius)	Study Type: Interventional<br>Phase: Phase 3<br>Study Design:<br>• Allocation: Randomized<br>• Intervention Model: Crossover Assignment<br>• Masking: Double<br>• Primary Purpose: Treatment<br>Outcome Measures:<br>• Mean 24 Hour Ambulatory Blood Pressure<br>• Mean Daytime Ambulatory Blood Pressure<br>• Mean Nighttime Ambulatory Blood Pressure<br>• Cystatin C	Enrollment: 52<br>Age: 18 Years to 85 Years (Adult, Older Adult)<br>Sex: All	• Risk Factor Modification Centre<br>• Ontario Ministry of Agriculture, Food and Rural Affairs<br>• Ontario Ginseng Growers Association	• Other	Study Start: April 2001<br>Primary Completion:<br>Study Completion: October 2003<br>First Posted: September 22, 2005<br>Results First Posted: No Results Posted<br>Last Update Posted: September 22, 2005	• Risk Factor Modification Centre, St. Michael's Hospital, Toronto, Ontario, Canada

	NCT Number	Title	Other Names	Status	Conditions	Interventions	Characteristics	Population	Sponsor/ Collaborators	Funder Type	Dates	Locations
47	NCT01478009	Efficacy of an Extract of Concentrated Korean Red Ginseng for Preventing Upper Respiratory Tract Infections<br>Study Documents:	Title Acronym:<br>Other Ids: IJRG-INFL-KRG	Completed	•Healthy Subjects	•Dietary Supplement: Korean red ginseng<br>•Dietary Supplement: Placebo	Study Type: Interventional<br>Phase: •Phase 2 •Phase 3<br>Study Design: •Allocation: Randomized •Intervention Model: Parallel Assignment •Masking: Double (Participant, Investigator) •Primary Purpose: Prevention<br>Outcome Measures: •Frequency of ILI(Influenza Like Illness) •Symptom Severity of All Colds •Total Number of Days of Symptoms and Duration of All Colds	Enrollment: 100<br>Age: 30 Years to 70 Years (Adult, Older Adult)<br>Sex: All	•Chonbuk National University Hospital	•Other	Study Start: November 2010<br>Primary Completion: December 2011<br>Study Completion: December 2011<br>First Posted: November 23, 2011<br>Results First Posted: December 25, 2012<br>Last Update Posted: December 25, 2012	•Clinical Trial Center for Functional Foods; Chonbuk National University Hospital, Jeonju, Jeollabuk-do, Korea, Republic of
48	NCT03136770	A Randomized, Open-label, Two-way Crossover Study to Assess the Pharmacokinetics and Safety of CK-30 600 mg (Compound K)<br>Study Documents:	Title Acronym:<br>Other Ids: CK-30	Completed	•Pharmacokinetics	•Dietary Supplement: CK-30<br>•Dietary Supplement: Red ginseng extracts	Study Type: Interventional<br>Phase: Not Applicable<br>Study Design: •Allocation: Randomized •Intervention Model: Crossover Assignment •Masking: Quadruple (Participant, Care Provider, Investigator, Outcomes Assessor) •Primary Purpose: Basic Science<br>Outcome Measures: •Pharmacokinetics (Cmax) •Pharmacokinetics (AUClast) •Safety and tolerability (Number of participants with treatment-related adverse events)	Enrollment: 25<br>Age: 19 Years to 45 Years (Adult)<br>Sex: Male	•Seoul National University Hospital	•Other	Study Start: February 15, 2017<br>Primary Completion: April 10, 2017<br>Study Completion: April 10, 2017<br>First Posted: May 2, 2017<br>Results First Posted: No Results Posted<br>Last Update Posted: May 2, 2017	•Seoul National University Hospital Clinical Trials Center, Seoul, Korea, Republic of

	NCT Number	Title	Other Names	Status	Conditions	Interventions	Characteristics	Population	Sponsor/ Collaborators	Funder Type	Dates	Locations
49	NCT01951443	Korean Red Ginseng Rg3 Extract on Arterial Stiffness and Blood Pressure<br>Study Documents:	Title Acronym: KRAB<br>Other Ids: KRAB	Completed	• Arterial Stiffness, Blood Pressure	• Dietary Supplement: Ginseng<br>• Dietary Supplement: Wheat Bran	Study Type: Interventional<br>Phase: Phase 1<br>Study Design:<br>• Allocation: Randomized<br>• Intervention Model: Crossover Assignment<br>• Masking: Triple (Participant, Investigator, Outcomes Assessor)<br>• Primary Purpose: Treatment<br>Outcome Measures:<br>• To evaluate the acute effect of Rg3-KRG on arterial stiffness in healthy adult volunteers, as measured by aortic augmentation index (AIx).<br>• To evaluate the acute effect of Rg3-KRG on aortic and brachial blood pressure in healthy adult volunteers.	Enrollment: 24<br>Age: 18 Years to 70 Years (Adult, Older Adult)<br>Sex: All	• Unity Health Toronto	• Other	Study Start: August 2013<br>Primary Completion: February 2014<br>Study Completion:<br>First Posted: September 26, 2013<br>Results First Posted: No Results Posted<br>Last Update Posted: April 28, 2014	• Risk Factor Modification Centre, St. Michael's Hospital, Toronto, Ontario, Canada
50	NCT00728221	Evaluation and Standardization of Ginseng and it's Components for Blood Pressure Regulation<br>Study Documents:	Title Acronym: ESGC<br>Other Ids: 118328	Completed	• Hypertension<br>• Blood Pressure<br>• Endothelial Function	• Dietary Supplement: Korean Red Ginseng<br>• Dietary Supplement: Korean Red Ginseng (Panax ginseng)<br>• Dietary Supplement: Cornstarch<br>• Dietary Supplement: Korean Red Ginseng (Panax Ginseng)	Study Type: Interventional<br>Phase: Phase 2<br>Study Design:<br>• Allocation: Randomized<br>• Intervention Model: Crossover Assignment<br>• Masking: Quadruple (Participant, Care Provider, Investigator, Outcomes Assessor)<br>• Primary Purpose: Prevention<br>Outcome Measures:<br>• Flow-mediated dilation of the brachial artery<br>• Augmentation Index<br>• Nitric Oxide and Cyclic GMP	Enrollment: 17<br>Age: 18 Years to 70 Years (Adult, Older Adult)<br>Sex: All	• Unity Health Toronto<br>• Heart and Stroke Foundation of Canada	• Other	Study Start: November 2007<br>Primary Completion: April 2008<br>Study Completion: April 2008<br>First Posted: August 5, 2008<br>Results First Posted: No Results Posted<br>Last Update Posted: January 6, 2017	• St. Michael's Hospital Clinical Nutrition and Risk Factor Modification Centre, Toronto, Ontario, Canada

	NCT Number	Title	Other Names	Status	Conditions	Interventions	Characteristics	Population	Sponsor/ Collaborators	Funder Type	Dates	Locations
51	NCT00730951	The Evaluation and Standardization of Ginseng and Its Components for Blood Pressure Regulation<br><br>Study Documents:	Title Acronym:<br><br>Other Ids: 107460	Completed	•Hypertension	•Dietary Supplement: Korean Red Ginseng<br>•Dietary Supplement: Corn Starch	Study Type: Interventional<br><br>Phase: Phase 2<br><br>Study Design:<br>•Allocation: Randomized<br>•Intervention Model: Crossover Assignment<br>•Masking: Double (Participant, Investigator)<br>•Primary Purpose: Treatment<br><br>Outcome Measures:<br>•Blood Pressure will be measured with an Ambulatory Blood Pressure Monitor<br>•Blood samples will be drawn and tested for Nitric Oxide levels.	Enrollment: 18<br><br>Age: 18 Years to 70 Years (Adult, Older Adult)<br><br>Sex: All	•Unity Health Toronto<br>•Heart and Stroke Foundation of Ontario	•Other	Study Start: June 2007<br><br>Primary Completion: January 2008<br><br>Study Completion: March 2008<br><br>First Posted: August 8, 2008<br><br>Results First Posted: No Results Posted<br><br>Last Update Posted: July 31, 2015	•Clinical Nutrition and Risk Factor Modification Centre, Toronto, Ontario, Canada
52	NCT01913210	Effect of Ginseng on Blood Pressure<br><br>Study Documents:	Title Acronym:<br><br>Other Ids: MetaBPG	Completed	•Blood Pressure	•Dietary Supplement: Ginseng	Study Type: Observational<br><br>Phase:<br><br>Study Design: Time Perspective: Prospective<br><br>Outcome Measures:<br>•Systolic Blood Pressure<br>•Diastolic Blood Pressure<br>•Mean Arterial Pressure	Enrollment: 500<br><br>Age: Child, Adult, Older Adult<br><br>Sex: All	•Unity Health Toronto	•Other	Study Start: April 2013<br><br>Primary Completion: May 2013<br><br>Study Completion: January 2016<br><br>First Posted: July 31, 2013<br><br>Results First Posted: No Results Posted<br><br>Last Update Posted: March 3, 2016	•The Toronto 3D Knowledge Synthesis and Clinical Trials Unit, Clinical Nutrition and Risk Factor Modification Centre, Toronto, Ontario, Canada

	NCT Number	Title	Other Names	Status	Conditions	Interventions	Characteristics	Population	Sponsor/ Collaborators	Funder Type	Dates	Locations
53	NCT00367926	The Effect of American Ginseng Root and Its Components on Glycemia in Healthy Individuals<br>Study Documents:	Title Acronym:<br>Other Ids:<br>•RFMC-0001-103<br>•MOP- 62943	Completed	•Hyperglycemia	•Drug: American ginseng root / polysaccharides	Study Type: Interventional<br>Phase: Phase 3<br>Study Design:<br>•Allocation: Randomized<br>•Intervention Model: Crossover Assignment<br>•Masking: Double<br>•Primary Purpose: Treatment<br>Outcome Measures:<br>•incremental area under the postprandial glucose curve<br>•incremental peak postprandial glucose	Enrollment: 12<br>Age: 16 Years to 65 Years (Child, Adult, Older Adult)<br>Sex: All	•Risk Factor Modification Centre<br>•Canadian Institutes of Health Research (CIHR)<br>•Ontario Ginseng Growers Association	•Other	Study Start: June 2005<br>Primary Completion:<br>Study Completion: July 2005<br>First Posted: August 23, 2006<br>Results First Posted: No Results Posted<br>Last Update Posted: August 23, 2006	•Risk Factor Modification Centre, Toronto, Ontario, Canada
54	NCT01854164	Efficacy and Safety of Hydrolyzed Ginseng Extract on Impaired Fasting Glucose<br>Study Documents:	Title Acronym:<br>Other Ids: Ilhwa-FG-001	Completed	•Hyperglycemia	•Dietary Supplement: HGE (hydrolyzed ginseng extract)<br>•Dietary Supplement: Placebo	Study Type: Interventional<br>Phase: Not Applicable<br>Study Design:<br>•Allocation: Randomized<br>•Intervention Model: Parallel Assignment<br>•Masking: Double (Participant, Investigator)<br>•Primary Purpose: Prevention<br>Outcome Measures:<br>•Changes of blood glucose during OGTT(oral glucose tolerance test)<br>•Changes of blood insulin during OGTT.<br>•Changes of glycated albumin, fructosamine, and lipid profile	Enrollment: 20<br>Age: 20 Years to 70 Years (Adult, Older Adult)<br>Sex: All	•Chonbuk National University Hospital	•Other	Study Start: June 2009<br>Primary Completion: December 2009<br>Study Completion: December 2009<br>First Posted: May 15, 2013<br>Results First Posted: No Results Posted<br>Last Update Posted: March 15, 2016	•Clinical Trial Center for Functional Foods, Chonbuk National University Hospital, Jeonju, Jeollabuk-do, Korea, Republic of

	NCT Number	Title	Other Names	Status	Conditions	Interventions	Characteristics	Population	Sponsor/ Collaborators	Funder Type	Dates	Locations
55	NCT01479426	A Trial to Evaluate the Efficacy and Safety of EFLA400 Korea Red Ginseng Extract on Sexual Function in Men With Erectile Dysfunction<br><br>Study Documents:	Title Acronym:<br><br>Other Ids:<br>LOTTE-MS-EFLA400	Completed	• Healthy Subjects(Only Men)	• Dietary Supplement: EFLA400<br>• Dietary Supplement: Placebo	Study Type:<br>Interventional<br><br>Phase:<br>Not Applicable<br><br>Study Design:<br>• Allocation: Randomized<br>• Intervention Model: Parallel Assignment<br>• Masking: Double (Participant, Investigator)<br>• Primary Purpose: Prevention<br><br>Outcome Measures:<br>• Changes in EF(Erectile Function) Domain<br>• Changes in MSHQ (Male Sexual Health Questionnaire)<br>• GEAQ (Global Efficacy Assessment Question)<br>• Changes in Uroflowmetry(Max Flow Rate)<br>• Changes in IIEF(International Index of Erectile Function)-Total Domain	Enrollment:<br>80<br><br>Age:<br>19 Years to 70 Years (Adult, Older Adult)<br><br>Sex:<br>Male	• Chonbuk National University Hospital	• Other	Study Start:<br>December 2010<br><br>Primary Completion:<br>December 2011<br><br>Study Completion:<br>February 2012<br><br>First Posted:<br>November 24, 2011<br><br>Results First Posted:<br>September 23, 2019<br><br>Last Update Posted:<br>September 23, 2019	• Clinical Trial Center for Functional Foods; Chonbuk National University Hospital, Jeonju, Jeollabuk-do, Korea, Republic of

	NCT Number	Title	Other Names	Status	Conditions	Interventions	Characteristics	Population	Sponsor/ Collaborators	Funder Type	Dates	Locations
56	NCT01201187	Efficacy and Safety Study of Combination of Ginkgo Extract and Ginseng Extract(YY-162)in Children With ADHD<br><br>Study Documents:	Title Acronym: yuyu<br><br>Other Ids: YY-162	Completed	•Mental Disorders	•Drug: YY-162<br>•Drug: Placebo	Study Type: Interventional<br><br>Phase: Phase 3<br><br>Study Design:<br>•Allocation: Randomized<br>•Intervention Model: Parallel Assignment<br>•Masking: Double (Participant, Investigator)<br>•Primary Purpose: Treatment<br><br>Outcome Measures:<br>•Korea-ADHD Rating scale<br>•IOWA Conner's rating scale<br>•Clinical global Impression(Severity and Improvement)<br>•Advanced Test of Attention<br>•Children's color trails test and stroop test<br>•Intelligence test(from KEDI-WISC)	Enrollment: 144<br><br>Age: 6 Years to 12 Years (Child)<br><br>Sex: All	•Yuyu Pharma, Inc.	•Industry	Study Start: March 2010<br><br>Primary Completion: April 2010<br><br>Study Completion: April 2011<br><br>First Posted: September 14, 2010<br><br>Results First Posted: No Results Posted<br><br>Last Update Posted: February 25, 2019	•Hallym University Hospital, Anyang-si, Korea, Republic of<br>•Inje University Ilsan Paik Hospital, Goyang-Si, Korea, Republic of<br>•Seoul National University Hospital, Seoul, Korea, Republic of

	NCT Number	Title	Other Names	Status	Conditions	Interventions	Characteristics	Population	Sponsor/ Collaborators	Funder Type	Dates	Locations
57	NCT01536210	Efficacy and Safety Study of Combination of Ginkgo Extract and Ginseng Extract in Children With ADHD(Attention Deficit Hyperactivity Disorder)<br><br>Study Documents:	Title Acronym: ADHD<br><br>Other Ids: YY-162 (b)	Completed	• Mental Disorders	• Drug: YY-162<br>• Drug: Placebo	Study Type: Interventional<br><br>Phase: Phase 3<br><br>Study Design:<br>• Allocation: Randomized<br>• Intervention Model: Parallel Assignment<br>• Masking: Triple (Participant, Care Provider, Investigator)<br>• Primary Purpose: Treatment<br><br>Outcome Measures:<br>• Korea-ADHD Rating scale<br>• IOWA conner's rating scale<br>• Clinical Global Impression(Severity and Improvement)<br>• Advanced Test of Attention<br>• children's color trails test and stroop test<br>• Intelligence test(from KEDI-WISC)	Enrollment: 144<br><br>Age: 6 Years to 15 Years (Child)<br><br>Sex: All	• Yuyu Pharma, Inc.	• Industry	Study Start: December 2011<br><br>Primary Completion: August 2012<br><br>Study Completion: December 2012<br><br>First Posted: February 20, 2012<br><br>Results First Posted: No Results Posted<br><br>Last Update Posted: February 25, 2019	• Hallym University Hospital, Anyang-si, Korea, Republic of<br>• Inje University Ilsan Paik Hospital, Goyang-Si, Korea, Republic of<br>• Seoul National University Hospital, Seoul, Korea, Republic of

	NCT Number	Title	Other Names	Status	Conditions	Interventions	Characteristics	Population	Sponsor/ Collaborators	Funder Type	Dates	Locations
58	NCT05055427	Efficacy Evaluation of Shen Cao Gan Jiang Tang on Mild and Moderate COVID-19 Patients<br><br>Study Documents:	Title Acronym:<br><br>Other Ids:<br>458/H###-#HYD	Recruiting	•COVID-19 Respiratory Infection<br>•Herbal Medicine	•Drug: Shen Cao Gan Jiang Tang (Gan Cao Gan Jiang Tang with Ginseng)	Study Type:<br>Interventional<br><br>Phase:<br>•Phase 2<br>•Phase 3<br><br>Study Design:<br>•Allocation: Randomized<br>•Intervention Model: Parallel Assignment<br>•Masking: None (Open Label)<br>•Primary Purpose: Treatment<br><br>Outcome Measures:<br>•Duration of symptoms of COVID-19<br>•The severity of the COVID-19 total and individual symptoms<br>•Rate of progression to disease severity<br>•The time required to meet discharge standards<br>•National Early Warning Score 2 (NEWS2)<br>•Cycle threshold (CT)<br>•Duration of SARS-CoV-2 virus infection<br>•Mortality rate<br>•Number of participants clinically recovered<br>•Paracetamol/Ibuprofen intake<br>•Safety evaluation	Enrollment:<br>300<br><br>Age:<br>18 Years to 64 Years (Adult)<br><br>Sex:<br>All	•University of Medicine and Pharmacy at Ho Chi Minh City	•Other	Study Start:<br>August 20, 2021<br><br>Primary Completion:<br>March 20, 2022<br><br>Study Completion:<br>August 20, 2022<br><br>First Posted:<br>September 24, 2021<br><br>Results First Posted:<br>No Results Posted<br><br>Last Update Posted:<br>March 15, 2022	•University of Medicine and Pharmacy at Ho Chi Minh City, Ho Chi Minh, Vietnam

	NCT Number	Title	Other Names	Status	Conditions	Interventions	Characteristics	Population	Sponsor/ Collaborators	Funder Type	Dates	Locations
59	NCT00029692	Effects of Ginseng and Ginkgo on Drug Disposition in Man<br><br>Study Documents:	Title Acronym:<br><br>Other Ids: R01AT000842-01	Completed	• Healthy	• Drug: Ginseng<br>• Drug: Ginkgo	Study Type: Interventional<br><br>Phase: Phase 2<br><br>Study Design:<br>• Allocation: Randomized<br>• Masking: Double<br>• Primary Purpose: Treatment<br><br>Outcome Measures:	Enrollment: 60<br><br>Age: 20 Years and older (Adult, Older Adult)<br><br>Sex: All	• National Center for Complementary and Integrative Health (NCCIH)	• NIH	Study Start: March 2002<br><br>Primary Completion:<br><br>Study Completion: March 2005<br><br>First Posted: January 21, 2002<br><br>Results First Posted: No Results Posted<br><br>Last Update Posted: July 26, 2006	• University of Kansas Medical Center, Kansas City, Kansas, United States

	NCT Number	Title	Other Names	Status	Conditions	Interventions	Characteristics	Population	Sponsor/ Collaborators	Funder Type	Dates	Locations
60	NCT02396615	SATIN WP4 Acute Effect on Appetite of Pineapple Juice With Viscofiber and Red Ginseng<br><br>Study Documents:	Title Acronym: SATIN<br><br>Other Ids: B318	Completed	• Healthy	• Dietary Supplement: SATIN pineapple<br>• Dietary Supplement: SATIN pineapple control	Study Type: Interventional<br><br>Phase: Not Applicable<br><br>Study Design:<br>• Allocation: Randomized<br>• Intervention Model: Crossover Assignment<br>• Masking: Triple (Participant, Investigator, Outcomes Assessor)<br>• Primary Purpose: Basic Science<br><br>Outcome Measures:<br>• Ad libitum energy intake<br>• VAS score for appetite sensation - hunger<br>• Eating behaviour questionnaire - binge eating scale<br>• Eating behaviour questionnaire - Control of Eating<br>• Eating behaviour questionnaire - three factor eating questionnaire<br>• Eating behaviour questionnaire- power of food<br>• VAS score for appetite sensation - fullness<br>• VAS score for appetite sensation - Desire to eat<br>• VAS score for appetite sensation - prospective consumption	Enrollment: 30<br><br>Age: 18 Years to 55 Years (Adult)<br><br>Sex: All	• University of Copenhagen<br>• University of Leeds<br>• University of Liverpool	• Other	Study Start: February 2015<br><br>Primary Completion: April 2015<br><br>Study Completion: April 2015<br><br>First Posted: March 24, 2015<br><br>Results First Posted: No Results Posted<br><br>Last Update Posted: April 22, 2015	• University of Copenhagen, Copenhagen, Frederiksberg, Denmark

	NCT Number	Title	Other Names	Status	Conditions	Interventions	Characteristics	Population	Sponsor/ Collaborators	Funder Type	Dates	Locations
61	NCT01911663	The Effect of Korean Red Ginseng Supplementation on Glucose Control<br>Study Documents:	Title Acronym:<br>Other Ids: KGC-CKJ-PC	Completed	•Diabetes	•Dietary Supplement: KRG<br>•Dietary Supplement: Placebo	Study Type: Interventional<br>Phase: Phase 4<br>Study Design:<br>•Allocation: Randomized<br>•Intervention Model: Parallel Assignment<br>•Masking: Quadruple (Participant, Care Provider, Investigator, Outcomes Assessor)<br>•Primary Purpose: Prevention<br>Outcome Measures:<br>•change from baseline in glucose<br>•change from baseline in insulin<br>•change from baseline in C-peptide	Enrollment: 60<br>Age: 20 Years to 70 Years (Adult, Older Adult)<br>Sex: All	•Yonsei University	•Other	Study Start: November 2011<br>Primary Completion: February 2012<br>Study Completion: March 2012<br>First Posted: July 30, 2013<br>Results First Posted: No Results Posted<br>Last Update Posted: July 30, 2013	•Laboratory of clinical Nutrigenetics/Nutrigenomic, Seoul, Korea, Republic of
62	NCT01734005	Efficacy and Safety of Red Ginseng on Decrement of Body Fat<br>Study Documents:	Title Acronym:<br>Other Ids: ARIMED-BF-GC	Completed	•Obesity	•Dietary Supplement: Red Ginseng<br>•Dietary Supplement: Placebo	Study Type: Interventional<br>Phase: Not Applicable<br>Study Design:<br>•Allocation: Randomized<br>•Intervention Model: Parallel Assignment<br>•Masking: Double (Participant, Investigator)<br>•Primary Purpose: Prevention<br>Outcome Measures:<br>•Changes in Body Fat Mass<br>•Changes in Percent Body Fat<br>•Changes in weight<br>•Changes in BMI(body mass index)	Enrollment: 60<br>Age: 19 Years to 65 Years (Adult, Older Adult)<br>Sex: All	•Chonbuk National University Hospital	•Other	Study Start: October 10, 2012<br>Primary Completion: August 2013<br>Study Completion: August 2013<br>First Posted: November 27, 2012<br>Results First Posted: No Results Posted<br>Last Update Posted: August 28, 2019	•Clinical Trial Center for Functional Foods; Chonbuk National University Hospital, Jeonju, Jeollabuk-do, Korea, Republic of

	NCT Number	Title	Other Names	Status	Conditions	Interventions	Characteristics	Population	Sponsor/ Collaborators	Funder Type	Dates	Locations
63	NCT01826409	Hypoglycemic Effects of Fermented Red Ginseng in Subject With Impaired Fasting Glucose or Type 2 Diabetes<br><br>Study Documents:	Title Acronym:<br><br>Other Ids: WKP-FG7070-001	Completed	•Impaired Glucose or Type 2 Diabetes	•Dietary Supplement: Fermented Red Ginseng<br>•Dietary Supplement: Placebo	Study Type: Interventional<br><br>Phase:<br>•Phase 2<br>•Phase 3<br><br>Study Design:<br>•Allocation: Randomized<br>•Intervention Model: Parallel Assignment<br>•Masking: Quadruple (Participant, Care Provider, Investigator, Outcomes Assessor)<br>•Primary Purpose: Prevention<br><br>Outcome Measures:<br>•Glucose profiles during meal tolerance test<br>•Change in lipid profiles	Enrollment: 42<br><br>Age: 20 Years to 75 Years (Adult, Older Adult)<br><br>Sex: All	•Chonbuk National University Hospital	•Other	Study Start: March 2008<br><br>Primary Completion: September 2009<br><br>Study Completion:<br><br>First Posted: April 8, 2013<br><br>Results First Posted: No Results Posted<br><br>Last Update Posted: August 29, 2013	•Clincial Trial Center for Functional Foods, Jeonju, Jeollabok-do, Korea, Republic of

	NCT Number	Title	Other Names	Status	Conditions	Interventions	Characteristics	Population	Sponsor/ Collaborators	Funder Type	Dates	Locations
64	NCT01006460	A Study With Arctic Root Compared With the Extract When Combined With Schizandra and Russian Root (Adapt 232), Standardized Ginseng Extract and Placebo Regarding Impact on the Level of Energy, Ability to Work Under Stress, Quality of Life and Wellbeing, in Middleaged Women Who Are Still Employed<br><br>Study Documents:	Title Acronym:<br><br>Other Ids: SHR5/DK	Completed	• Depression<br>• Stress	• Drug: Rhodiola rosea, L<br>• Drug: Rhodiola rosea, L., Eleutherococcus senticosus, Schisandra chinensis<br>• Drug: Panax ginseng<br>• Drug: Placebo - dark brown sugar	Study Type: Interventional<br><br>Phase: Not Applicable<br><br>Study Design:<br>• Allocation: Randomized<br>• Intervention Model: Parallel Assignment<br>• Masking: Quadruple (Participant, Care Provider, Investigator, Outcomes Assessor)<br>• Primary Purpose: Treatment<br><br>Outcome Measures:<br>• Psychological parameters: changes in cognitive functions as measured by the D2 Test of Attention<br>• Depression parameters: changes in depressive state as measured by the using Hamilton Depression Rating Scale (HAM-D) and Bechs Depression Inventory (BDI).<br>• Quality of Life parameters: changes in quality of life as measured by the SF-36 scale, Danish Stress Profile (SP) test and questions regarding various aspects of well-being as formulated in a non-validated VAS scale	Enrollment: 200<br><br>Age: 40 Years and older (Adult, Older Adult)<br><br>Sex: Female	• Frederiksberg University Hospital	• Other	Study Start: November 2009<br><br>Primary Completion: July 2010<br><br>Study Completion: August 2010<br><br>First Posted: November 1, 2009<br><br>Results First Posted: No Results Posted<br><br>Last Update Posted: August 27, 2010	• Department of Clinical Biochemistry, Frederikbergs Hospital, Frederiksberg, Denmark

	NCT Number	Title	Other Names	Status	Conditions	Interventions	Characteristics	Population	Sponsor/ Collaborators	Funder Type	Dates	Locations
65	NCT02161198	Efficacy and Safety Study of Ginseng Polysaccharide Extract<br><br>Study Documents:	Title Acronym:<br><br>Other Ids:<br>Y-75	Completed	• Healthy	• Dietary Supplement: Y-75<br>• Dietary Supplement: Placebo	Study Type:<br>Interventional<br><br>Phase:<br>Phase 2<br><br>Study Design:<br>• Allocation: Randomized<br>• Intervention Model: Parallel Assignment<br>• Masking: Quadruple (Participant, Care Provider, Investigator, Outcomes Assessor)<br>• Primary Purpose: Treatment<br><br>Outcome Measures:<br>• Mean percentage change in natural killer cell activity from a baseline level<br>• Changes from baseline in phagocytic activity of macrophages and polymorphonuclear cells<br>• Changes from baseline in serum levels of tumor necrosis factor-alpha and interleukin-12<br>• Number of patients with laboratory abnormalities<br>• Number of participants with serious and non-serious adverse events<br>• Number of patients with abnormal findings in physical examination and vital signs	Enrollment:<br>72<br><br>Age:<br>50 Years to 75 Years (Adult, Older Adult)<br><br>Sex:<br>All	• Seoul St. Mary's Hospital<br>• Health Biomed Inc.	• Other<br>• Industry	Study Start:<br>September 2012<br><br>Primary Completion:<br>April 2013<br><br>Study Completion:<br>December 2013<br><br>First Posted:<br>June 11, 2014<br><br>Results First Posted:<br>No Results Posted<br><br>Last Update Posted:<br>June 11, 2014	• Seoul St. Mary's Hospital, The Catholic University of Korea, Seoul, Korea, Republic of

	NCT Number	Title	Other Names	Status	Conditions	Interventions	Characteristics	Population	Sponsor/ Collaborators	Funder Type	Dates	Locations
66	NCT00255307	Pilot Evaluation of CVT-E002 in Pediatric Upper Respiratory Tract Infection.<br>Study Documents:	Title Acronym:<br>Other Ids: CVT-E002-2005-4	Completed	•Upper Respiratory Infection	•Drug: CVT-E002 ginseng extract	Study Type: Interventional<br>Phase: Phase 2<br>Study Design: •Allocation: Randomized •Intervention Model: Parallel Assignment •Masking: Double •Primary Purpose: Treatment<br>Outcome Measures: •To establish preliminary estimate of treatment effect of two doses of American ginseng extract, CVT-E002, in reducing severity and duration of URTI in children •To document adverse events related to the short course of American ginseng extract, CVT-E002, in children	Enrollment: 75<br>Age: 3 Years to 12 Years (Child)<br>Sex: All	•CV Technologies •University of Alberta •Capital Health, Canada •Afexa Life Sciences Inc	•Industry •Other	Study Start: November 2005<br>Primary Completion:<br>Study Completion: April 2006<br>First Posted: November 18, 2005<br>Results First Posted: No Results Posted<br>Last Update Posted: June 18, 2007	•Stollery Children's Hospital, Edmonton, Alberta, Canada •Misericordia Child Health Clinic, Edmonton, Alberta, Canada
67	NCT02202382	Effects of Korean Red Ginseng on Male Infertility<br>Study Documents:	Title Acronym:<br>Other Ids: KGR study	Completed	•Male Infertility	•Drug: Korean Red Ginseng, Varicocelectomy •Drug: Placebo •Procedure: Varicocelectomy	Study Type: Interventional<br>Phase: Phase 4<br>Study Design: •Allocation: Randomized •Intervention Model: Parallel Assignment •Masking: Double (Participant, Investigator) •Primary Purpose: Treatment<br>Outcome Measures: •Sperm concentration •Sperm motility •Sperm morphology •Sperm viability •Serum concentrations of FSH •Serum concentrations of LH •Serum concentrations of testosterone	Enrollment: 80<br>Age: 25 Years to 45 Years (Adult)<br>Sex: Male	•Pusan National University Hospital	•Other	Study Start: April 2011<br>Primary Completion: January 2012<br>Study Completion:<br>First Posted: July 29, 2014<br>Results First Posted: No Results Posted<br>Last Update Posted: July 31, 2014	•Department of Urology, Pusan National University Hospital, Busan, Korea, Republic of

	NCT Number	Title	Other Names	Status	Conditions	Interventions	Characteristics	Population	Sponsor/ Collaborators	Funder Type	Dates	Locations
68	NCT05241405	Evaluation of the Impact of Taking American Ginseng for 8 Weeks on Fatigue in Patients Treated for Localized Breast Cancer<br><br>Study Documents:	Title Acronym: QISEIN<br><br>Other Ids: 2021-A01550-41	Recruiting	• Breast Cancer<br>• Fatigue	• Dietary Supplement: QISENG<br>• Dietary Supplement: PLACEBO	Study Type: Interventional<br><br>Phase: Not Applicable<br><br>Study Design:<br>• Allocation: Randomized<br>• Intervention Model: Parallel Assignment<br>• Masking: Double (Participant, Investigator)<br>• Primary Purpose: Treatment<br><br>Outcome Measures:<br>• Fatigue score change [min :5; max:20]<br>• Other dimensions of fatigue [min :12; max:48]<br>• The incidence of treatment-related adverse events<br>• Quality of life level assessed by EORTC QLQ-C30 questionnaire<br>• anxiety level<br>• Cognitive function<br>• Physical activity level<br>• Sleep quality<br>• The level of acceptability of the treatment,	Enrollment: 354<br><br>Age: 18 Years and older (Adult, Older Adult)<br><br>Sex: Female	• Centre Francois Baclesse<br>• NATSUCA laboratory<br>• Groupement Interrégional de Recherche Clinique et d'Innovation	• Other	Study Start: September 27, 2022<br><br>Primary Completion: September 2025<br><br>Study Completion: September 2025<br><br>First Posted: February 15, 2022<br><br>Results First Posted: No Results Posted<br><br>Last Update Posted: September 29, 2022	• ARCOCEA_Clinique Europe, Amiens, France<br>• Centre Pierre Curie, Beuvry, France<br>• Centre François Baclesse, Caen, France<br>• Polyclinique du Parc, Caen, France<br>• Ch Calais, Calais, France<br>• Ch Cherbourg, Cherbourg, France<br>• Clinique de Flandre, Coudekerque-Branche, France<br>• Centre Henri Becquerel, Rouen, France<br>• Clinique des Dentellières, Valenciennes, France

	NCT Number	Title	Other Names	Status	Conditions	Interventions	Characteristics	Population	Sponsor/ Collaborators	Funder Type	Dates	Locations
69	NCT02326766	Blood Pressure Lowering Effect of Supplementation With Korea Red Ginseng Associated With Reductions in Circulating Lp-PLA2 Activity and Lysophospatidylcholines and an Increase in Dihydrobiopterin in Prehypertensive Subjects<br>Study Documents:	Title Acronym:<br>Other Ids: KGC_metabolites_1	Completed	• Prehypertension	• Dietary Supplement: KRG<br>• Dietary Supplement: Placebo	Study Type: Interventional<br>Phase: Phase 3<br>Study Design:<br>• Allocation: Randomized<br>• Intervention Model: Parallel Assignment<br>• Masking: Quadruple (Participant, Care Provider, Investigator, Outcomes Assessor)<br>• Primary Purpose: Prevention<br>Outcome Measures:<br>• changes from baselilne in blood pressure<br>• changes from baseline in plasma metabolites	Enrollment: 62<br>Age: 20 Years to 70 Years (Adult, Older Adult)<br>Sex: All	• Yonsei University	• Other	Study Start: November 2011<br>Primary Completion: February 2012<br>Study Completion: March 2012<br>First Posted: December 29, 2014<br>Results First Posted: No Results Posted<br>Last Update Posted: December 29, 2014	
70	NCT02392819	Effects on Metabolism and Cognitive Functions of a Commonly Used Commercial Food Supplement<br>Study Documents:	Title Acronym:<br>Other Ids: LundU	Completed	• Postprandial Blood Glucose Regulation	• Dietary Supplement: Panax ginseng<br>• Dietary Supplement: Placebo	Study Type: Interventional<br>Phase: Not Applicable<br>Study Design:<br>• Allocation: Randomized<br>• Intervention Model: Crossover Assignment<br>• Masking: Single (Participant)<br>• Primary Purpose: Prevention<br>Outcome Measures:<br>• blood glucose<br>• serum insulin<br>• Mood as measured by ratings on 100 mm visual analog scales(VAS rating scales)	Enrollment: 22<br>Age: 40 Years to 60 Years (Adult)<br>Sex: All	• Lund University<br>• Anti-Diabetic Food Centre	• Other	Study Start: January 2015<br>Primary Completion: June 2015<br>Study Completion: July 2015<br>First Posted: March 19, 2015<br>Results First Posted: No Results Posted<br>Last Update Posted: September 3, 2015	• Food for Health Science Centre, Medicon Village, Lund, Sweden

	NCT Number	Title	Other Names	Status	Conditions	Interventions	Characteristics	Population	Sponsor/ Collaborators	Funder Type	Dates	Locations
71	NCT03679364	An Observational Registry Study of LUOTAI in Patients With Acute Ischemic Stroke in Vietnam<br><br>Study Documents:	Title Acronym:<br><br>Other Ids: KPC.VS.01	Completed	•Infarction, Anterior Cerebral Artery<br>•Infarction, Middle Cerebral Artery<br>•Ischemic Attack, Transient		Study Type: Observational<br><br>Phase:<br><br>Study Design:<br>•Observational Model: Case-Control<br>•Time Perspective: Prospective<br><br>Outcome Measures:<br>•Primary Efficacy Endpoint: Modified Ranking Scale<br>•NIHSS<br>•Cognitive status (MoCA)<br>•mRC Proportion<br>•ARAT Score	Enrollment: 364<br><br>Age: Child, Adult, Older Adult<br><br>Sex: All	•KPC Pharmaceuticals, Inc	•Industry	Study Start: July 2, 2019<br><br>Primary Completion: August 15, 2020<br><br>Study Completion: August 20, 2020<br><br>First Posted: September 20, 2018<br><br>Results First Posted: No Results Posted<br><br>Last Update Posted: September 2, 2020	•Bach Mai Hospital, Hà N#i, Ha# N#i, Vietnam<br>•Phu Tho General Hospital, T#nh Phú, Phu Tho, Vietnam<br>•103 Military Hospital, Hanoi, Vietnam
72	NCT02553382	Fibre Grain Herb Trial in Type 2 Diabetes<br><br>Study Documents:	Title Acronym: FIGHT<br><br>Other Ids: 15-111	Completed	•Type 2 Diabetes	•Dietary Supplement: Dietary, Herbal<br>•Dietary Supplement: Positive Control	Study Type: Interventional<br><br>Phase: Not Applicable<br><br>Study Design:<br>•Allocation: Randomized<br>•Intervention Model: Parallel Assignment<br>•Masking: Double (Participant, Investigator)<br>•Primary Purpose: Treatment<br><br>Outcome Measures:<br>•Change in HbA1c<br>•Change in LDL-C<br>•Change in 24h ambulatory systolic blood pressure	Enrollment: 104<br><br>Age: 40 Years to 75 Years (Adult, Older Adult)<br><br>Sex: All	•Unity Health Toronto<br>•Canadian Diabetes Association	•Other	Study Start: November 2015<br><br>Primary Completion: April 2018<br><br>Study Completion: April 2018<br><br>First Posted: September 17, 2015<br><br>Results First Posted: No Results Posted<br><br>Last Update Posted: May 26, 2020	•St. Michael's Hopsital, Toronto, Ontario, Canada<br>•Clinical Centre Vuk Vrhovac, Merkur Hospital, Zagreb, Croatia

	NCT Number	Title	Other Names	Status	Conditions	Interventions	Characteristics	Population	Sponsor/ Collaborators	Funder Type	Dates	Locations
73	NCT05228873	Comparison of Quality of Life After Discharge of the Mild and Moderate COVID-19 Patients With or Without Herbal Medicine<br>Study Documents:	Title Acronym:<br>Other Ids: 838-9/H###-#HYD	Recruiting	•COVID-19 Pandemic<br>•Quality of Life	•Drug: Shen Cao Gan Jiang Tang (Gan Cao Gan Jiang Tang with Ginseng)	Study Type: Observational<br>Phase:<br>Study Design:<br>•Observational Model: Case-Control<br>•Time Perspective: Cross-Sectional<br>Outcome Measures:<br>•Quality of Life (QoL) questionnaire<br>•Post COVID-19 symptoms	Enrollment: 300<br>Age: 18 Years to 64 Years (Adult)<br>Sex: All	•University of Medicine and Pharmacy at Ho Chi Minh City	•Other	Study Start: December 20, 2021<br>Primary Completion: March 21, 2022<br>Study Completion: July 15, 2022<br>First Posted: February 8, 2022<br>Results First Posted: No Results Posted<br>Last Update Posted: February 23, 2022	•University of Medicine and Pharmacy at Ho Chi Minh City, Ho Chi Minh, Vietnam
74	NCT05117385	The Effect of the Consumption of 4 Botanical Extracts on Immunity in Healthy Adults (B-4-Immune)<br>Study Documents:	Title Acronym: B-4-Immune<br>Other Ids: 2021-A01973-38	Recruiting	•Healthy Subject<br>•Immunity	•Dietary Supplement: Botanical extract	Study Type: Interventional<br>Phase: Not Applicable<br>Study Design:<br>•Allocation: Randomized<br>•Intervention Model: Parallel Assignment<br>•Masking: Quadruple (Participant, Care Provider, Investigator, Outcomes Assessor)<br>•Primary Purpose: Prevention<br>Outcome Measures:<br>•Ex vivo Immune blood cell response after challenges at baseline (before supplementation) and after 28 days of each botanical consumption compared to placebo<br>•1. Frequency of innate and adaptative immune cell before and after 28 days of consumption of each botanical extract compared to placebo<br>•2. Variation of quality of life before and after 28 days of consumption of each botanical extract compared to placebo	Enrollment: 115<br>Age: 40 Years to 60 Years (Adult)<br>Sex: All	•Naturex SA<br>•Biofortis Mérieux NutriSciences	•Industry<br>•Other	Study Start: November 22, 2021<br>Primary Completion: July 18, 2022<br>Study Completion: July 18, 2022<br>First Posted: November 11, 2021<br>Results First Posted: No Results Posted<br>Last Update Posted: November 30, 2021	•Biofortis Merieux Nutrisciences, Saint-Herblain, France

	NCT Number	Title	Other Names	Status	Conditions	Interventions	Characteristics	Population	Sponsor/ Collaborators	Funder Type	Dates	Locations
75	NCT01155076	Effects of a Vitality Dietary Supplement on Physical and Mental Function in Middle-aged Adults<br>Study Documents:	Title Acronym:<br>Other Ids: 10-SUS-03-NU-01	Completed	•Health-related Quality of Life	•Dietary Supplement: Vitality Product<br>•Dietary Supplement: Placebo	Study Type: Interventional<br>Phase: Not Applicable<br>Study Design:<br>•Allocation: Randomized<br>•Intervention Model: Parallel Assignment<br>•Masking: Quadruple (Participant, Care Provider, Investigator, Outcomes Assessor)<br>•Primary Purpose: Supportive Care<br>Outcome Measures:<br>•Health-related quality of life<br>•Medical Outcomes Study (MOS) Sexual Function questionnaire<br>•Adverse events	Enrollment: 116<br>Age: 40 Years to 70 Years (Adult, Older Adult)<br>Sex: All	•Pharmanex<br>•Sprim Advanced Life Sciences	•Industry<br>•Other	Study Start: July 2010<br>Primary Completion: November 2012<br>Study Completion: November 2013<br>First Posted: July 1, 2010<br>Results First Posted: No Results Posted<br>Last Update Posted: November 14, 2013	•Quality of Life Medical and Research Center, Tucson, Arizona, United States<br>•Ridgeview Chaska Medical Plaza, Chaska, Minnesota, United States<br>•West Houston Clinical Research Services, Houston, Texas, United States
76	NCT05129917	Effects of Supplementation With Ginseng and BCAA Improved Central Fatigue and Enhanced Attention During Exercise<br>Study Documents:	Title Acronym:<br>Other Ids: TSMH No.13-023-A2	Completed	•Exercise Addiction	•Dietary Supplement: Placebo<br>•Dietary Supplement: Gojinsen® drinks	Study Type: Interventional<br>Phase: Not Applicable<br>Study Design:<br>•Allocation: Randomized<br>•Intervention Model: Parallel Assignment<br>•Masking: Double (Participant, Investigator)<br>•Primary Purpose: Treatment<br>Outcome Measures:<br>•Endurance exercise performance testing<br>•rating of perceived exertion<br>•blood lactate<br>•Continuous attention performance test	Enrollment: 16<br>Age: 20 Years to 25 Years (Adult)<br>Sex: All	•TCI Co., Ltd.	•Industry	Study Start: June 1, 2013<br>Primary Completion: December 1, 2013<br>Study Completion: December 31, 2013<br>First Posted: November 22, 2021<br>Results First Posted: No Results Posted<br>Last Update Posted: December 6, 2021	

	NCT Number	Title	Other Names	Status	Conditions	Interventions	Characteristics	Population	Sponsor/ Collaborators	Funder Type	Dates	Locations
77	NCT03911921	RSYR for Fatigue Reduction in Cancer Survivors<br>Study Documents:	Title Acronym:<br>Other Ids:<br>•FATIGUE<br>•PZ2017019	Completed	•Cancer-related Problem/Condition	•Drug: RSYRT decoction<br>•Drug: Astragalus membranaceus	Study Type: Interventional<br>Phase: Phase 2<br>Study Design:<br>•Allocation: Randomized<br>•Intervention Model: Parallel Assignment<br>•Masking: Double (Participant, Investigator)<br>•Primary Purpose: Treatment<br>Outcome Measures: Fatigue score	Enrollment: 92<br>Age: 18 Years to 65 Years (Adult, Older Adult)<br>Sex: All	•Peking University Cancer Hospital & Institute	•Other	Study Start: June 1, 2015<br>Primary Completion: February 1, 2019<br>Study Completion: February 1, 2019<br>First Posted: April 11, 2019<br>Results First Posted: No Results Posted<br>Last Update Posted: April 16, 2019	
78	NCT02603016	Efficacy Study of Clinical Nutrition to Treat Lung Neoplasms And Breast Carcinoma<br>Study Documents:	Title Acronym:<br>Other Ids: PLAGHS201503601	Completed	•Lung Neoplasms<br>•Breast Neoplasms	•Drug: GLSE compound<br>•Drug: Maitake mushroom extract compound<br>•Drug: Ginseng compound	Study Type: Interventional<br>Phase: Not Applicable<br>Study Design:<br>•Allocation: Randomized<br>•Intervention Model: Parallel Assignment<br>•Masking: Single (Participant)<br>•Primary Purpose: Supportive Care<br>Outcome Measures: T-lymphocyte cell subsets	Enrollment: 480<br>Age: 18 Years to 70 Years (Adult, Older Adult)<br>Sex: All	•Chinese PLA General Hospital	•Other	Study Start: November 2015<br>Primary Completion: November 2016<br>Study Completion: February 2017<br>First Posted: November 11, 2015<br>Results First Posted: No Results Posted<br>Last Update Posted: March 14, 2017	•PLA general hospital, Beijing, Beijing, China
79	NCT01636154	Clinical Application and Basic Study of Medicinal Fractions-based Herbal Combinations<br>Study Documents:	Title Acronym:<br>Other Ids: BUCMDFH9634	Completed	•Ischemic Stroke	•Drug: Ixeris of sonchifolia Hance<br>•Drug: Panax notoginseng saponins<br>•Drug: Ixeris of sonchifolia Hance combined with Panax notoginseng saponins	Study Type: Interventional<br>Phase: Not Applicable<br>Study Design:<br>•Allocation: Randomized<br>•Intervention Model: Factorial Assignment<br>•Masking: None (Open Label)<br>•Primary Purpose: Treatment<br>Outcome Measures:	Enrollment: 120<br>Age: 40 Years to 75 Years (Adult, Older Adult)<br>Sex: All	•Yunling Zhang<br>•Shandong University of Traditional Chinese Medicine<br>•Huairou Hospital of Traditional Chinese Medicine<br>•Dongfang Hospital Beijing University of Chinese Medicine	•Other	Study Start: January 2012<br>Primary Completion: March 2016<br>Study Completion: August 2016<br>First Posted: July 10, 2012<br>Results First Posted: No Results Posted<br>Last Update Posted: August 5, 2016	•Department of Neurology,Dongfang Hospital,Beijing University of Chinese Medicine, Beijing, Beijing, China<br>•Department of Neurology,Huairou Hospital of Traditional Chinese Medicine, Beijing, Beijing, China<br>•Department of Neurology,Affiliated Hospital of Shandong University of Traditional Chinese Medicine, Jinan, Shandong, China

	NCT Number	Title	Other Names	Status	Conditions	Interventions	Characteristics	Population	Sponsor/ Collaborators	Funder Type	Dates	Locations
80	NCT01761227	Efficacy and Safety of Fufangdanshen Tablets in Mild to Moderate Vascular Dementia<br><br>Study Documents:	Title Acronym:<br><br>Other Ids:<br>• SFDA2005L01916<br>• SFDA#2008#I919	Completed	• Vascular Dementia	• Drug: Fufangdanshen Tablets<br>• Drug: Placebo	Study Type: Interventional<br><br>Phase: Phase 2<br><br>Study Design:<br>• Allocation: Randomized<br>• Intervention Model: Parallel Assignment<br>• Masking: Quadruple (Participant, Care Provider, Investigator, Outcomes Assessor)<br>• Primary Purpose: Treatment<br><br>Outcome Measures:<br>• Change from baseline to end of double-blind treatment Clinician's Interview Based Impression of Change - Plus Caregiver Input; Alzheimer Disease Assessment Scale-cognitive subscale<br>• Change in cognitive scores: Alzheimer Disease Assessment Scale-cognitive. subscale (ADAS-cog)<br>• Change in functional scores: Activities of Daily Living (ADL).<br>• Change from baseline to the end of double-blind treatment in Mini-mental state examination (MMSE) scores	Enrollment: 240<br><br>Age: 45 Years to 80 Years (Adult, Older Adult)<br><br>Sex: All	• Dongzhimen Hospital, Beijing<br>• Hutchison Whampoa Guangzhou Baiyunshan Chinese Medicine Company Limited	• Other<br>• Industry	Study Start: September 2012<br><br>Primary Completion: December 2015<br><br>Study Completion: December 2015<br><br>First Posted: January 4, 2013<br><br>Results First Posted: No Results Posted<br><br>Last Update Posted: October 23, 2018	• Dongzhimen Hospital ,Beijing University of Chinese Medicine, Beijing, Beijing, China

	NCT Number	Title	Other Names	Status	Conditions	Interventions	Characteristics	Population	Sponsor/ Collaborators	Funder Type	Dates	Locations
81	NCT02482324	Phase 1 Study of ALZT-OP1 Combination Therapy in Normal Healthy Volunteers<br><br>Study Documents:	Title Acronym:<br><br>Other Ids:<br>AZT-002	Completed	• Healthy Volunteers	• Drug: ALZT-OP1a<br>• Drug: ALZT-OP1b<br>• Device: Dry Powder Inhaler	Study Type:<br>Interventional<br><br>Phase:<br>Phase 1<br><br>Study Design:<br>• Allocation: Randomized<br>• Intervention Model: Crossover Assignment<br>• Masking: None (Open Label)<br><br>Outcome Measures:<br>• Non-compartmental plasma pharmacokinetics for ALZT-OP1a and ALZT-OP1b<br>• Levels of ALZT-OP1a and ALZT-OP1b in cerebrospinal fluid (CSF)<br>• Number of Treatment Emergent Adverse Events (TEAE)	Enrollment:<br>26<br><br>Age:<br>55 Years to 75 Years (Adult, Older Adult)<br><br>Sex:<br>All	• AZTherapies, Inc.<br>• Panax Clinical Research<br>• Pharma Consulting Group AB<br>• KCAS	• Industry<br>• Other	Study Start:<br>June 2015<br><br>Primary Completion:<br>July 2015<br><br>Study Completion:<br>July 2015<br><br>First Posted:<br>June 26, 2015<br><br>Results First Posted:<br>No Results Posted<br><br>Last Update Posted:<br>July 21, 2015	• Panax Clinical Research, Miami Lakes, Florida, United States
82	NCT00965822	A Randomized, Double-blind, Placebo-controlled Study on the Efficacy and Safety of CVT-E002 in the Treatment of Upper Respiratory Tract Infections in a Pediatric (3-11 Years) Population<br><br>Study Documents:	Title Acronym:<br><br>Other Ids:<br>CVT-E002-2007-3	Completed	• Upper Respiratory Tract Infections	• Other: North American ginseng<br>• Other: Placebo	Study Type:<br>Interventional<br><br>Phase:<br>Phase 2<br><br>Study Design:<br>• Allocation: Randomized<br>• Intervention Model: Parallel Assignment<br>• Masking: Quadruple (Participant, Care Provider, Investigator, Outcomes Assessor)<br>• Primary Purpose: Treatment<br><br>Outcome Measures:<br>• The primary objective is to assess the efficacy of acute dosing of CVT-E002 in reducing the duration of URTI symptoms in children 3-11 years of age<br>• To asses the efficacy of acute dosing of CVT-E002 in reducing: (1) symptom severity; (2) peak CARIFS scores and; (3) absenteeism for participant and/or parent/guardian.	Enrollment:<br>293<br><br>Age:<br>3 Years to 11 Years (Child)<br><br>Sex:<br>All	• Afexa Life Sciences Inc	• Industry	Study Start:<br>September 2009<br><br>Primary Completion:<br>April 2012<br><br>Study Completion:<br>April 2012<br><br>First Posted:<br>August 26, 2009<br><br>Results First Posted:<br>No Results Posted<br><br>Last Update Posted:<br>August 31, 2012	• Alberta Health Services, Edmonton, Alberta, Canada<br>• Saint John Regional Hospital, Saint John, New Brunswick, Canada<br>• Canadian Center for Vaccinology, Dalhousie University, Halifax, Nova Scotia, Canada<br>• JDM Research, Toronto, Ontario, Canada

	NCT Number	Title	Other Names	Status	Conditions	Interventions	Characteristics	Population	Sponsor/ Collaborators	Funder Type	Dates	Locations
83	NCT03028077	Effects of GS-3K8 and GINst15 on Acute Respiratory Illness<br>Study Documents:	Title Acronym:<br>Other Ids: CTCF2_2014_GN	Completed	• Acute Respiratory Infection	• Dietary Supplement: GS-3K8<br>• Dietary Supplement: GINst15<br>• Dietary Supplement: Placebo	Study Type: Interventional<br>Phase: Not Applicable<br>Study Design:<br>• Allocation: Randomized<br>• Intervention Model: Parallel Assignment<br>• Masking: Double (Participant, Investigator)<br>• Primary Purpose: Prevention<br>Outcome Measures:<br>• Incidence rate of ARI (by questionnaire)<br>• Development of ARI symptoms (by questionnaire)<br>• Duration of ARI symptoms (by questionnaire)	Enrollment: 45<br>Age: 39 Years to 65 Years (Adult, Older Adult)<br>Sex: All	• Chonbuk National University Hospital	• Other	Study Start: October 2014<br>Primary Completion: March 2015<br>Study Completion: March 2015<br>First Posted: January 23, 2017<br>Results First Posted: No Results Posted<br>Last Update Posted: January 23, 2017	• Clinical Trial Center for Functional Foods Chonbuk National University Hospital, Jeonju, Jeollabuk-do, Korea, Republic of
84	NCT03681249	the Integrated Traditional Chinese and Western Medicine Treat Middle Stage DKD<br>Study Documents:	Title Acronym:<br>Other Ids: 17401970500-b	Completed	• Diabetic Nephropathy Type 2	• Drug: ShenqiDihuang Decoction<br>• Drug: ShenqiDihuang Decoction placebo	Study Type: Interventional<br>Phase: Early Phase 1<br>Study Design:<br>• Allocation: Randomized<br>• Intervention Model: Parallel Assignment<br>• Masking: Double (Participant, Investigator)<br>• Primary Purpose: Treatment<br>Outcome Measures:<br>24H urine total protein	Enrollment: 112<br>Age: 18 Years to 75 Years (Adult, Older Adult)<br>Sex: All	• Shanghai University of Traditional Chinese Medicine<br>• Shanghai Jiao Tong University Affiliated Sixth People's Hospital	• Other	Study Start: November 2, 2018<br>Primary Completion: December 31, 2021<br>Study Completion: December 31, 2021<br>First Posted: September 24, 2018<br>Results First Posted: No Results Posted<br>Last Update Posted: May 6, 2022	• Longhua Hospital, Shanghai University of Traditional Chinese Medicine, Shanghai, Shanghai, China

	NCT Number	Title	Other Names	Status	Conditions	Interventions	Characteristics	Population	Sponsor/ Collaborators	Funder Type	Dates	Locations
85	NCT01622101	Thermogenic Properties of Zantrex-3<br><br>Study Documents:	Title Acronym: ZANTREX<br><br>Other Ids: B238	Completed	• Obesity	• Dietary Supplement: Thermogenic properties of Zantrex-3®	Study Type: Interventional<br><br>Phase: Not Applicable<br><br>Study Design:<br>• Allocation: Randomized<br>• Intervention Model: Crossover Assignment<br>• Masking: Quadruple (Participant, Care Provider, Investigator, Outcomes Assessor)<br>• Primary Purpose: Prevention<br><br>Outcome Measures:<br>• Acute 3-h changes from baseline in energy expenditure and respiratory quotient (RQ)<br>• 3-h acute change in blood pressure (systolic and diastolic)<br>• 3-h acute change in heart rate<br>• Acute 3-h changes from baseline in subjective appetite sensations using visual analogue scales<br>• Acute 3-h changes from baseline in self-reported discomfort	Enrollment: 23<br><br>Age: 18 Years to 50 Years (Adult)<br><br>Sex: Male	• University of Copenhagen	• Other	Study Start: May 2007<br><br>Primary Completion: August 2007<br><br>Study Completion: August 2007<br><br>First Posted: June 18, 2012<br><br>Results First Posted: No Results Posted<br><br>Last Update Posted: June 21, 2012	• Department of Human Nutrition, Faculty of Science, University of Copenhagen, Denmark, Frederiksberg, Denmark

	NCT Number	Title	Other Names	Status	Conditions	Interventions	Characteristics	Population	Sponsor/ Collaborators	Funder Type	Dates	Locations
86	NCT03388762	RCT of a Polyherbal Dietary Supplement for Prediabetes<br><br>Study Documents:	Title Acronym:<br><br>Other Ids:<br>022-DFH, HP-00075768	Completed	• Prediabetic State	• Dietary Supplement: GlucoSupreme™ Herbal<br>• Other: Placebo	Study Type:<br>Interventional<br><br>Phase:<br>Not Applicable<br><br>Study Design:<br>• Allocation: Randomized<br>• Intervention Model: Parallel Assignment<br>• Masking: Double (Participant, Care Provider)<br>• Primary Purpose: Treatment<br><br>Outcome Measures:<br>• Fasting Blood Glucose<br>• Glycated hemoglobin/A1C (HbA1c)<br>• Fasting insulin<br>• Insulin Resistance (HOMA-IR)<br>• #-cell function (HOMA-#)<br>• Quantitative Insulin Sensitivity Check Index (QUICKI)<br>• Fructosamine<br>• GlycoMark<br>• Lipid profile<br>• Inflammation<br>• Supplement compliance assessed using participant daily diary<br>• Adverse events	Enrollment:<br>39<br><br>Age:<br>18 Years and older (Adult, Older Adult)<br><br>Sex:<br>All	• University of Maryland, Baltimore<br>• Alliance Institute for Integrative Medicine	• Other	Study Start:<br>December 5, 2017<br><br>Primary Completion:<br>September 30, 2019<br><br>Study Completion:<br>January 1, 2020<br><br>First Posted:<br>January 3, 2018<br><br>Results First Posted:<br>No Results Posted<br><br>Last Update Posted:<br>June 28, 2022	• University of Maryland Center for Diabetes and Endocrinology, Baltimore, Maryland, United States<br>• University of Maryland Family Medicine Associates, Baltimore, Maryland, United States<br>• University of Maryland School of Medicine, Department of Family and Community Medicine, East Hall, Baltimore, Maryland, United States<br>• Alliance Integrative Medicine, Cincinnati, Ohio, United States
87	NCT00010634	Complementary Naturopathic Medicine for Periodontitis<br><br>Study Documents:	Title Acronym:<br><br>Other Ids:<br>• P50AT000076-01P<br>• P50AT000076-01	Completed	• Periodontitis	• Drug: Connective Tissue Nutrient Formula<br>• Drug: Adaptogenic herbs<br>• Drug: glutamine	Study Type:<br>Interventional<br><br>Phase:<br>Phase 2<br><br>Study Design:<br>• Allocation: Randomized<br>• Primary Purpose: Treatment<br><br>Outcome Measures:	Enrollment:<br><br>Age:<br>35 Years and older (Adult, Older Adult)<br><br>Sex:<br>All	• National Center for Complementary and Integrative Health (NCCIH)	• NIH	Study Start:<br>September 1999<br><br>Primary Completion:<br><br>Study Completion:<br>July 2004<br><br>First Posted:<br>February 5, 2001<br><br>Results First Posted:<br>No Results Posted<br><br>Last Update Posted:<br>August 18, 2006	• The Oregon Health Sciences University (OHSU), Portland, Oregon, United States

	NCT Number	Title	Other Names	Status	Conditions	Interventions	Characteristics	Population	Sponsor/ Collaborators	Funder Type	Dates	Locations
88	NCT03640884	Post-marketing Safety Surveillance of Xueshuantong-Injection : a Registry Study<br><br>Study Documents:	Title Acronym:<br><br>Other Ids:<br>Xueshuantong--V2.0	Recruiting	•Adverse Drug Event<br>•Adverse Drug Reactions<br>•Anaphylactic Reaction<br>•Severe Adverse Reactions	•Drug: Xueshuantong-Injection	Study Type:<br>Observational<br><br>Phase:<br><br>Study Design:<br>•Observational Model: Cohort<br>•Time Perspective: Prospective<br><br>Outcome Measures:<br>•The incidence of severe adverse reactions (SAR) of Xueshuantong-Injection<br>•The incidence of adverse drug reactions (ADR) of Xueshuantong-Injection<br>•The incidence of adverse events #AE#of Xueshuantong-Injection<br>•The incidence of serious adverse events #SAE#of Xueshuantong-Injection<br>•The incidence of anaphylactic reaction of Xueshuantong-Injection<br>•The incidence of new ADRs of Xueshuantong-Injection<br>•The effective rate of Xueshuantong-Injection	Enrollment:<br>30000<br><br>Age:<br>Child, Adult, Older Adult<br><br>Sex:<br>All	•Zhong Wang<br>•China Academy of Chinese Medical Sciences	•Other	Study Start:<br>February 18, 2019<br><br>Primary Completion:<br>December 2021<br><br>Study Completion:<br>June 2022<br><br>First Posted:<br>August 21, 2018<br><br>Results First Posted:<br>No Results Posted<br><br>Last Update Posted:<br>July 7, 2021	•Jizhou City Hospital, Hengshui, Hebei, China<br>•Tongbai County People's Hospital, Nanyang, Henan, China<br>•Lingbao 3th People's Hospital, Sanmenxia, Henan, China<br>•People's Hospital of Dancheng County, Zhoukou, Henan, China<br>•Dongxihu District People's Hospital, Wuhan, Hubei, China<br>•Donghai Country People's Hospital, Lianyungang, Jiangsu, China<br>•People's Hospital of Yichun City, Yichun, Jiangxi, China<br>•Central Hospital of Wafangdian City, Dalian, Liaoning, China<br>•Lingyuan City Central Hospital, Lingyuan, Liaoning, China<br>•Chinse Medical Hospital of Yangxin County, Binzhou, Shandong, China<br>•and 4 more

	NCT Number	Title	Other Names	Status	Conditions	Interventions	Characteristics	Population	Sponsor/ Collaborators	Funder Type	Dates	Locations
89	NCT04849650	PK Study of IV and Oral Amisulpride in Subjects With Severe Renal Impairment<br><br>Study Documents:	Title Acronym:<br><br>Other Ids: DP10026	Completed	• Renal Disease, End Stage	• Drug: Amisulpride IV<br>• Drug: Amisulpride Oral Tablet	Study Type: Interventional<br><br>Phase: Phase 1<br><br>Study Design:<br>• Allocation: Non-Randomized<br>• Intervention Model: Single Group Assignment<br>• Masking: None (Open Label)<br>• Primary Purpose: Basic Science<br><br>Outcome Measures:<br>• PK: Amisulpride plasma exposure (AUC) after a single IV dose<br>• PK: Amisulpride plasma exposure (AUC) after a single oral dose<br>• PK: Cmax<br>• PK: Tmax<br>• PK: T1/2<br>• PK: Clearance<br>• PK: Vd<br>• Safety: AE	Enrollment: 12<br><br>Age: 18 Years to 75 Years (Adult, Older Adult)<br><br>Sex: All	• Acacia Pharma Ltd	• Industry	Study Start: June 3, 2021<br><br>Primary Completion: October 30, 2021<br><br>Study Completion: October 30, 2021<br><br>First Posted: April 19, 2021<br><br>Results First Posted: No Results Posted<br><br>Last Update Posted: December 13, 2021	• Panax Clinical Research, Miami Lakes, Florida, United States

	NCT Number	Title	Other Names	Status	Conditions	Interventions	Characteristics	Population	Sponsor/ Collaborators	Funder Type	Dates	Locations
90	NCT04570644	Randomized I/II Phase Study of ALZT-OP1 Combination Therapy in Alzheimer's Disease and Normal Healthy Volunteers<br>Study Documents:	Title Acronym:<br>Other Ids: AZT-008	Completed	•Healthy Volunteers<br>•Alzheimer Disease	•Drug: ALZT-OP1 (cromolyn and ibuprofen) ALZT-OP1a (cromolyn) and ALZT-OP1b (ibuprofen)	Study Type: Interventional<br>Phase: Phase 1<br>Study Design:<br>•Allocation: Randomized<br>•Intervention Model: Crossover Assignment<br>•Masking: None (Open Label)<br>•Primary Purpose: Treatment<br>Outcome Measures:<br>•Part A Non-compartmental PK parameters will be calculated and reported for ALZT-OP1a and ALZT-OP1b<br>•PK profile for ALZT-OP1a and ALZT-OP1b in plasma and CSF AUC 0-#<br>•PK profile for ALZT-OP1a and ALZT-OP1b in plasma and CSF AUC 0-t<br>•PK profile for ALZT-OP1a and ALZT-OP1b in plasma and CSF AUCPLASMA/ AUCCSF<br>•PK profile for ALZT-OP1a and ALZT-OP1b in plasma and CSF CL/F<br>•PK profile for ALZT-OP1a and ALZT-OP1b in plasma and CSF Cmax<br>•PK profile for ALZT-OP1a and ALZT-OP1b in plasma and CSF t½ (half-life)<br>•PK profile for ALZT-OP1a and ALZT-OP1b in plasma and CSF tmax<br>•PK profile for ALZT-OP1a and ALZT-OP1b in plasma and CSF Vd/F<br>•Biomarker Beta Amyloid (##-42) Sample Analysis plasma and CSF Day 1 to 60 Days<br>•and 12 more	Enrollment: 56<br>Age: 55 Years to 79 Years (Adult, Older Adult)<br>Sex: All	•AZTherapies, Inc.	•Industry	Study Start: August 28, 2020<br>Primary Completion: January 18, 2021<br>Study Completion: January 18, 2021<br>First Posted: September 30, 2020<br>Results First Posted: No Results Posted<br>Last Update Posted: March 3, 2022	•Panax Clinical Research, Miami Lakes, Florida, United States

	NCT Number	Title	Other Names	Status	Conditions	Interventions	Characteristics	Population	Sponsor/ Collaborators	Funder Type	Dates	Locations
91	NCT05323136	Evaluate the Effects of Renal Impairment on the Pharmacokinetics and Pharmacodynamics<br><br>Study Documents:	Title Acronym:<br><br>Other Ids:<br>MT1002-I-C02	Recruiting	•ACS - Acute Coronary Syndrome	•Drug: MT1002 for Injection	Study Type:<br>Interventional<br><br>Phase:<br>Phase 1<br><br>Study Design:<br>•Allocation: Randomized<br>•Intervention Model: Sequential Assignment<br>•Masking: None (Open Label)<br>•Primary Purpose: Treatment<br><br>Outcome Measures:<br>•Plasma PK<br>•plasma PK<br>•Urine PK<br>•Adverse events	Enrollment:<br>24<br><br>Age:<br>18 Years to 80 Years (Adult, Older Adult)<br><br>Sex:<br>All	•Shaanxi Micot Technology Limited Company	•Industry	Study Start:<br>April 15, 2022<br><br>Primary Completion:<br>March 31, 2023<br><br>Study Completion:<br>April 30, 2023<br><br>First Posted:<br>April 12, 2022<br><br>Results First Posted:<br>No Results Posted<br><br>Last Update Posted:<br>October 25, 2022	•Panax Clinical Research, LLC., Miami, Florida, United States
92	NCT02232893	Effect of TU-100 in Patients Undergoing Laparoscopic Colectomy<br><br>Study Documents:<br>•Study Protocol and Statistical Analysis Plan	Title Acronym:<br>TU100P2T3<br><br>Other Ids:<br>TU100P2T3	Completed	•Postoperative Ileus	•Drug: Daikenchuto (TU-100)<br>•Drug: Placebo	Study Type:<br>Interventional<br><br>Phase:<br>Phase 2<br><br>Study Design:<br>•Allocation: Randomized<br>•Intervention Model: Parallel Assignment<br>•Masking: Quadruple (Participant, Care Provider, Investigator, Outcomes Assessor)<br>•Primary Purpose: Treatment<br><br>Outcome Measures:<br>Quality of Life After Surgery Based on Gastrointestinal Quality of Life Index (GIQLI) Global Score From Baseline to Visit 4.	Enrollment:<br>69<br><br>Age:<br>18 Years and older (Adult, Older Adult)<br><br>Sex:<br>All	•Tsumura USA<br>•Cato Research	•Industry	Study Start:<br>September 2014<br><br>Primary Completion:<br>May 2017<br><br>Study Completion:<br>May 2017<br><br>First Posted:<br>September 5, 2014<br><br>Results First Posted:<br>June 19, 2020<br><br>Last Update Posted:<br>June 19, 2020	•Los Angeles Site, Los Angeles, California, United States<br>•Aurora Site, Aurora, Colorado, United States<br>•Weston Site, Weston, Florida, United States<br>•Atlanta Site, Atlanta, Georgia, United States<br>•Chicago Site, Chicago, Illinois, United States<br>•Metairie Site, Metairie, Louisiana, United States<br>•Burlington, MA Site, Burlington, Massachusetts, United States<br>•Coon Rapids Site, Coon Rapids, Minnesota, United States<br>•Minneapolis Site, Minneapolis, Minnesota, United States<br>•Jackson Site, Jackson, Mississippi, United States<br>•and 3 more

	NCT Number	Title	Other Names	Status	Conditions	Interventions	Characteristics	Population	Sponsor/ Collaborators	Funder Type	Dates	Locations
93	NCT02413099	The Efficacy and Safety of New Herbal Formula (KBMSI-2) in the Treatment of Erectile Dysfunction<br><br>Study Documents:	Title Acronym:<br><br>Other Ids:<br>KBMSI-2 Study	Completed	• Erectile Dysfunction	• Drug: KBMSI-2	Study Type:<br>Interventional<br><br>Phase:<br>Phase 4<br><br>Study Design:<br>• Allocation: Randomized<br>• Intervention Model: Parallel Assignment<br>• Masking: Double<br>• Primary Purpose: Treatment<br><br>Outcome Measures:<br>• Change in the EF domain scores of the IIEF questionnaire from baseline<br>• Change in all domain scores of the IIEF from baseline<br>• change in question 2 and 3 of the Sexual Encounter Profile (SEP2: Were you able to insert your penis in your partner's vagina? SEP3: Did your erection last long enough for you to have a successful intercourse?) from baseline	Enrollment:<br>44<br><br>Age:<br>19 Years to 40 Years (Adult)<br><br>Sex:<br>Male	• Hyun Jun Park<br>• Pusan National University Hospital	• Other	Study Start:<br>February 2012<br><br>Primary Completion:<br>November 2012<br><br>Study Completion:<br>January 3, 2013<br><br>First Posted:<br>April 9, 2015<br><br>Results First Posted:<br>No Results Posted<br><br>Last Update Posted:<br>August 29, 2018	• Department of Urology, Pusan National University Hospital, Busan, Korea, Republic of

	NCT Number	Title	Other Names	Status	Conditions	Interventions	Characteristics	Population	Sponsor/ Collaborators	Funder Type	Dates	Locations
94	NCT00726401	A Randomized, Double-blind, Placebo-controlled Study on the Effect of CVT-E002 in Patients With Seasonal Allergic Rhinitis<br><br>Study Documents:	Title Acronym:<br><br>Other Ids: CVT-E002-2007-2	Completed	•Seasonal Allergic Rhinitis	•Drug: COLD-fX<br>•Drug: Placebo	Study Type: Interventional<br><br>Phase: Phase 2<br><br>Study Design:<br>•Allocation: Randomized<br>•Intervention Model: Parallel Assignment<br>•Masking: Quadruple (Participant, Care Provider, Investigator, Outcomes Assessor)<br>•Primary Purpose: Treatment<br><br>Outcome Measures:<br>•Preliminary estimates of treatment effect of CVT-E002 in improving quality of life and reducing symptoms<br>•Safety and tolerability of CVT-E002	Enrollment: 200<br><br>Age: 12 Years to 75 Years (Child, Adult, Older Adult)<br><br>Sex: All	•Afexa Life Sciences Inc	•Industry	Study Start: May 2008<br><br>Primary Completion: September 2010<br><br>Study Completion: September 2010<br><br>First Posted: July 31, 2008<br><br>Results First Posted: No Results Posted<br><br>Last Update Posted: October 11, 2010	•Capital Health, Edmonton, Alberta, Canada<br>•McMaster University Medical Centre, Hamilton, Ontario, Canada<br>•Melimar Allergy Laboratory, Toronto, Ontario, Canada<br>•JDM Research, Toronto, Ontario, Canada

	NCT Number	Title	Other Names	Status	Conditions	Interventions	Characteristics	Population	Sponsor/ Collaborators	Funder Type	Dates	Locations
95	NCT05559372	Energy Drink Effects on Performance, Mood, and Cardiovascular Outcomes<br><br>Study Documents:	Title Acronym:<br><br>Other Ids: 202107364	Completed	• Caffeine<br>• Exercise Performance<br>• Energy Drink<br>• Cardiovascular Safety<br>• Mood	• Dietary Supplement: Acute energy drink or placebo consumption	Study Type: Interventional<br><br>Phase: Not Applicable<br><br>Study Design:<br>• Allocation: Randomized<br>• Intervention Model: Crossover Assignment<br>• Masking: Triple (Participant, Investigator, Outcomes Assessor)<br>• Primary Purpose: Other<br><br>Outcome Measures:<br>• Maximal Exercise Performance<br>• Sub-maximal Exercise Performance<br>• Fatiguing Isometric Exercise Performance<br>• Mood<br>• Change in Systolic Blood Pressure<br>• Change in Diastolic Blood Pressure<br>• Change in Heart Rate<br>• Change in Rate Pressure Product<br>• Change in QTc Interval<br>• Change in Leg Blood Flow	Enrollment: 109<br><br>Age: 20 Years to 35 Years (Adult)<br><br>Sex: Male	• Nathaniel Jenkins<br>• University of Iowa	• Other	Study Start: October 25, 2021<br><br>Primary Completion: March 4, 2022<br><br>Study Completion: March 4, 2022<br><br>First Posted: September 29, 2022<br><br>Results First Posted: No Results Posted<br><br>Last Update Posted: September 29, 2022	• Integrative Laboratory of Applied Physiology and Lifestyle Medicine, Iowa City, Iowa, United States

	NCT Number	Title	Other Names	Status	Conditions	Interventions	Characteristics	Population	Sponsor/ Collaborators	Funder Type	Dates	Locations
96	NCT01598272	Effects of a Proprietary Blend of Herbal Extract Supplement on Cellular Detoxification, Inflammation, and Cumulative Cognitive Index as Well as Gene Expression in Middle-Aged Adult Women<br><br>Study Documents:	Title Acronym: Pharmanex<br><br>Other Ids: 11-PHX-03-NU-01	Completed	•Anti Aging	•Dietary Supplement: Vitality product AM + Vitality product PM<br>•Dietary Supplement: Placebo	Study Type: Interventional<br><br>Phase: Phase 4<br><br>Study Design:<br>•Allocation: Randomized<br>•Intervention Model: Parallel Assignment<br>•Masking: Quadruple (Participant, Care Provider, Investigator, Outcomes Assessor)<br>•Primary Purpose: Supportive Care<br><br>Outcome Measures:<br>•Determine effects of investigational supplement on gene expression profiles.<br>•Establish a safety profile of investigational supplement<br>•Determine effects of investigational supplement on markers of inflammation<br>•Determine effects of investigational supplement on composite cognitive index<br>•Determine changes in scoring on HADS assessment (Hospital Anxiety & Depression Scale)<br>•Determine changes in Quality of Life<br>•Determine changes in sleep patterns and quality<br>•Determine changes to overall health<br>•Determine changes to biological age scanning (Digital Pulse Wave Analyzer (DPA)<br>•Determine changes in Biophotonic Scanner Scores<br>•Determine changes in skin advanced glycation endproducts (AGE) levels	Enrollment: 95<br><br>Age: 35 Years to 73 Years (Adult, Older Adult)<br><br>Sex: Female	•Pharmanex<br>•Aspen Clinical Research	•Industry<br>•Other	Study Start: July 2011<br><br>Primary Completion: June 2012<br><br>Study Completion: June 2012<br><br>First Posted: May 15, 2012<br><br>Results First Posted: No Results Posted<br><br>Last Update Posted: December 11, 2014	•Aspen Clinical Research, Orem, Utah, United States

	NCT Number	Title	Other Names	Status	Conditions	Interventions	Characteristics	Population	Sponsor/ Collaborators	Funder Type	Dates	Locations
97	NCT05092113	The Efficacy and Safety of a Compound Glutamine Capsule in the Primary Prevention of Chemotherapy-induced Mucositis<br><br>Study Documents:	Title Acronym:<br><br>Other Ids: 2021-615	Recruiting	•Mucositis<br>•Chemotherapeutic Toxicity	•Drug: a compound glutamine capsule/ a compound glutamine capsule simulated placebo	Study Type: Interventional<br><br>Phase: Phase 3<br><br>Study Design:<br>•Allocation: Randomized<br>•Intervention Model: Parallel Assignment<br>•Masking: Double (Participant, Investigator)<br>•Primary Purpose: Prevention<br><br>Outcome Measures:<br>•Overall incidence of chemotherapy-induced diarrhea # grade 1<br>•Overall incidence of gastrointestinal adverse events except diarrhea # grade 1<br>•Overall incidence of grade 3/4 gastrointestinal adverse events	Enrollment: 108<br><br>Age: 18 Years to 75 Years (Adult, Older Adult)<br><br>Sex: All	•Meng Qiu<br>•West China Hospital	•Other	Study Start: June 30, 2021<br><br>Primary Completion: December 31, 2022<br><br>Study Completion: December 31, 2022<br><br>First Posted: October 25, 2021<br><br>Results First Posted: No Results Posted<br><br>Last Update Posted: October 25, 2021	•Meng Qiu, Sichuan, China
98	NCT04988971	The Efficacy and Safety of a Compound Glutamine Capsule in the Prevention of Chemotherapy-induced Mucositis<br><br>Study Documents:	Title Acronym:<br><br>Other Ids: 2021615	Recruiting	•Mucositis<br>•Chemotherapeutic Toxicity	•Drug: a compound glutamine capsule<br>•Drug: a compound glutamine capsule simulated placebo	Study Type: Interventional<br><br>Phase: Phase 3<br><br>Study Design:<br>•Allocation: Randomized<br>•Intervention Model: Parallel Assignment<br>•Masking: Double (Participant, Investigator)<br>•Primary Purpose: Prevention<br><br>Outcome Measures:<br>•Overall incidence of chemotherapy-induced diarrhea # grade 1<br>•Overall incidence of gastrointestinal adverse events except diarrhea # grade 1<br>•Overall incidence of grade 3/4 gastrointestinal adverse events	Enrollment: 90<br><br>Age: 18 Years to 75 Years (Adult, Older Adult)<br><br>Sex: All	•Meng Qiu<br>•West China Hospital	•Other	Study Start: June 30, 2021<br><br>Primary Completion: July 1, 2022<br><br>Study Completion: December 31, 2022<br><br>First Posted: August 4, 2021<br><br>Results First Posted: No Results Posted<br><br>Last Update Posted: October 15, 2021	•Meng Qiu, Sichuan, China

	NCT Number	Title	Other Names	Status	Conditions	Interventions	Characteristics	Population	Sponsor/ Collaborators	Funder Type	Dates	Locations
99	NCT04149717	The Effect of Energy Drink Ingredients on Cardiovascular Function in Men and Women 18-39 Years Old<br>Study Documents:	Title Acronym: EEDICF<br>Other Ids: #2019/10/1	Recruiting	•Cardiac Arrhythmia<br>•Blood Pressure	•Dietary Supplement: Energy Drink Ingredients and Exercise	Study Type: Interventional<br>Phase: Not Applicable<br>Study Design:<br>•Allocation: Randomized<br>•Intervention Model: Crossover Assignment<br>•Masking: Triple (Participant, Investigator, Outcomes Assessor)<br>•Primary Purpose: Basic Science<br>Outcome Measures:<br>•Change in QTc interval of EKG<br>•Change in Heart Rate<br>•Change in Systolic and Diastolic Blood Pressure	Enrollment: 35<br>Age: 18 Years to 39 Years (Adult)<br>Sex: All	•Duquesne University	•Other	Study Start: January 1, 2020<br>Primary Completion: December 2022<br>Study Completion: March 2023<br>First Posted: November 4, 2019<br>Results First Posted: No Results Posted<br>Last Update Posted: March 23, 2021	•Duquesne University, Pittsburgh, Pennsylvania, United States
100	NCT03533972	Effect of Ashwagandha on Salivary Antioxidant and Serum c Reactive Protein in Chronic Generalized Periodontitis<br>Study Documents:	Title Acronym:<br>Other Ids: nibhask	Completed	•Chronic Periodontitis	•Drug: Ashwagandha<br>•Other: Placebo	Study Type: Interventional<br>Phase:<br>•Phase 1<br>•Phase 2<br>Study Design:<br>•Allocation: Randomized<br>•Intervention Model: Parallel Assignment<br>•Masking: Double (Participant, Investigator)<br>•Primary Purpose: Treatment<br>Outcome Measures:<br>•change in c reactive protein<br>•change in super oxide dismutase<br>•change in probing depth<br>•change in clinical attachment loss<br>•gingival index	Enrollment: 50<br>Age: 17 Years to 55 Years (Child, Adult)<br>Sex: All	•Tatyasaheb Kore Dental College	•Other	Study Start: November 30, 2017<br>Primary Completion: February 27, 2018<br>Study Completion: April 28, 2018<br>First Posted: May 23, 2018<br>Results First Posted: No Results Posted<br>Last Update Posted: May 23, 2018	•Tatyasaheb Kore Dental College, Kolhapur, Maharashtra, India

44 additional studies not shown
